# Supplementary material for: No apparent influence of psychometrically-defined schizotypy on orientation-dependent contextual modulation of visual contrast detection
Source: PeerJ. 2017 Jan 24;5:e2921. doi: 10.7717/peerj.2921 (PMC5267566; doi:10.7717/peerj.2921)
Supplement: Figure S1 — The blue circles represent the proportion of correct responses within a given target contrast bin, with an area that is proportional to the number of trials. The solid black lines represent the best-fitting psychometric function, with the grey surrounding region capturing the bootstrapped 95% confidence interval. The dashed lines indicate the contrast detection threshold (the target contrast corresponding to 69.25% accuracy). The vertical axes are accuracy (proportion correct) and the horizontal axes are the target contrast (logarithmic spacing). The rows depict the four experimental conditions. [file peerj-05-2921-s001.pdf]

Fig. S 1 Raw data and best-fitting psychometric functions for all participants (begins overleaf). The blue circles represent the proportion of correct responses within a given target contrast bin, with an area that is proportional to the number of trials. The solid black lines represent the best-fitting psychometric function, with the grey surrounding region capturing the bootstrapped 95% confidence interval. The dashed lines indicate the contrast detection threshold (the target contrast corresponding to 69.25% accuracy). The vertical axes are accuracy (proportion correct) and the horizontal axes are the target contrast (logarithmic spacing). The rows depict the four experimental conditions.

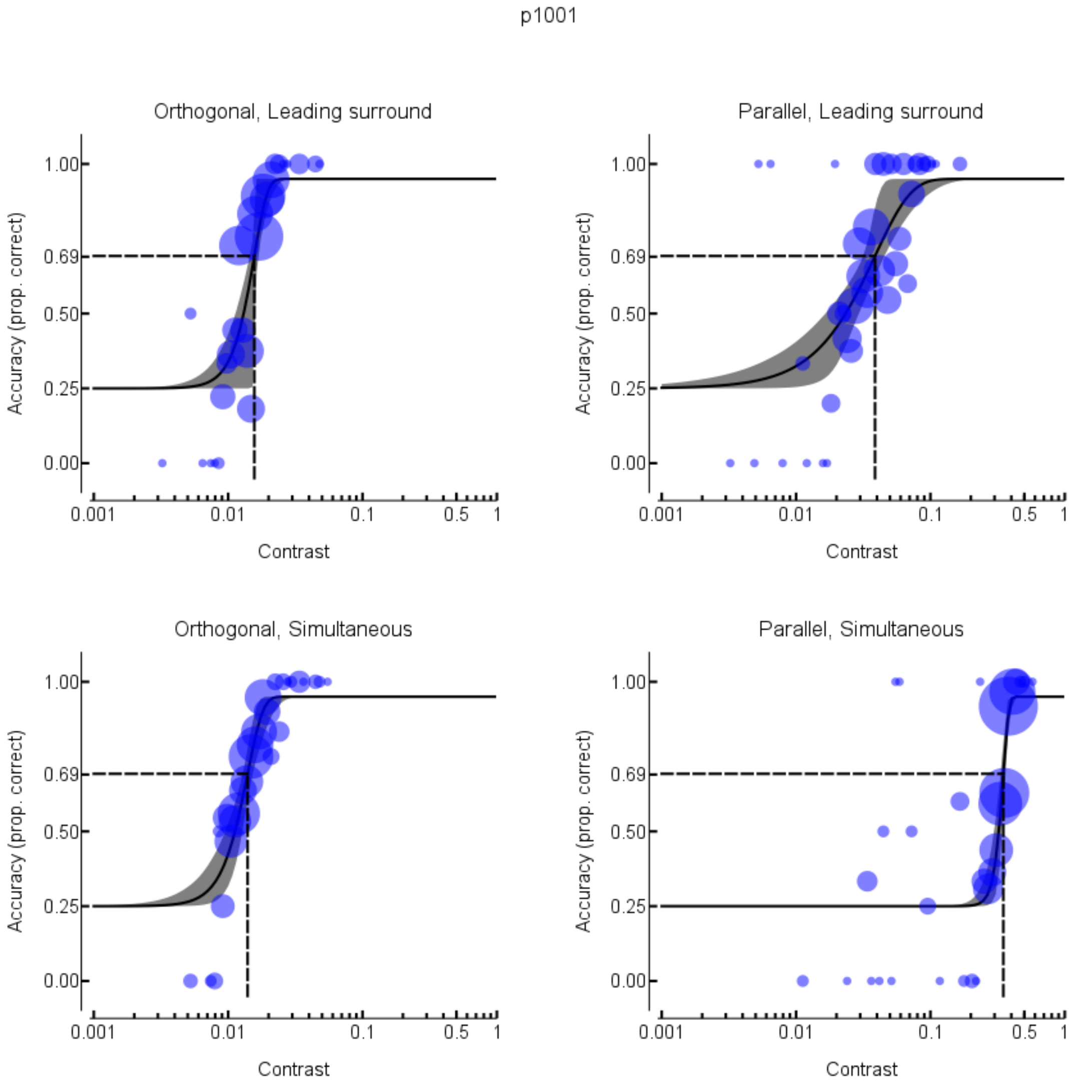
p1001
Orthogonal, Leading surround
Parallel, Leading surround
Orthogonal, Simultaneous
Parallel, Simultaneous
Accuracy (prop. correct)
1.00
0.69
0.50
0.25
0.00
0.001
0.01
0.1
0.5
1
Contrast

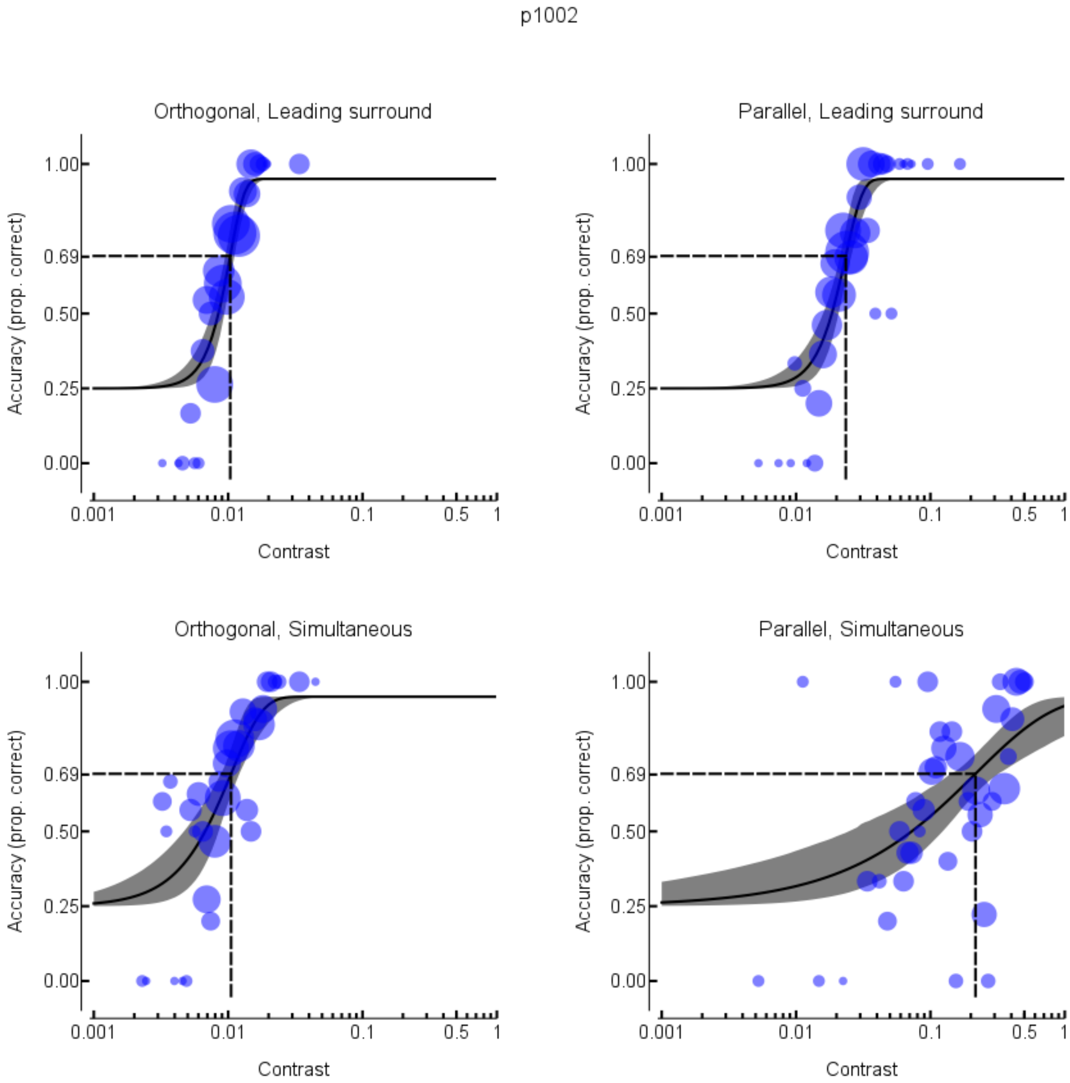
p1002
Orthogonal, Leading surround
Parallel, Leading surround
Orthogonal, Simultaneous
Parallel, Simultaneous
Accuracy (prop. correct)
1.00
0.69
0.50
0.25
0.00
0.001
0.01
0.1
0.5
1
Contrast

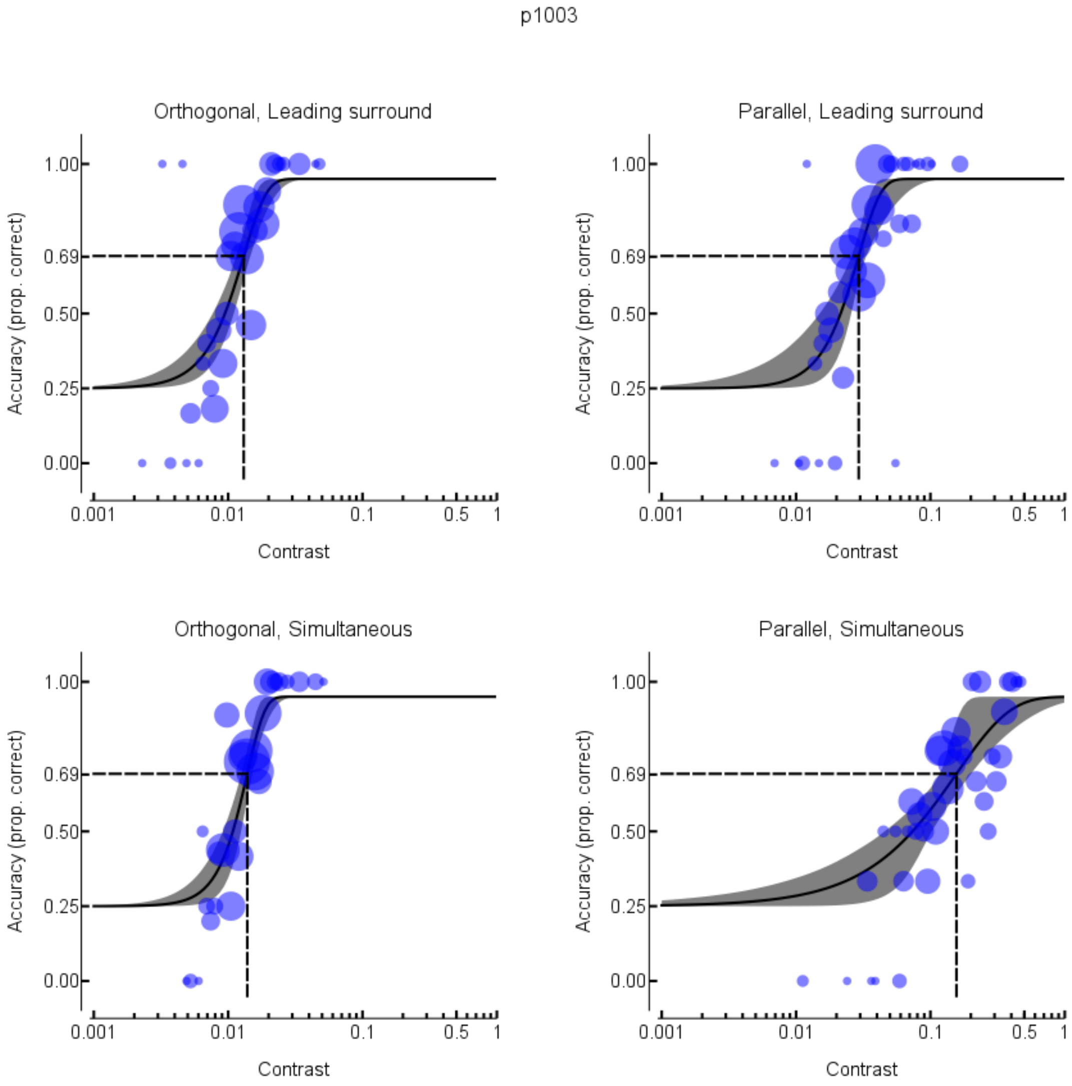
p1003
Orthogonal, Leading surround
Parallel, Leading surround
Orthogonal, Simultaneous
Parallel, Simultaneous
Accuracy (prop. correct)
1.00
0.69
0.50
0.25
0.00
0.001
0.01
0.1
0.5
1
Contrast

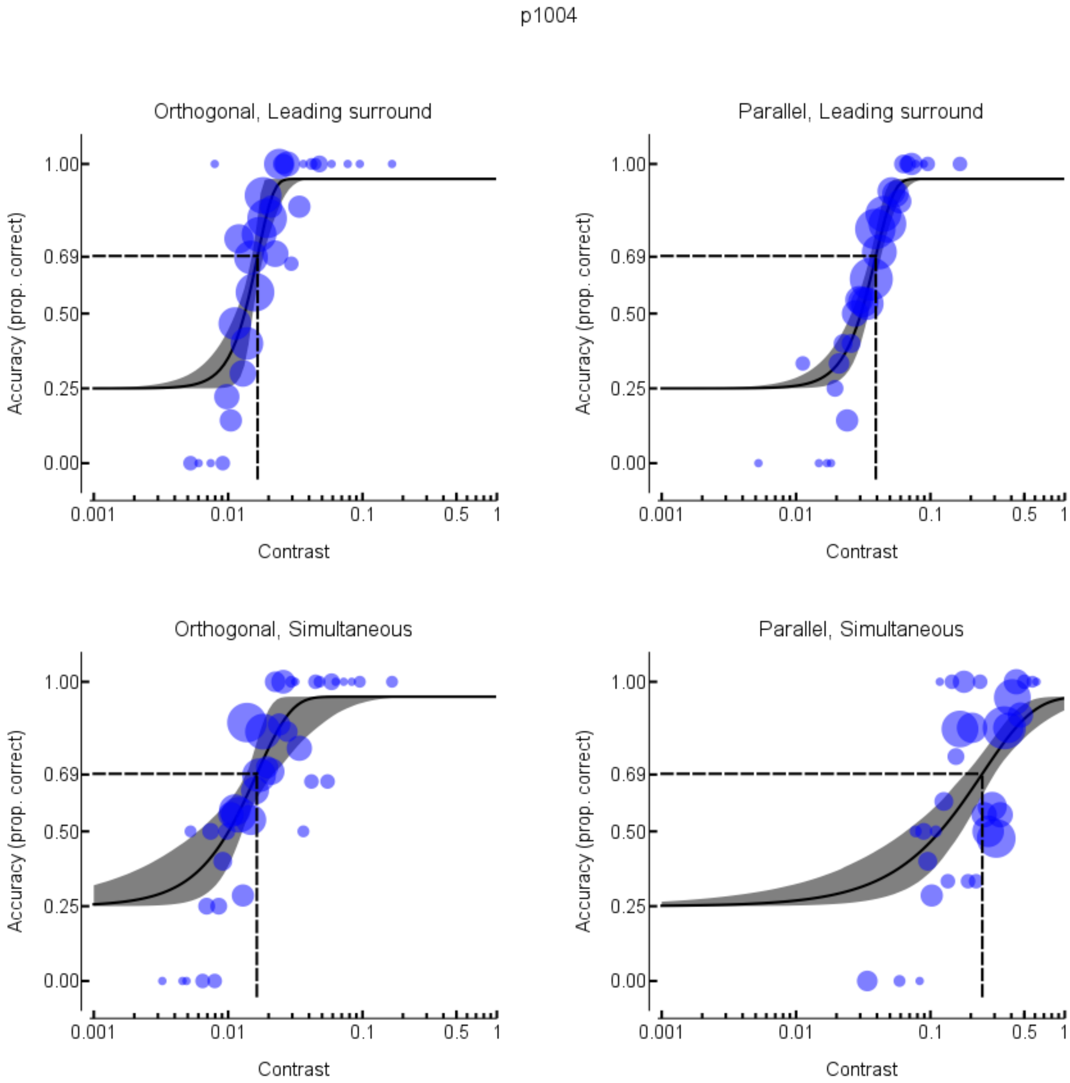
p1004
Orthogonal, Leading surround
Parallel, Leading surround
Orthogonal, Simultaneous
Parallel, Simultaneous
Accuracy (prop. correct)
1.00
0.69
0.50
0.25
0.00
0.001
0.01
0.1
0.5
1
Contrast

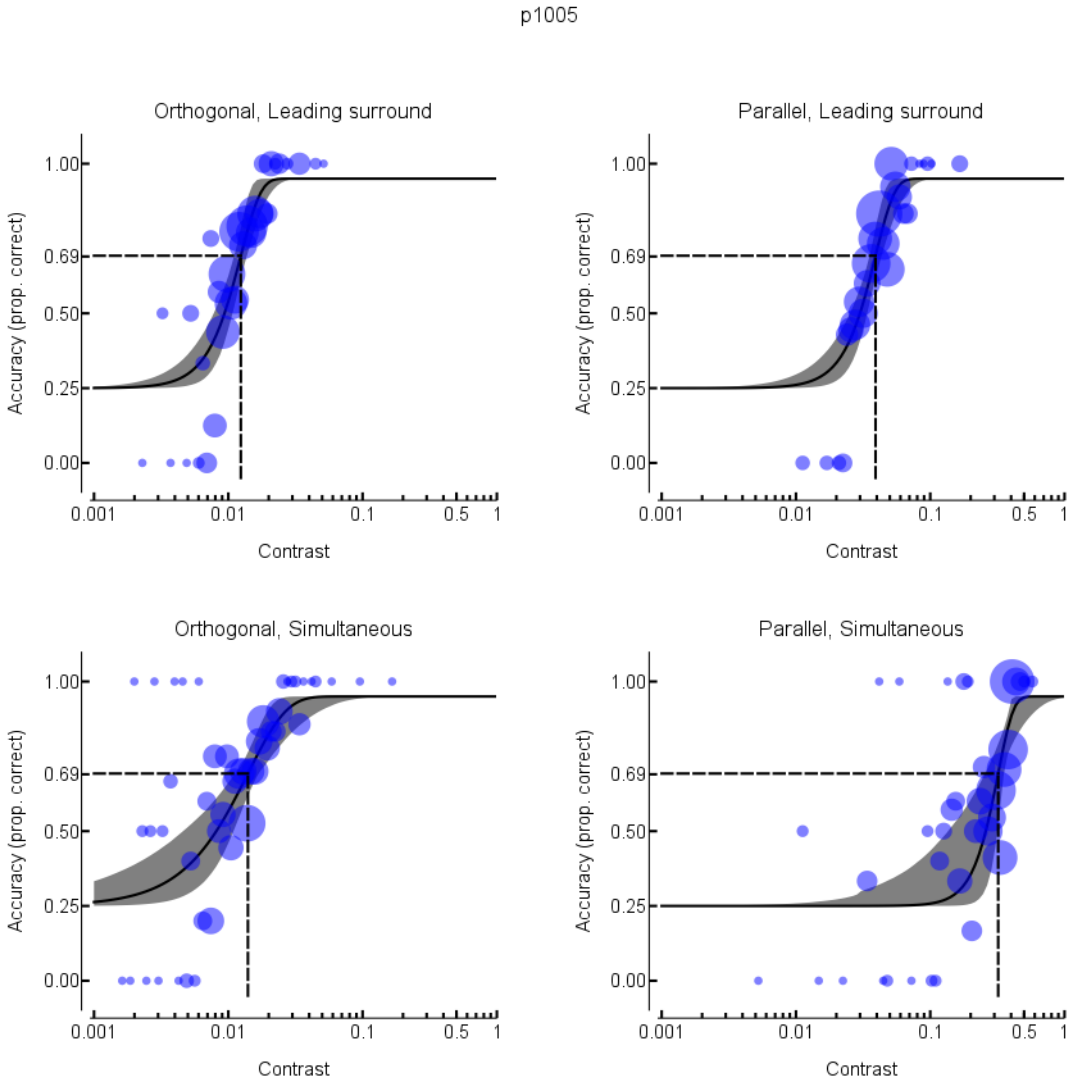
p1005
Orthogonal, Leading surround
Parallel, Leading surround
Orthogonal, Simultaneous
Parallel, Simultaneous
Accuracy (prop. correct)
1.00
0.69
0.50
0.25
0.00
0.001
0.01
0.1
0.5
1
Contrast

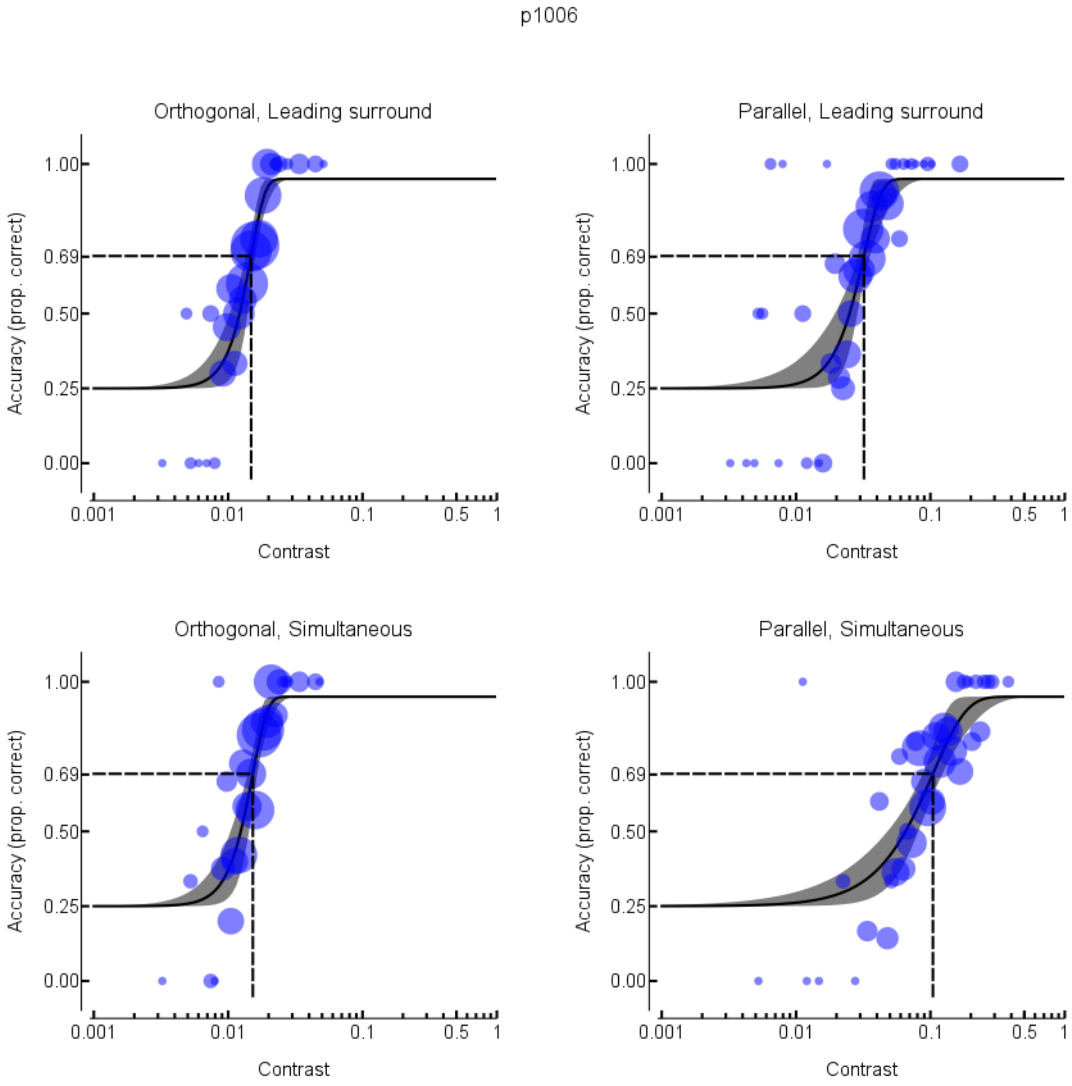
p1006
Orthogonal, Leading surround
Parallel, Leading surround
Orthogonal, Simultaneous
Parallel, Simultaneous
Accuracy (prop. correct)
1.00
0.69
0.50
0.25
0.00
Contrast
0.001
0.01
0.1
0.5
1

p1007

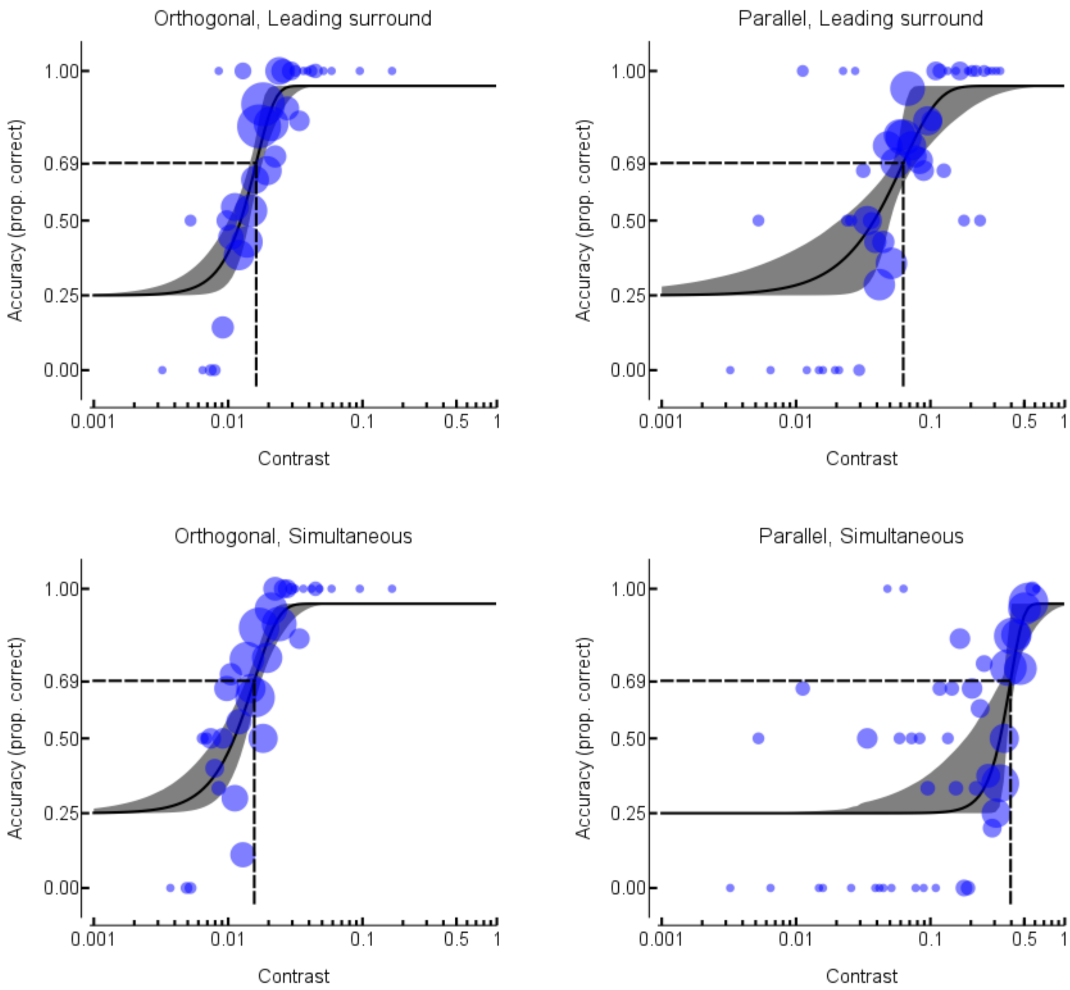
Orthogonal, Leading surround
Parallel, Leading surround
Orthogonal, Simultaneous
Parallel, Simultaneous
Accuracy (prop. correct)
1.00
0.69
0.50
0.25
0.00
0.001
0.01
0.1
0.5
1
Contrast

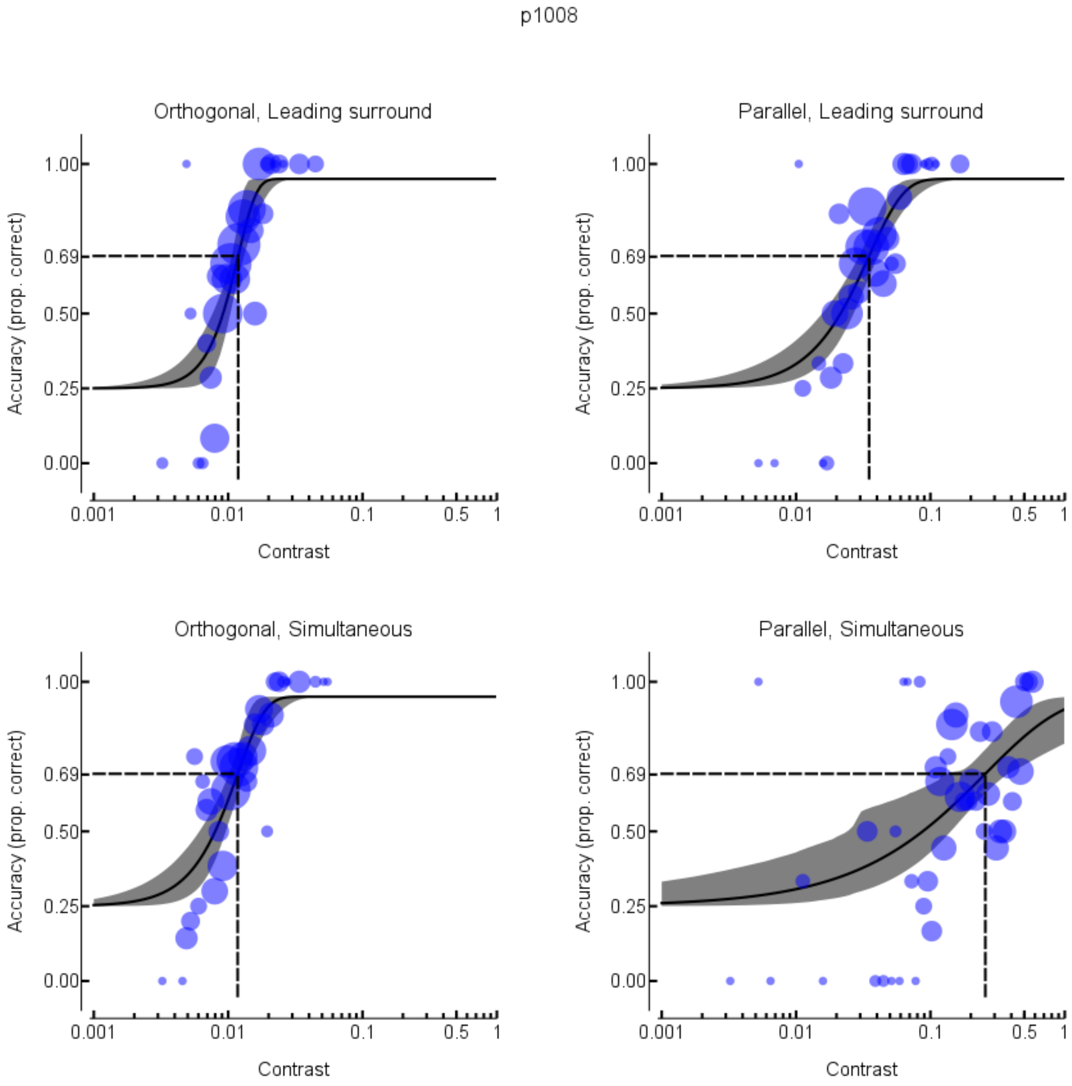
p1008
Orthogonal, Leading surround
Parallel, Leading surround
Orthogonal, Simultaneous
Parallel, Simultaneous
Accuracy (prop. correct)
1.00
0.69
0.50
0.25
0.00
Contrast
0.001
0.01
0.1
0.5
1

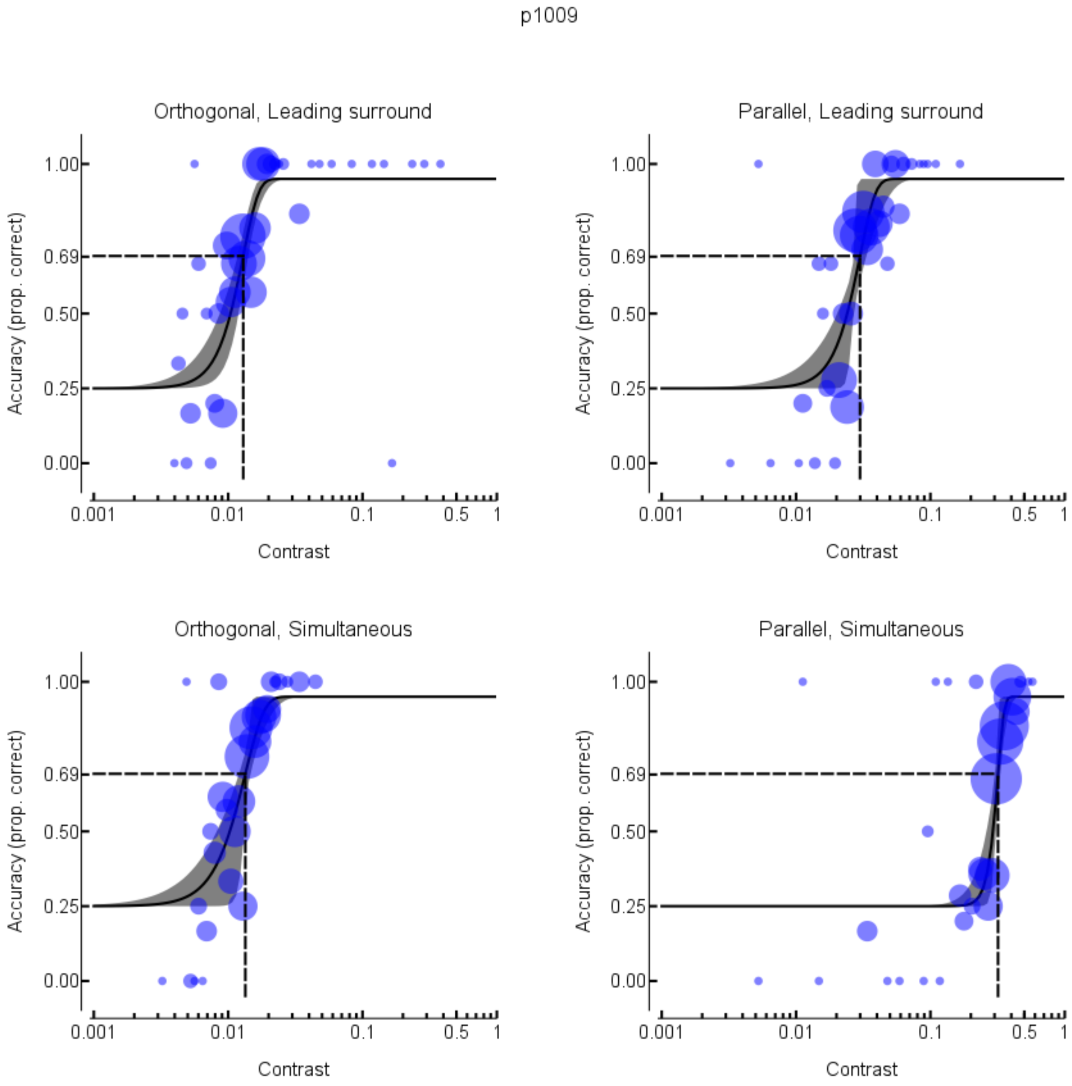
p1009
Orthogonal, Leading surround
Parallel, Leading surround
Orthogonal, Simultaneous
Parallel, Simultaneous
Accuracy (prop. correct)
1.00
0.69
0.50
0.25
0.00
0.001
0.01
0.1
0.5
1
Contrast

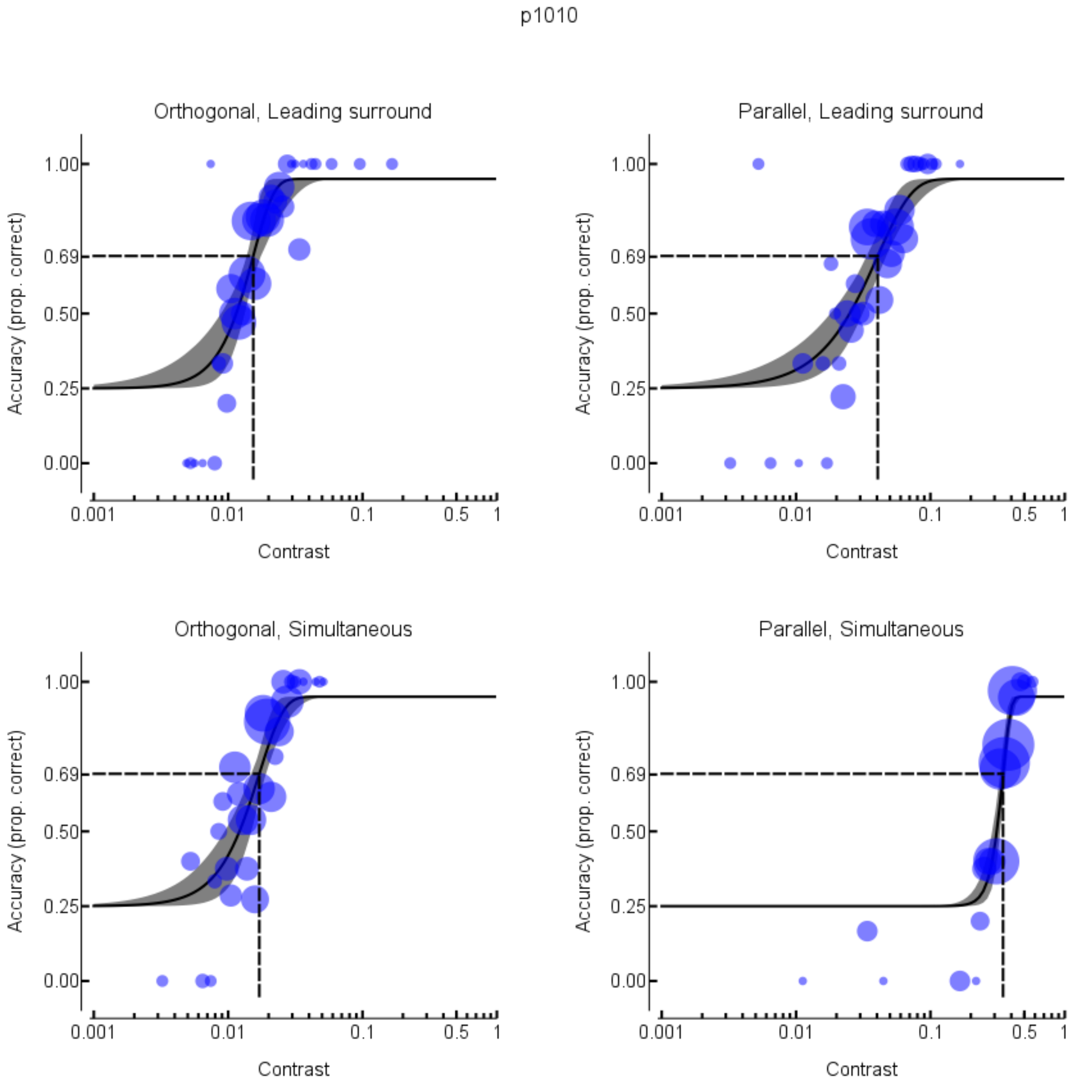
p1010
Orthogonal, Leading surround
Parallel, Leading surround
Orthogonal, Simultaneous
Parallel, Simultaneous
Accuracy (prop. correct)
1.00
0.69
0.50
0.25
0.00
Contrast
0.001
0.01
0.1
0.5
1

p1011

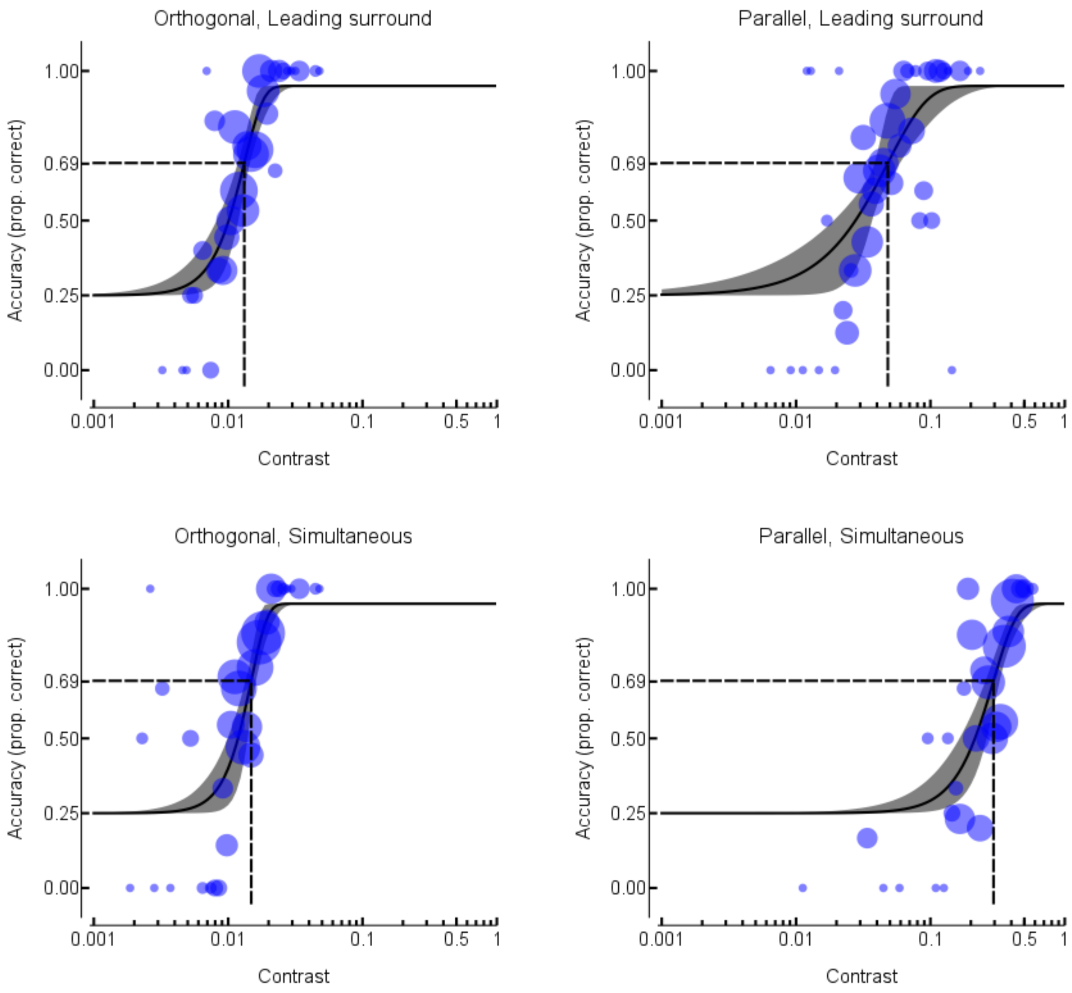
Orthogonal, Leading surround
Parallel, Leading surround
Orthogonal, Simultaneous
Parallel, Simultaneous
Accuracy (prop. correct)
1.00
0.69
0.50
0.25
0.00
Contrast
0.001
0.01
0.1
0.5
1

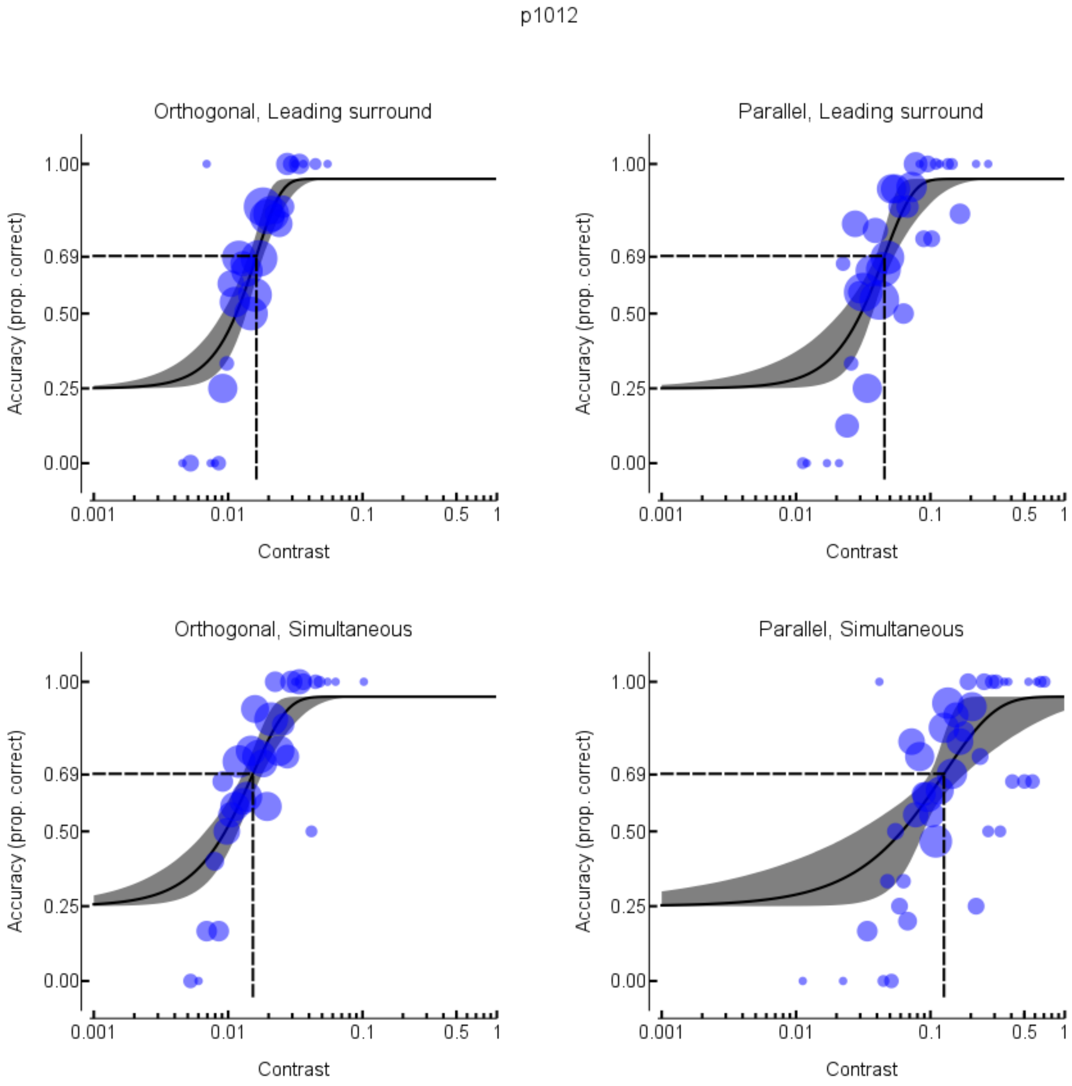
p1012
Orthogonal, Leading surround
Parallel, Leading surround
Orthogonal, Simultaneous
Parallel, Simultaneous
Accuracy (prop. correct)
1.00
0.69
0.50
0.25
0.00
0.001
0.01
0.1
0.5
1
Contrast

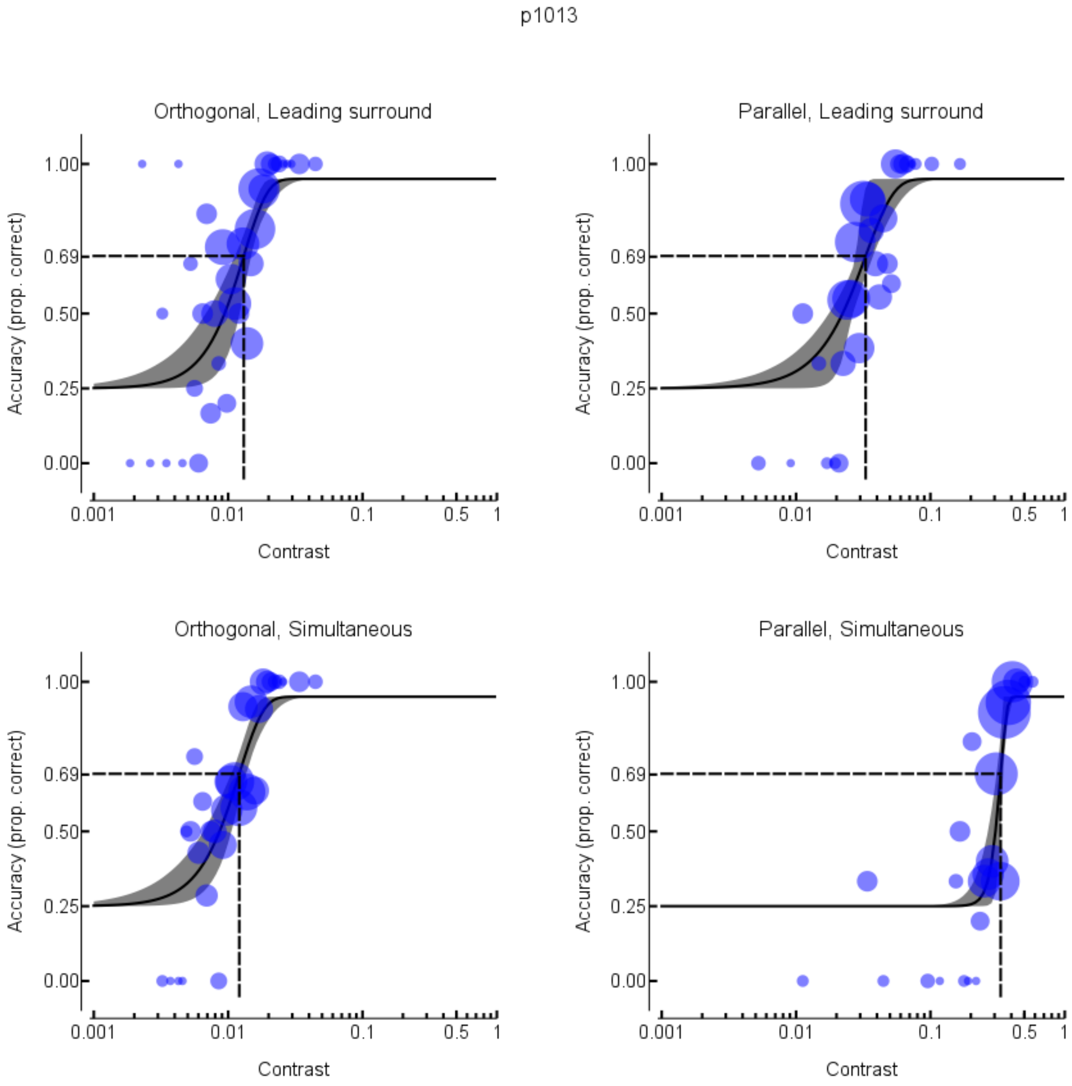
p1013
Orthogonal, Leading surround
Parallel, Leading surround
Orthogonal, Simultaneous
Parallel, Simultaneous
Accuracy (prop. correct)
1.00
0.69
0.50
0.25
0.00
0.001
0.01
0.1
0.5
1
Contrast

p1014

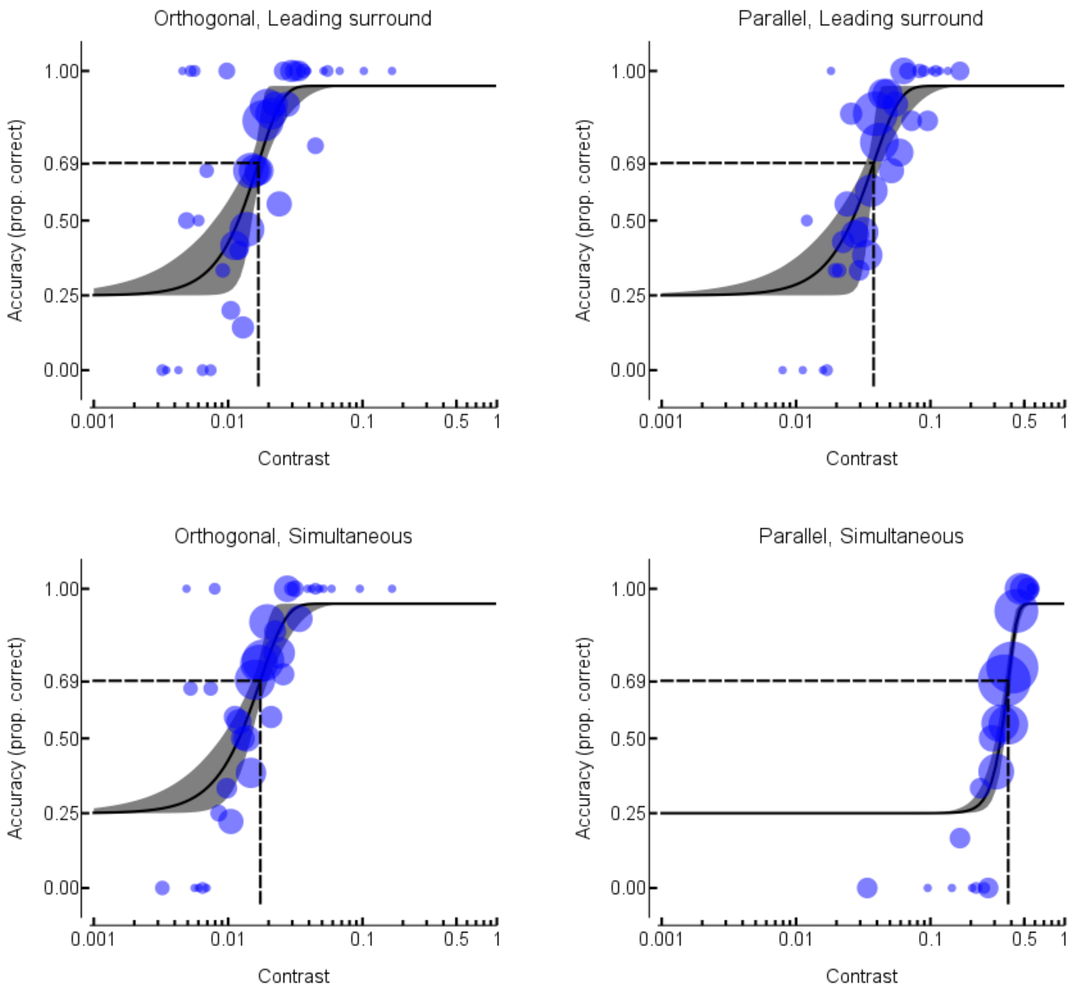
Orthogonal, Leading surround
Parallel, Leading surround
Orthogonal, Simultaneous
Parallel, Simultaneous
Accuracy (prop. correct)
1.00
0.69
0.50
0.25
0.00
0.001
0.01
0.1
0.5
1
Contrast

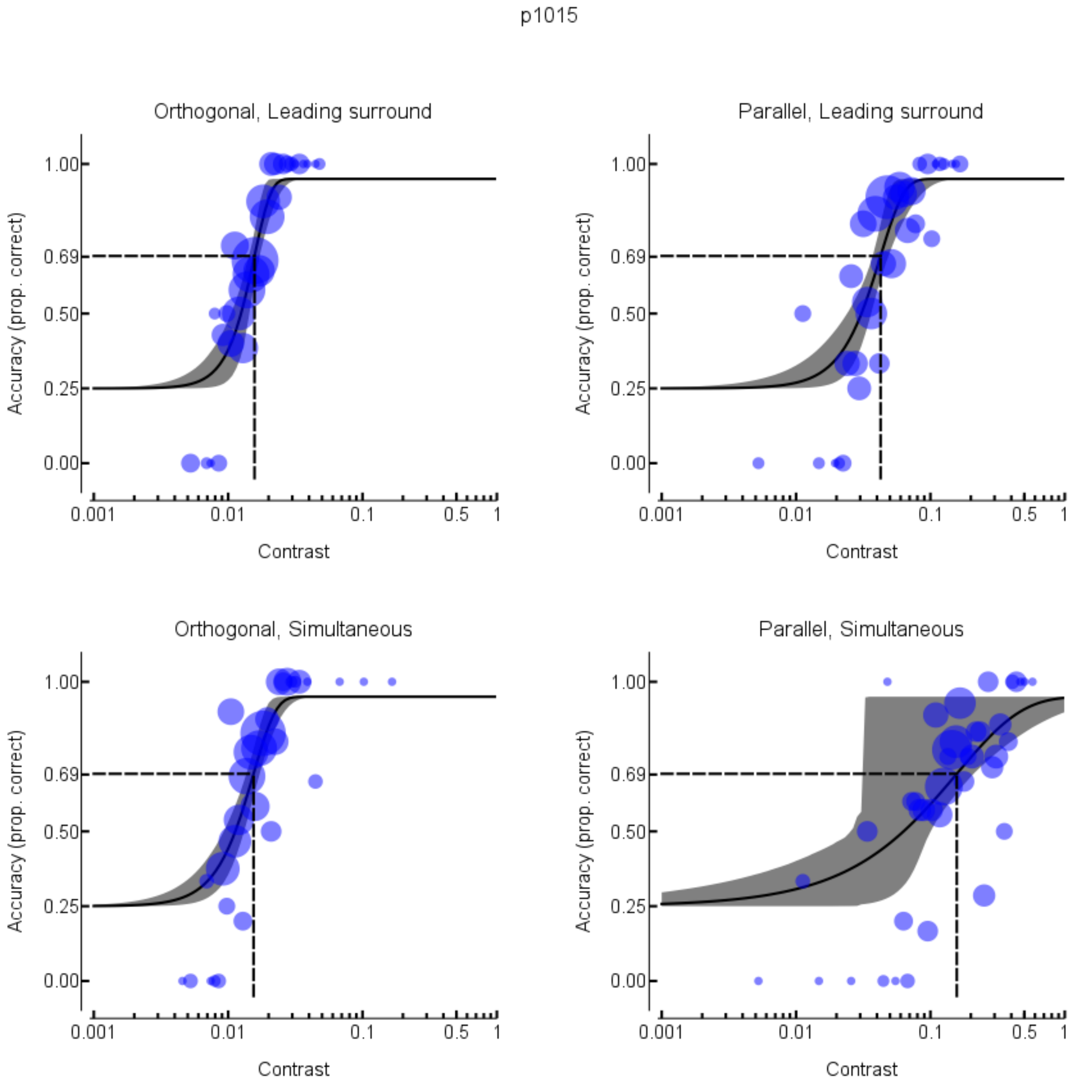
p1015
Orthogonal, Leading surround
Parallel, Leading surround
Orthogonal, Simultaneous
Parallel, Simultaneous
Accuracy (prop. correct)
1.00
0.69
0.50
0.25
0.00
0.001
0.01
0.1
0.5
1
Contrast

p1016

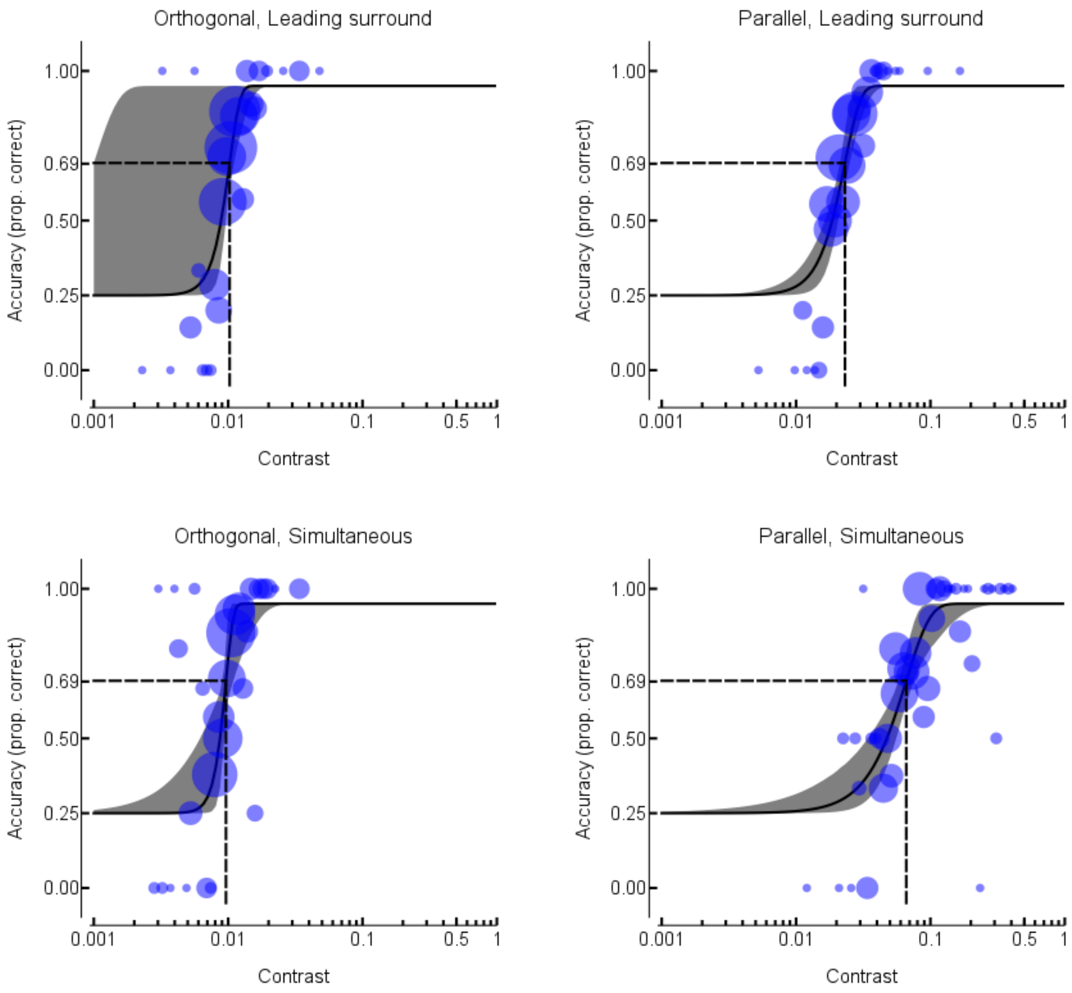
Orthogonal, Leading surround
Parallel, Leading surround
Orthogonal, Simultaneous
Parallel, Simultaneous
Accuracy (prop. correct)
1.00
0.69
0.50
0.25
0.00
0.001
0.01
0.1
0.5
1
Contrast

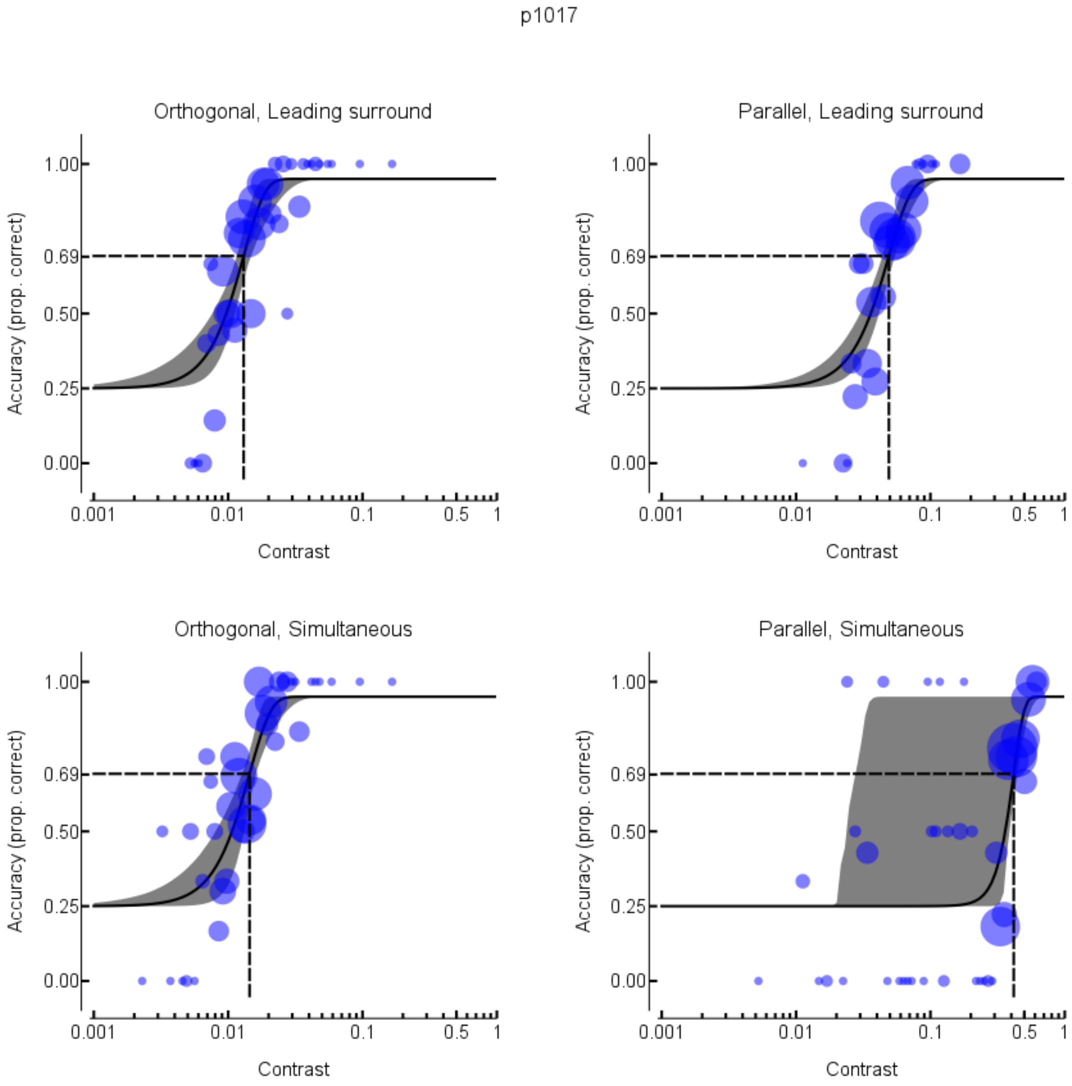
p1017
Orthogonal, Leading surround
Parallel, Leading surround
Orthogonal, Simultaneous
Parallel, Simultaneous
Accuracy (prop. correct)
1.00
0.69
0.50
0.25
0.00
0.001
0.01
0.1
0.5
1
Contrast

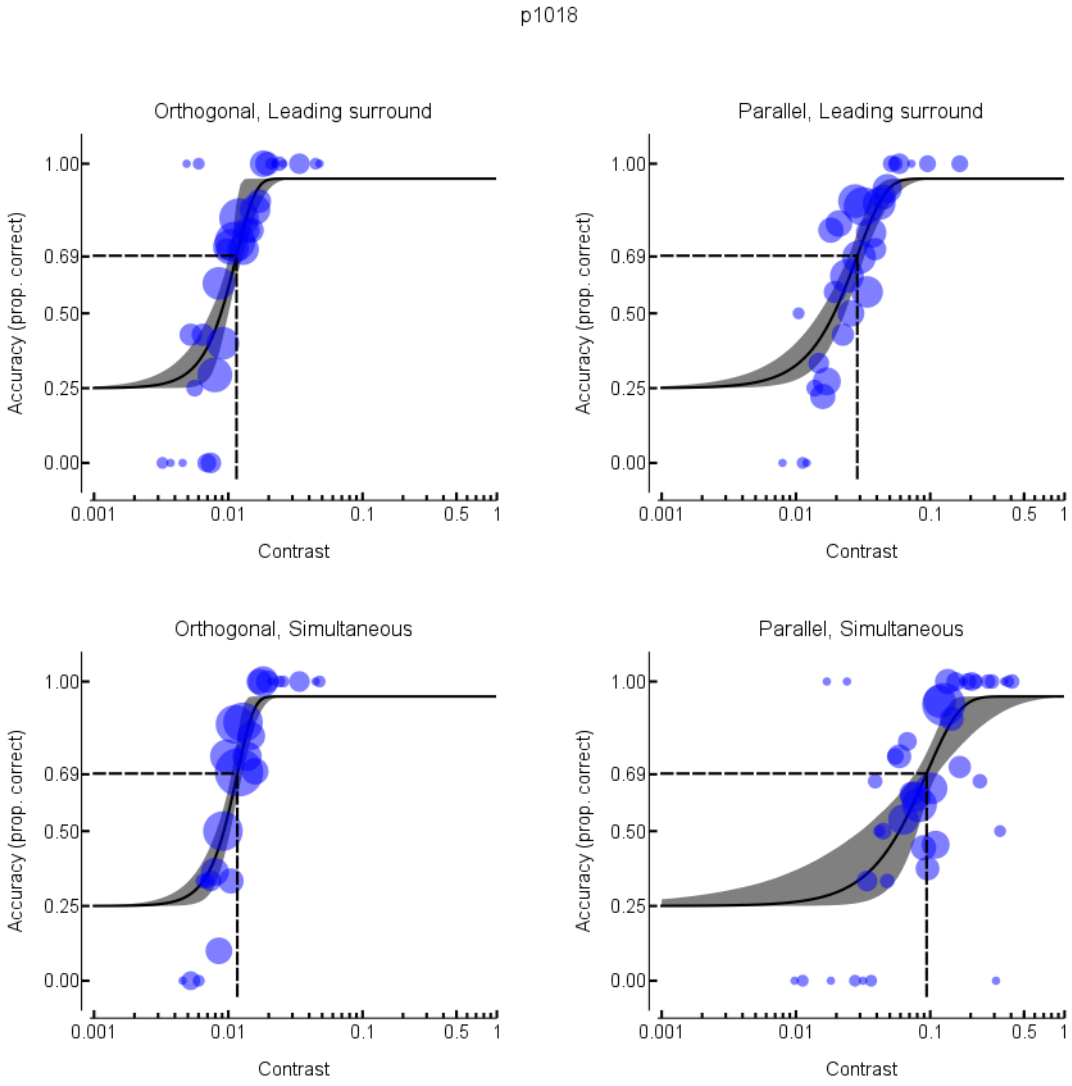
p1018
Orthogonal, Leading surround
Parallel, Leading surround
Orthogonal, Simultaneous
Parallel, Simultaneous
Accuracy (prop. correct)
1.00
0.69
0.50
0.25
0.00
0.001
0.01
0.1
0.5
1
Contrast

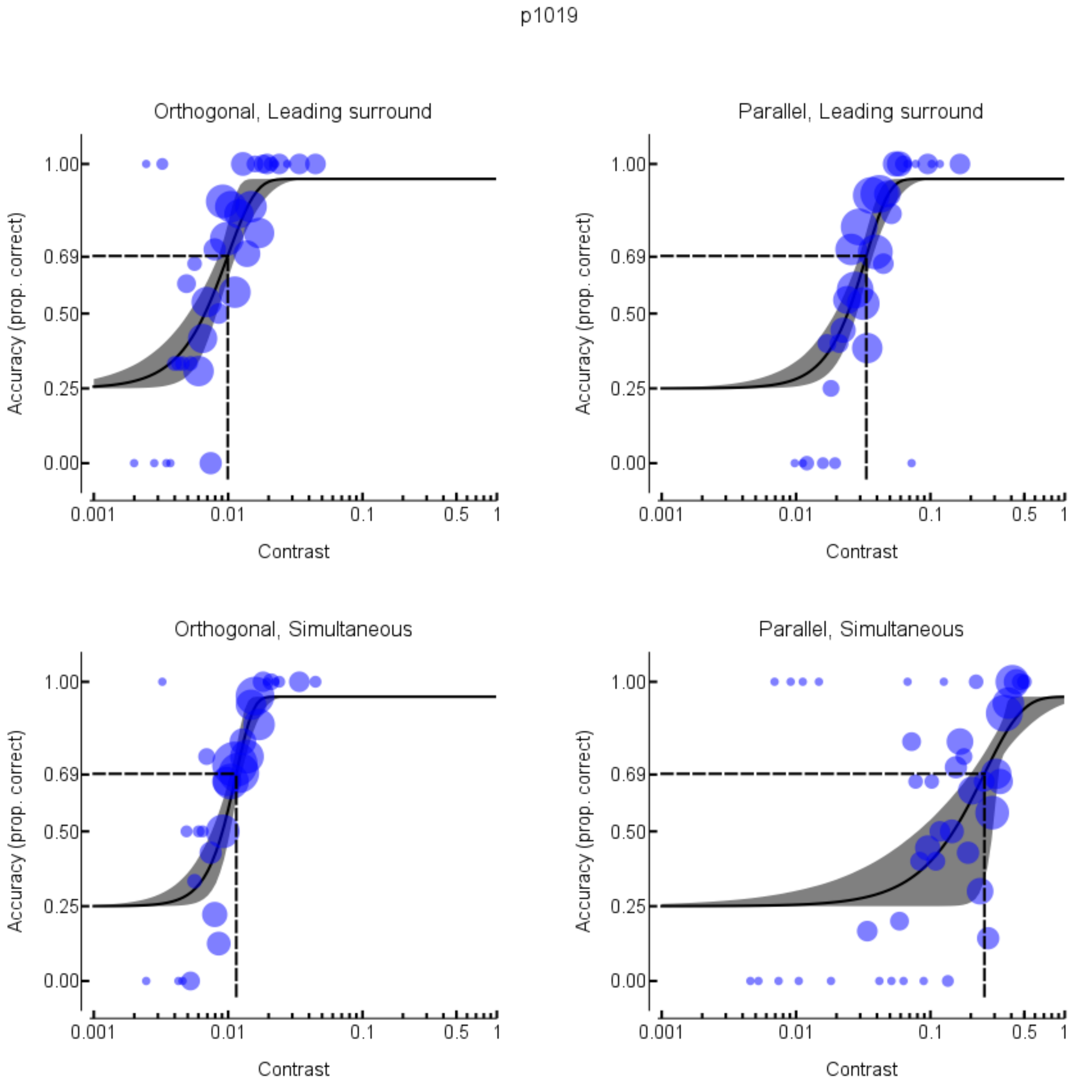
p1019
Orthogonal, Leading surround
Parallel, Leading surround
Orthogonal, Simultaneous
Parallel, Simultaneous
Accuracy (prop. correct)
1.00
0.69
0.50
0.25
0.00
Contrast
0.001
0.01
0.1
0.5
1

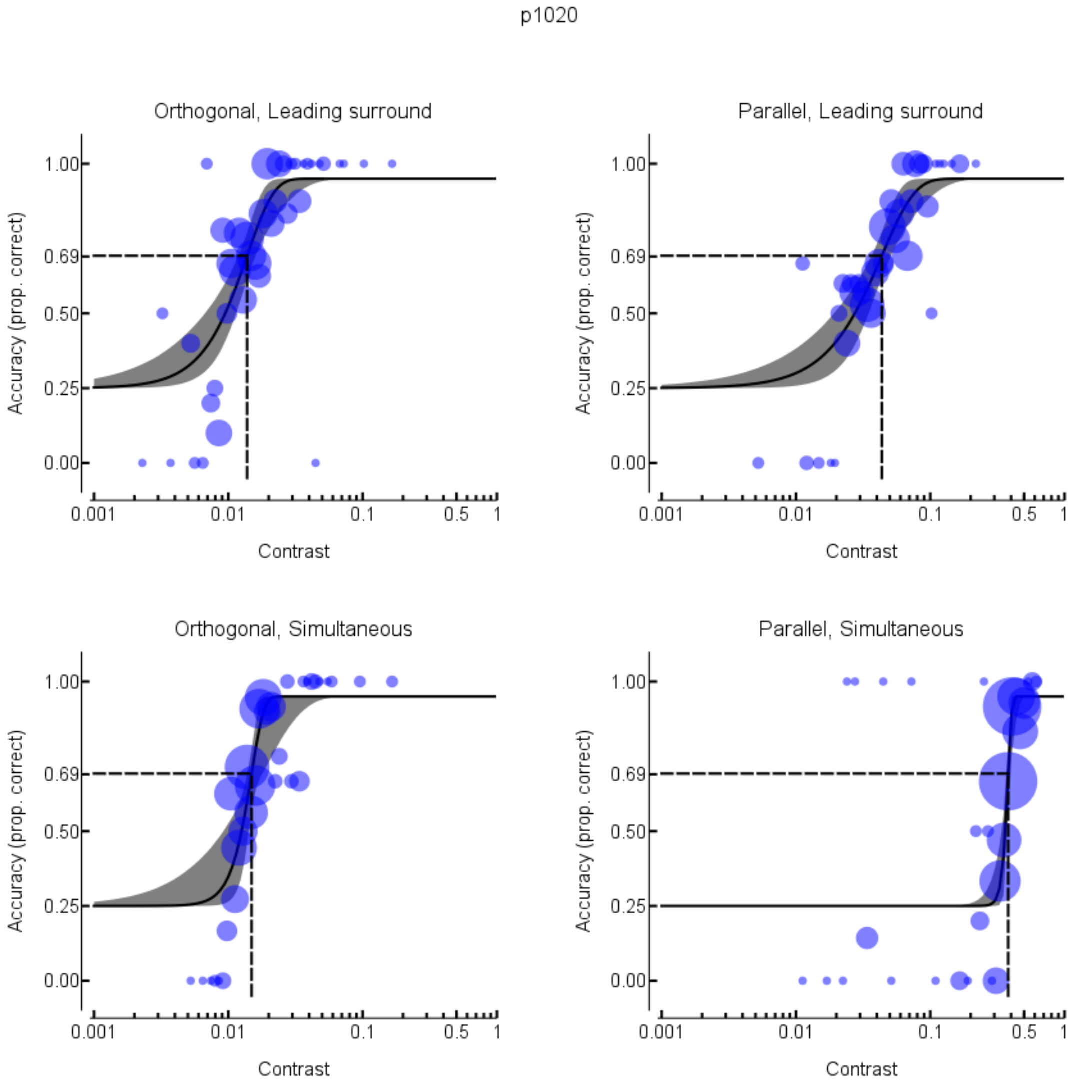
p1020
Orthogonal, Leading surround
Parallel, Leading surround
Orthogonal, Simultaneous
Parallel, Simultaneous
Accuracy (prop. correct)
1.00
0.69
0.50
0.25
0.00
0.001
0.01
0.1
0.5
1
Contrast

p1021

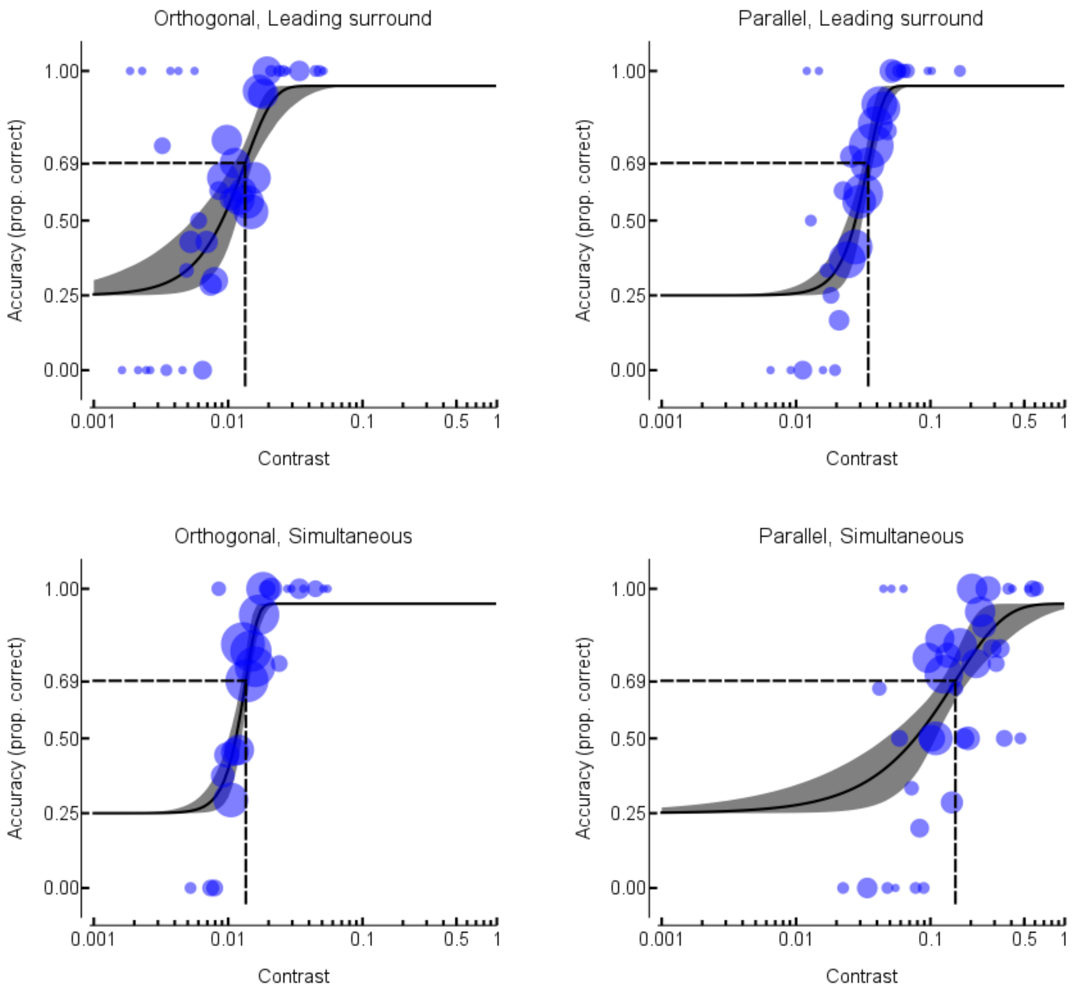

Orthogonal, Leading surround
Parallel, Leading surround
Orthogonal, Simultaneous
Parallel, Simultaneous
Accuracy (prop. correct)
1.00
0.69
0.50
0.25
0.00
Contrast
0.001
0.01
0.1
0.5
1

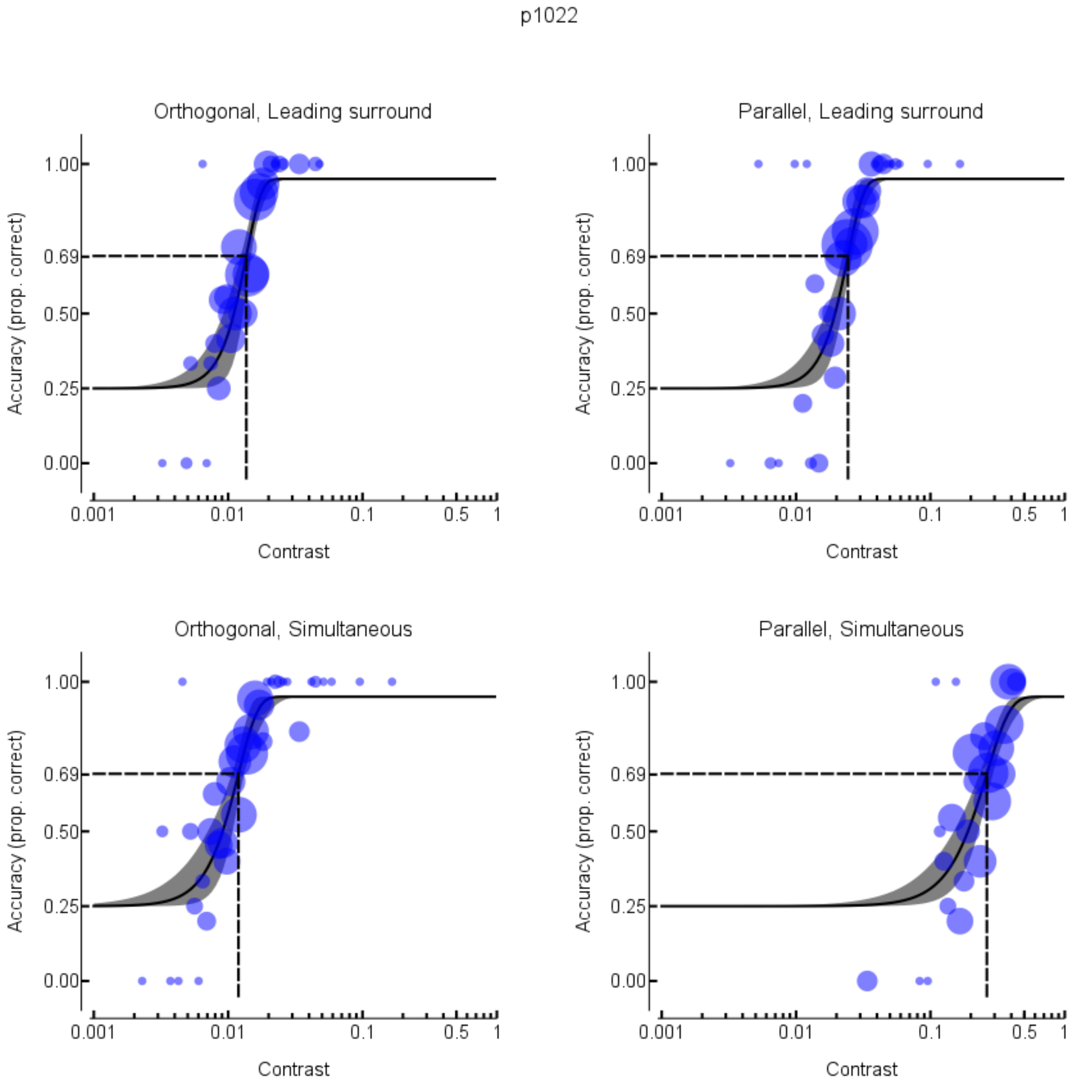
p1022
Orthogonal, Leading surround
Parallel, Leading surround
Orthogonal, Simultaneous
Parallel, Simultaneous
Accuracy (prop. correct)
1.00
0.69
0.50
0.25
0.00
0.001
0.01
0.1
0.5
1
Contrast

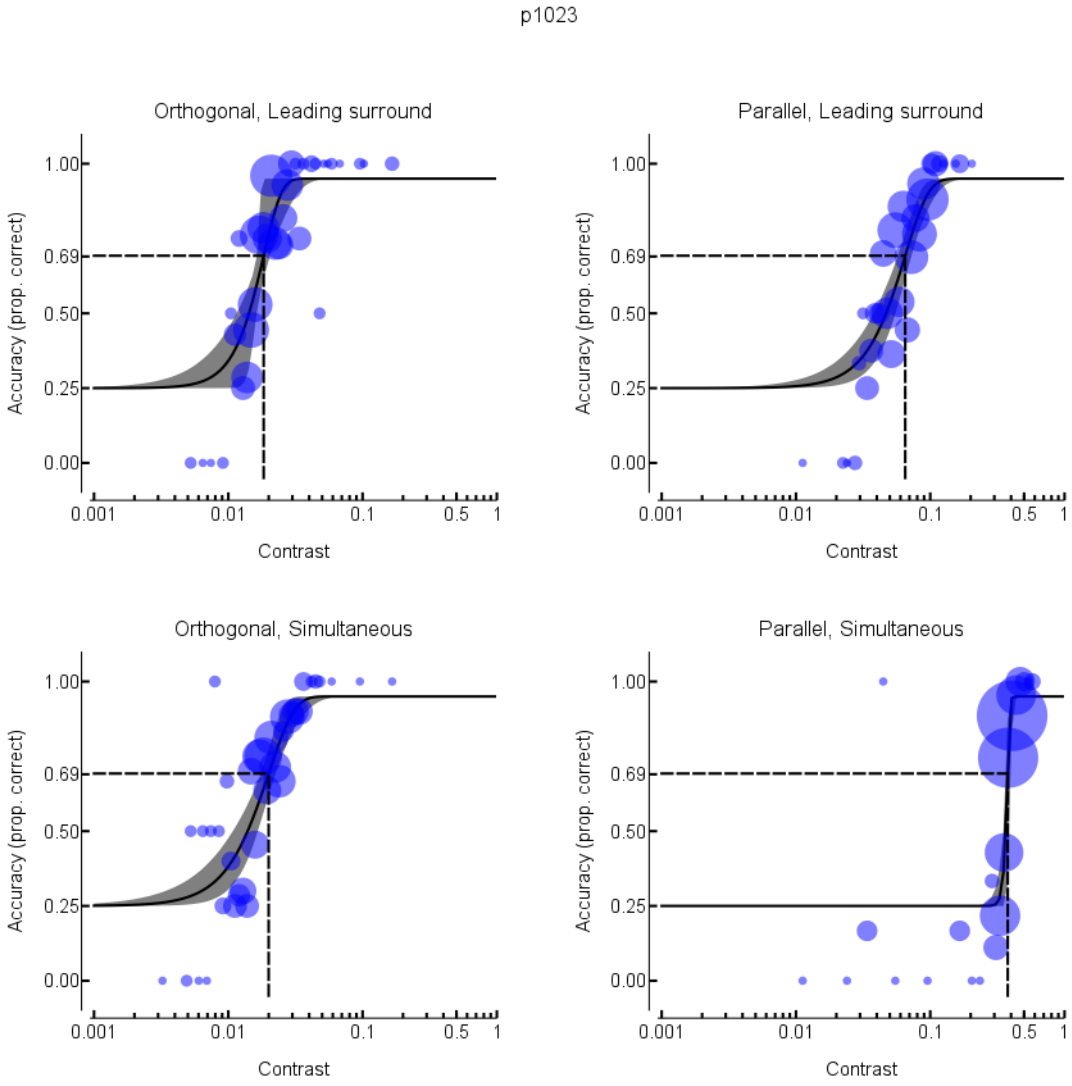
p1023
Orthogonal, Leading surround
Parallel, Leading surround
Orthogonal, Simultaneous
Parallel, Simultaneous
Accuracy (prop. correct)
1.00
0.69
0.50
0.25
0.00
Contrast
0.001
0.01
0.1
0.5
1

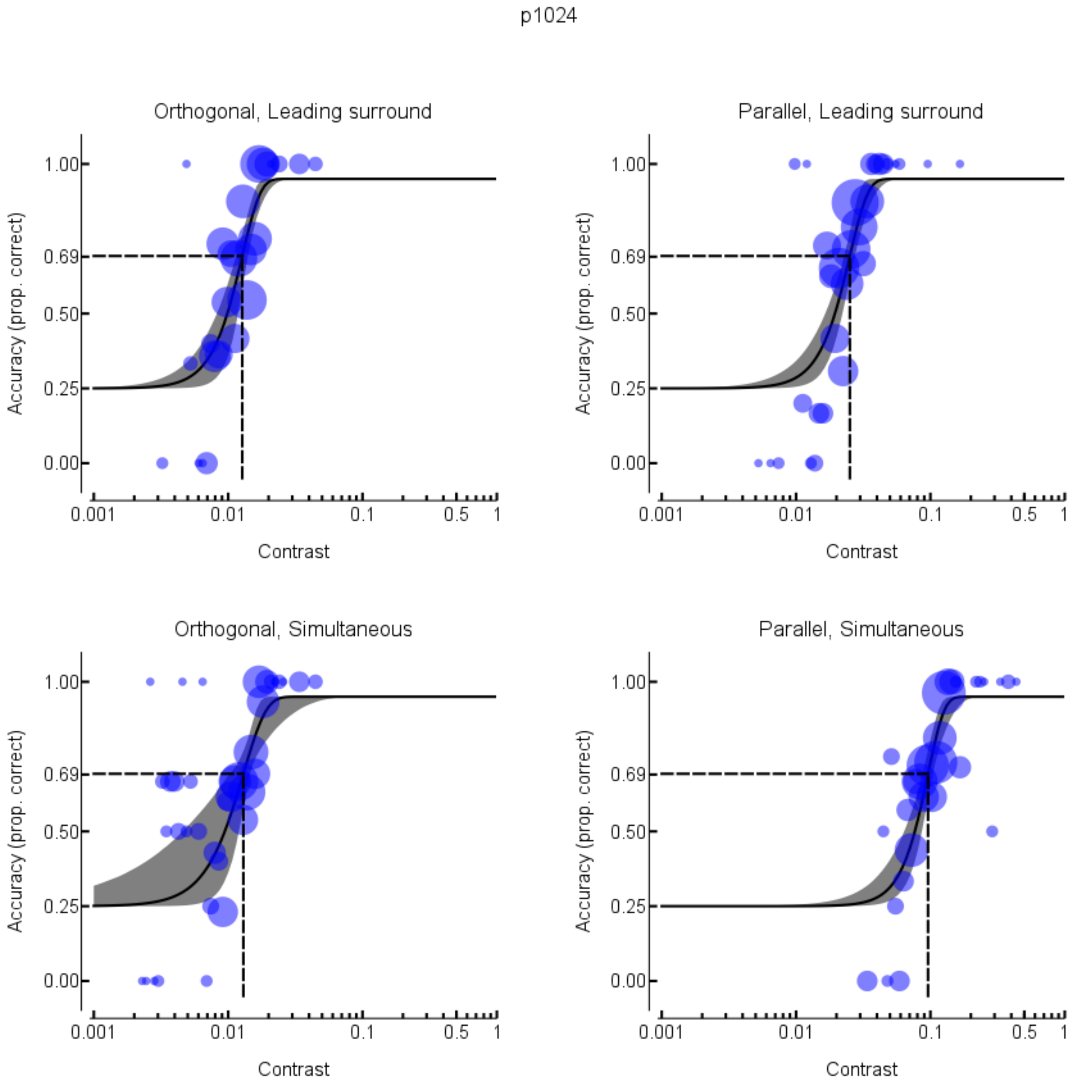
p1024
Orthogonal, Leading surround
Parallel, Leading surround
Orthogonal, Simultaneous
Parallel, Simultaneous
Accuracy (prop. correct)
1.00
0.69
0.50
0.25
0.00
Contrast
0.001
0.01
0.1
0.5
1

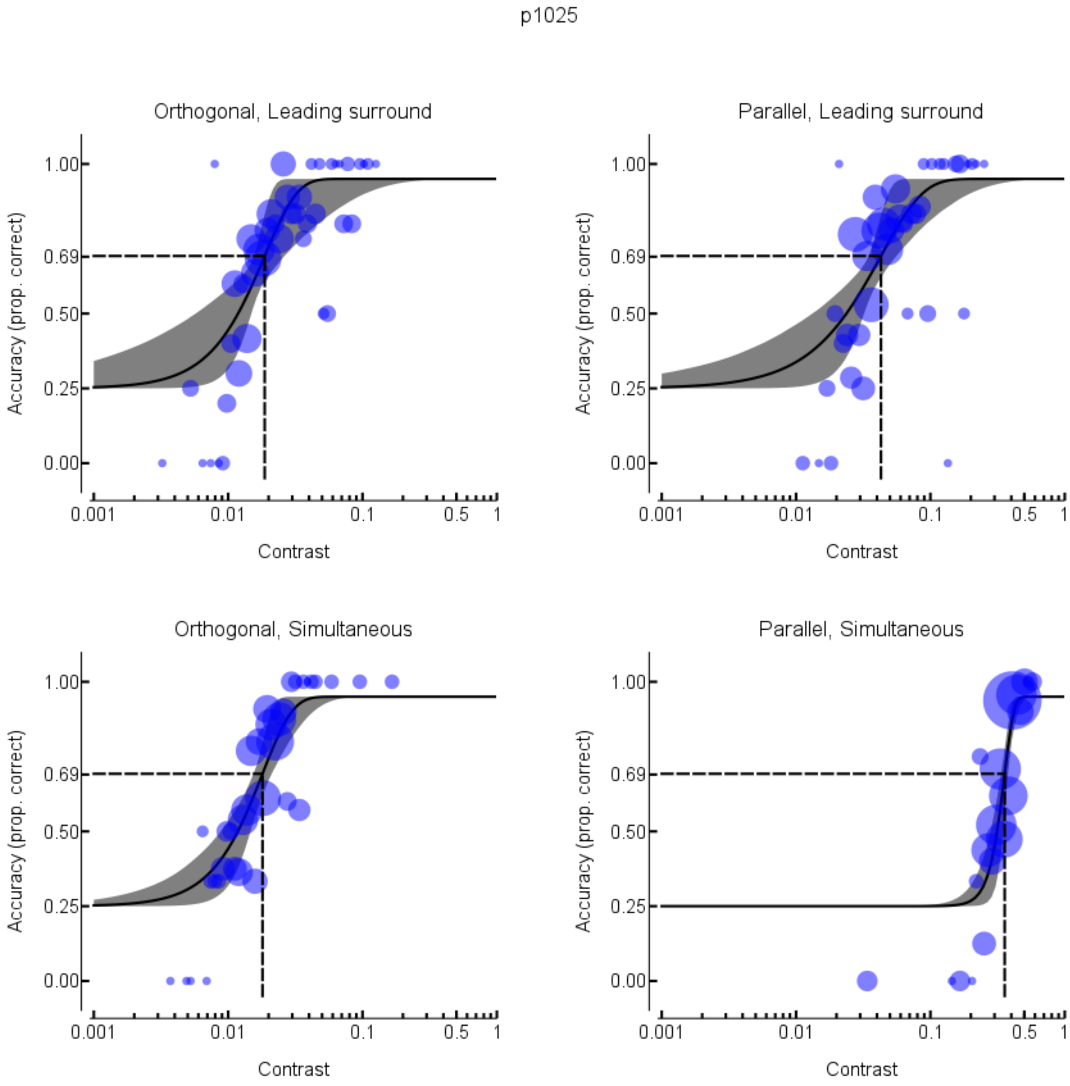
p1025
Orthogonal, Leading surround
Parallel, Leading surround
Orthogonal, Simultaneous
Parallel, Simultaneous
Accuracy (prop. correct)
1.00
0.69
0.50
0.25
0.00
0.001
0.01
0.1
0.5
1
Contrast

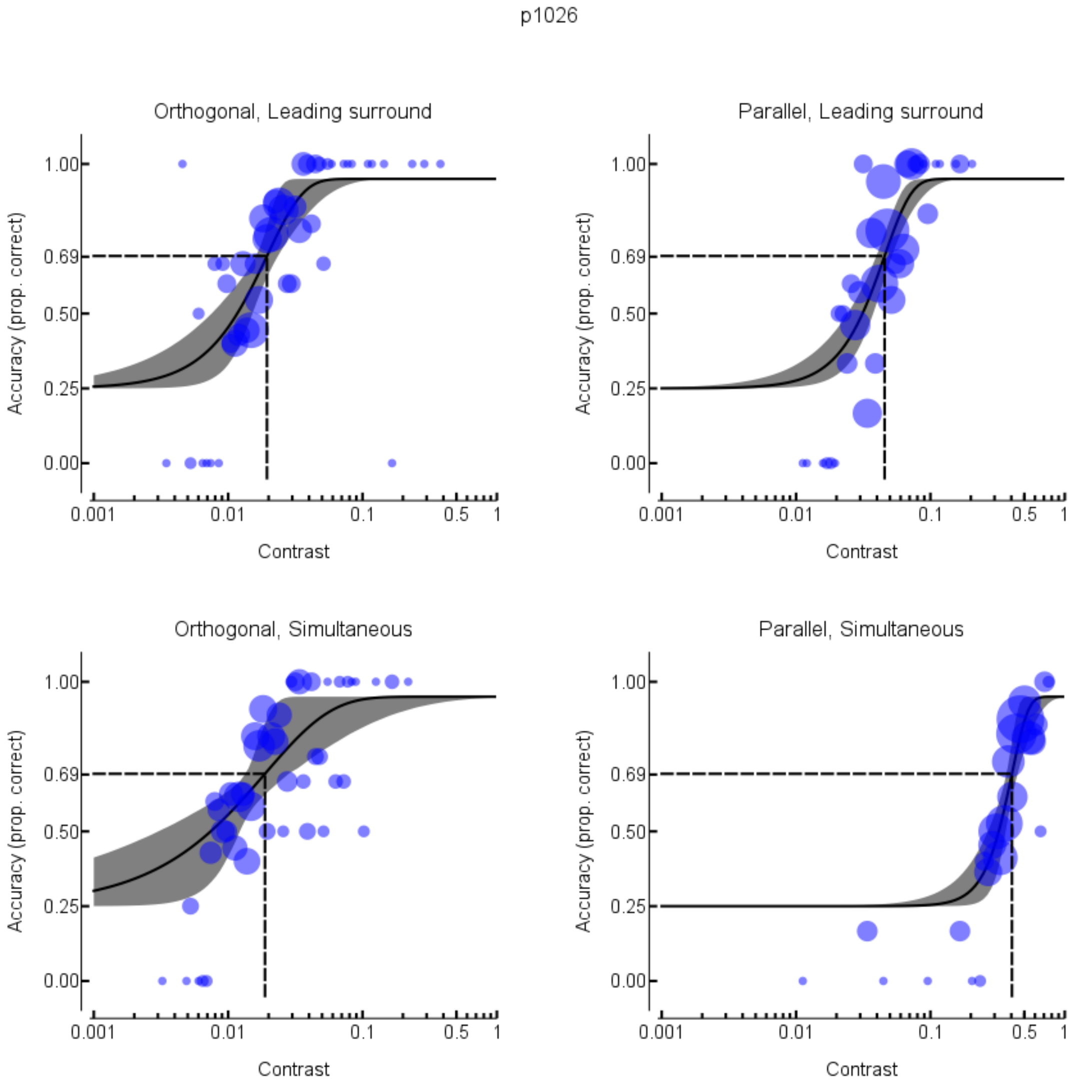
p1026
Orthogonal, Leading surround
Parallel, Leading surround
Orthogonal, Simultaneous
Parallel, Simultaneous
Accuracy (prop. correct)
1.00
0.69
0.50
0.25
0.00
Contrast
0.001
0.01
0.1
0.5
1

p1027 (excluded)

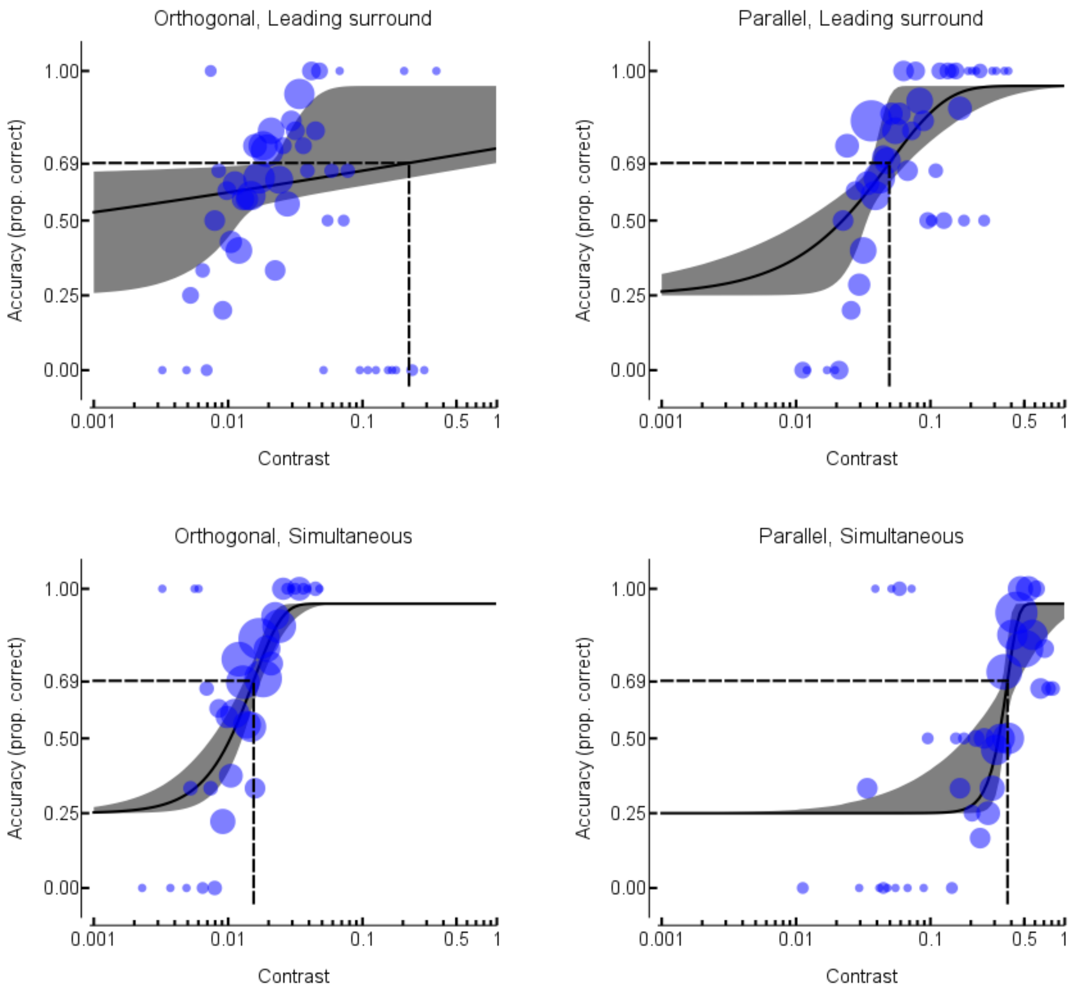

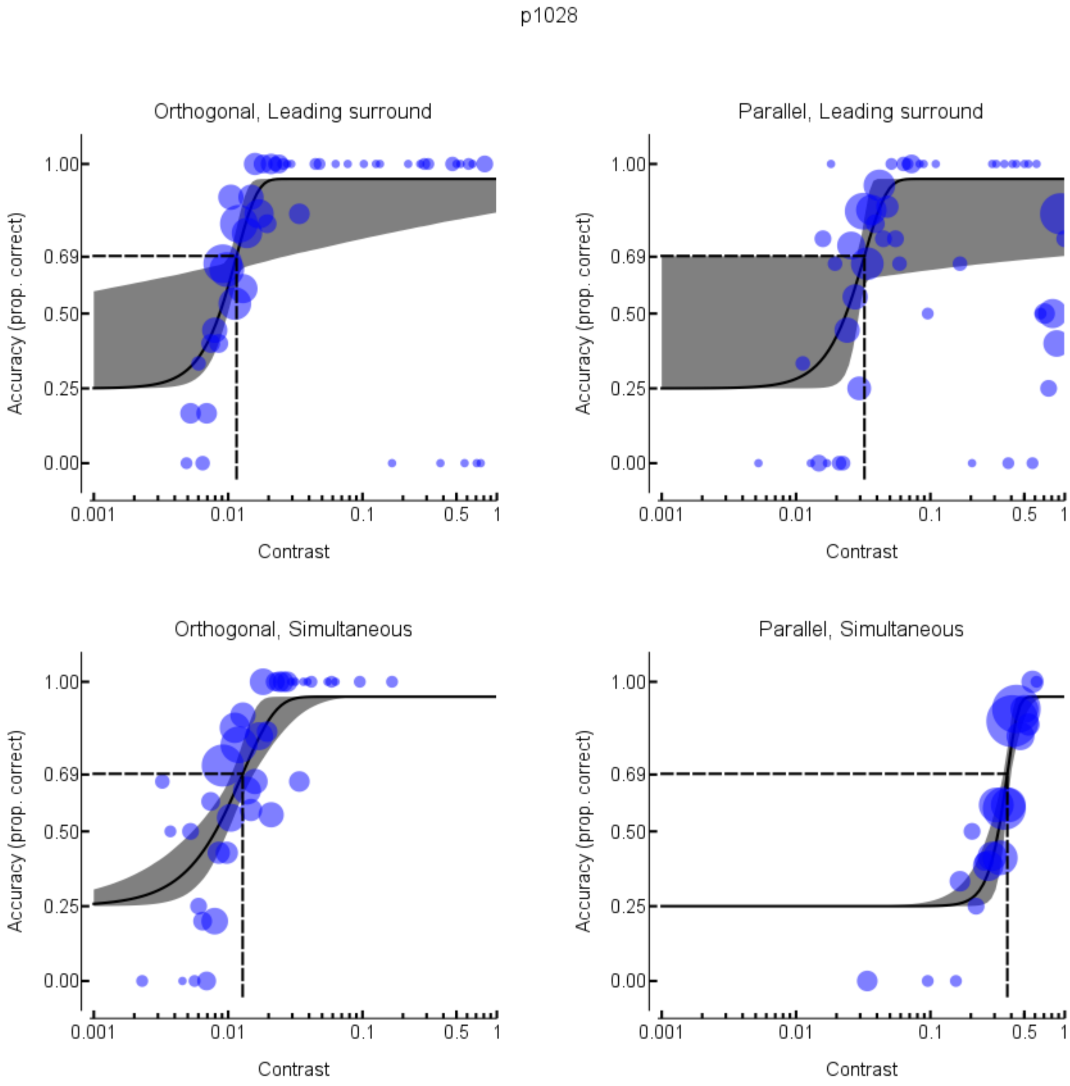
p1028
Orthogonal, Leading surround
Parallel, Leading surround
Orthogonal, Simultaneous
Parallel, Simultaneous
Accuracy (prop. correct)
1.00
0.69
0.50
0.25
0.00
Contrast
0.001
0.01
0.1
0.5
1

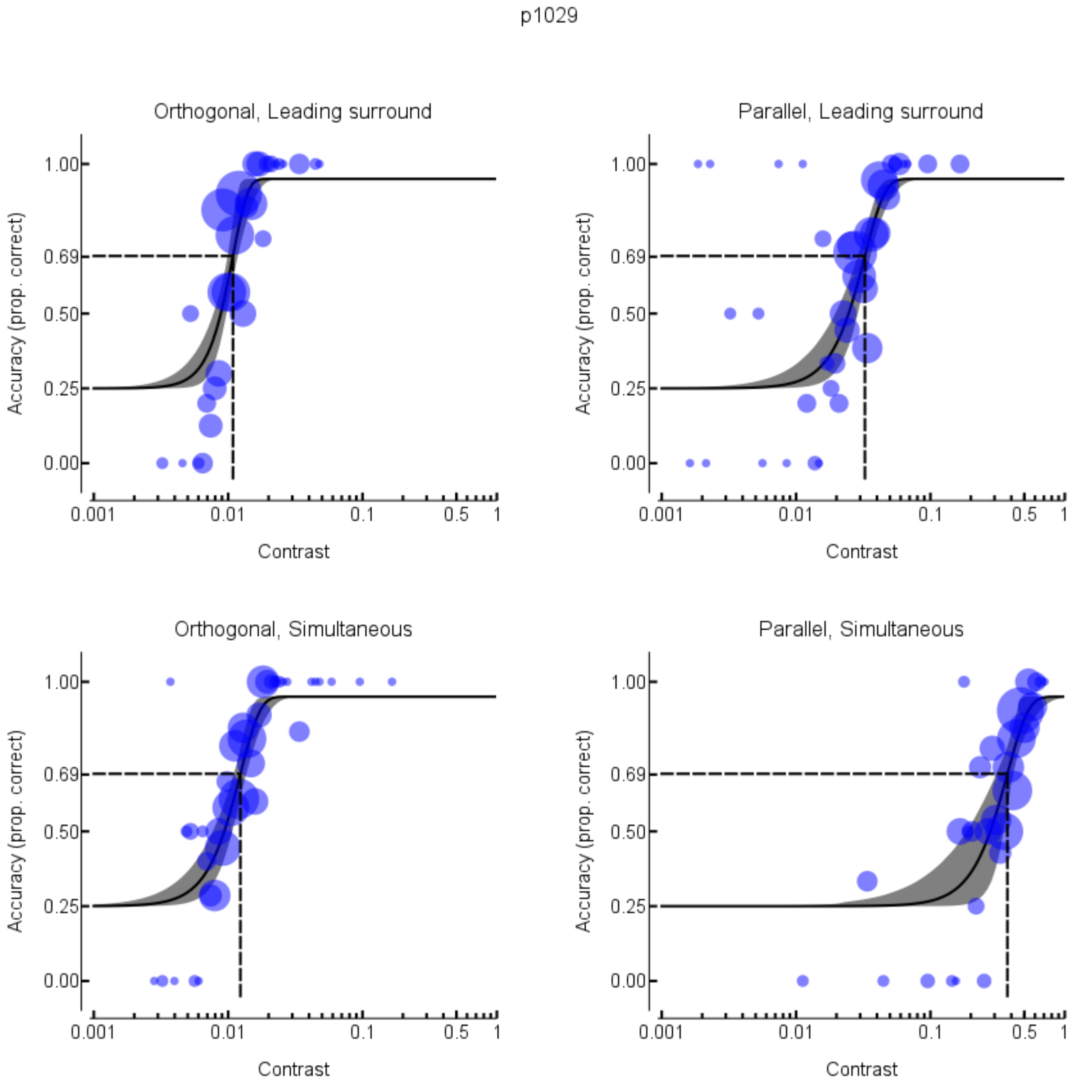
p1029
Orthogonal, Leading surround
Parallel, Leading surround
Orthogonal, Simultaneous
Parallel, Simultaneous
Accuracy (prop. correct)
1.00
0.69
0.50
0.25
0.00
0.001
0.01
0.1
0.5
1
Contrast

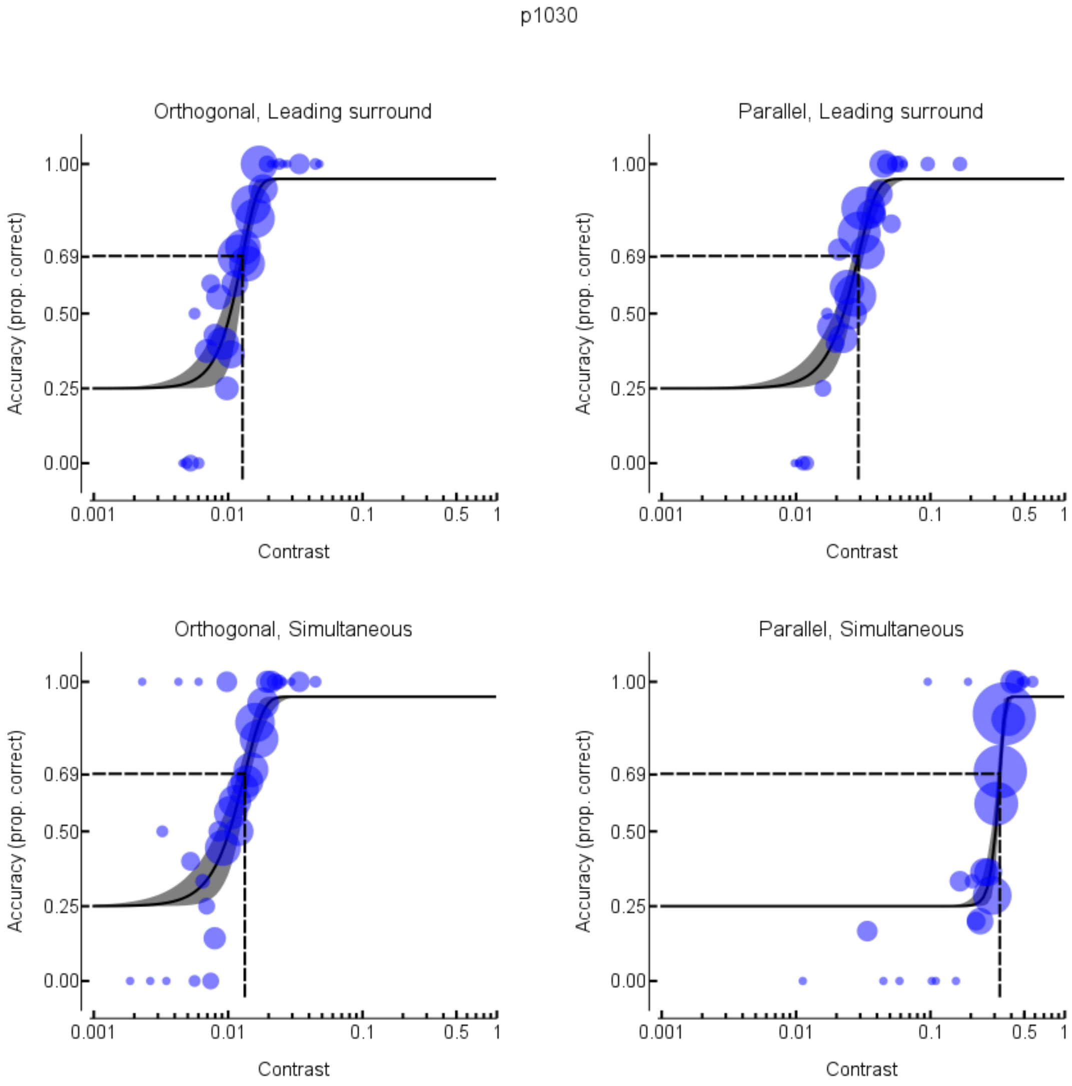
p1030
Orthogonal, Leading surround
Parallel, Leading surround
Orthogonal, Simultaneous
Parallel, Simultaneous
Accuracy (prop. correct)
1.00
0.69
0.50
0.25
0.00
0.001
0.01
0.1
0.5
1
Contrast

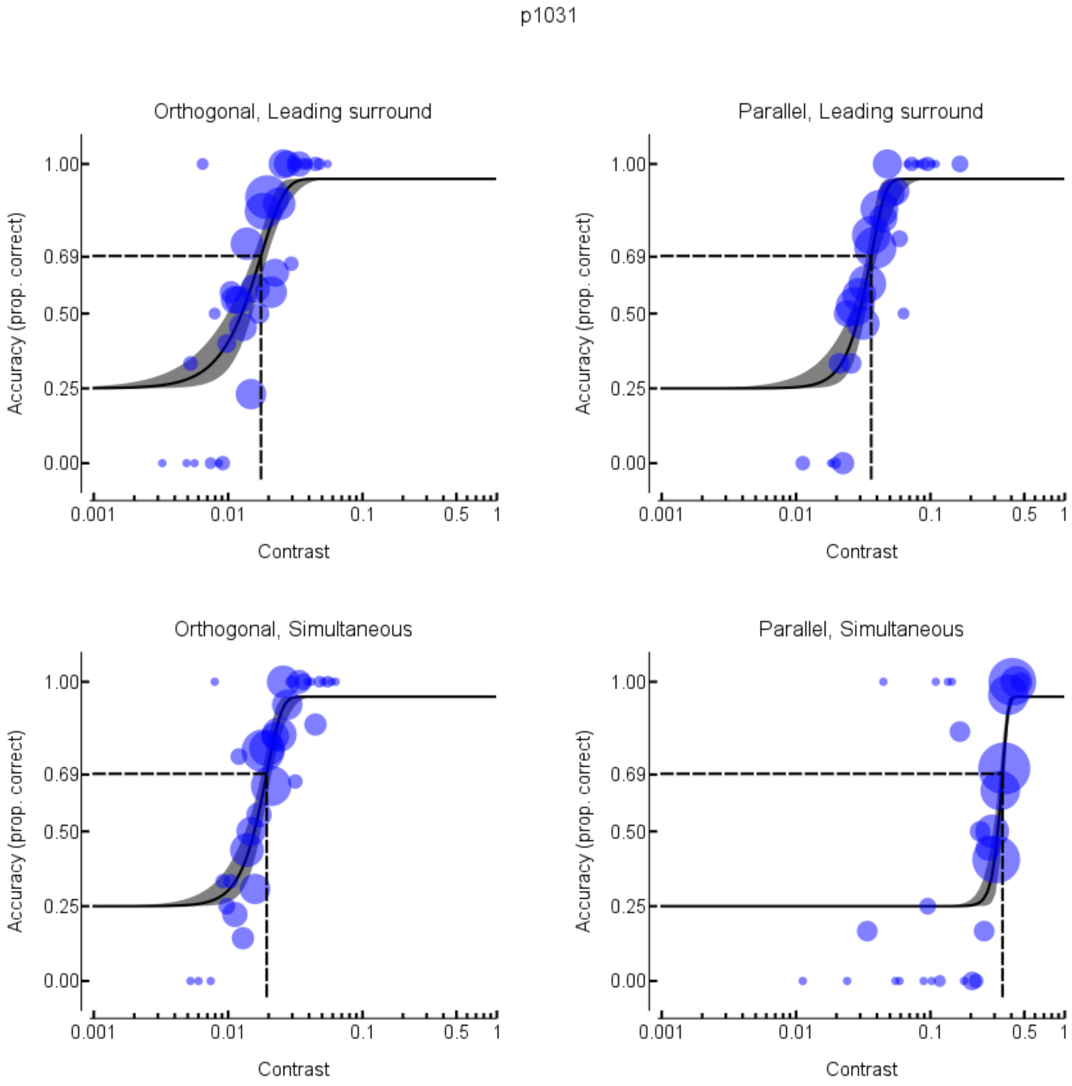
p1031
Orthogonal, Leading surround
Parallel, Leading surround
Orthogonal, Simultaneous
Parallel, Simultaneous
Accuracy (prop. correct)
1.00
0.69
0.50
0.25
0.00
Contrast
0.001
0.01
0.1
0.5
1

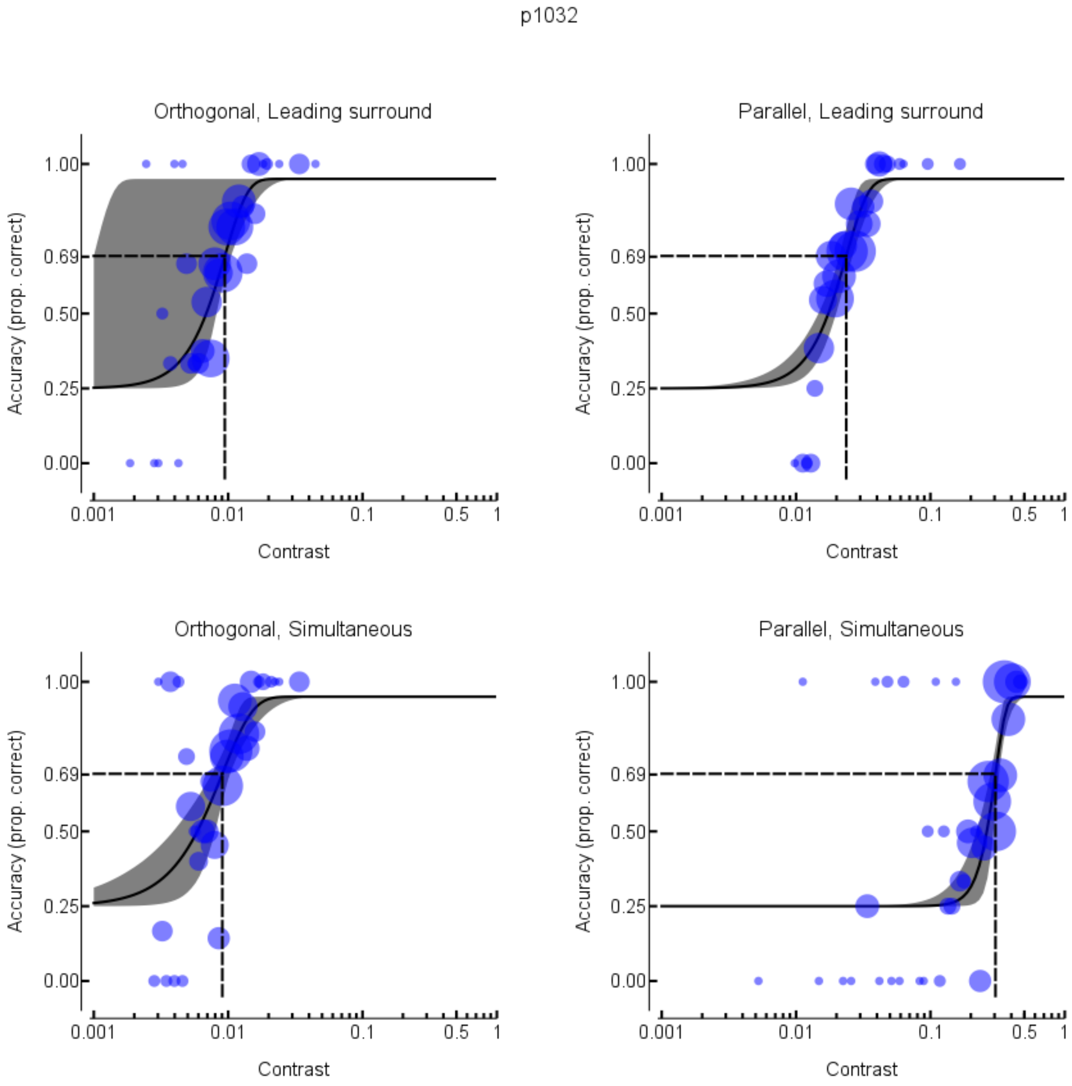
p1032
Orthogonal, Leading surround
Parallel, Leading surround
Orthogonal, Simultaneous
Parallel, Simultaneous
Accuracy (prop. correct)
1.00
0.69
0.50
0.25
0.00
Contrast
0.001
0.01
0.1
0.5
1

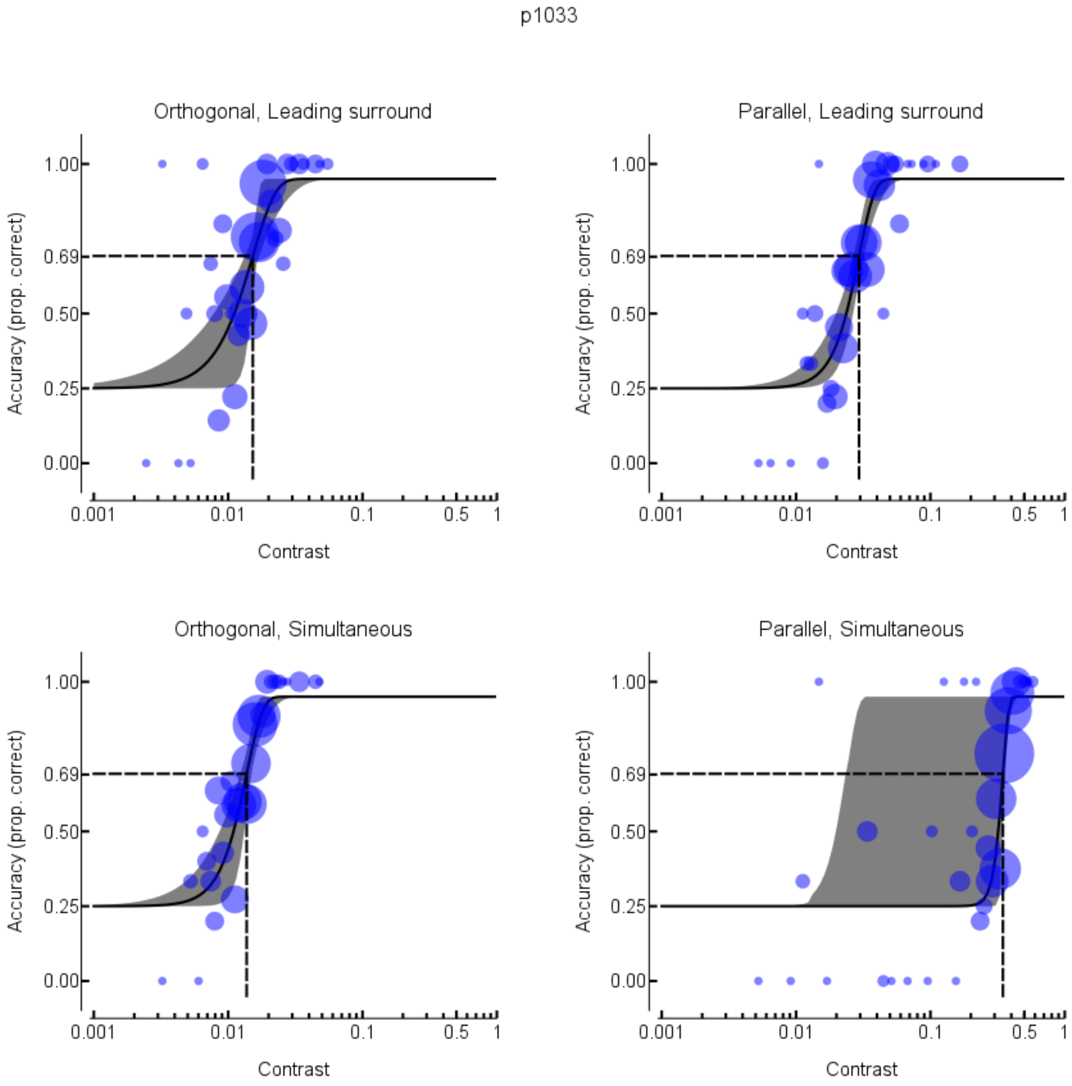
p1033
Orthogonal, Leading surround
Parallel, Leading surround
Orthogonal, Simultaneous
Parallel, Simultaneous
Accuracy (prop. correct)
1.00
0.69
0.50
0.25
0.00
0.001
0.01
0.1
0.5
1
Contrast

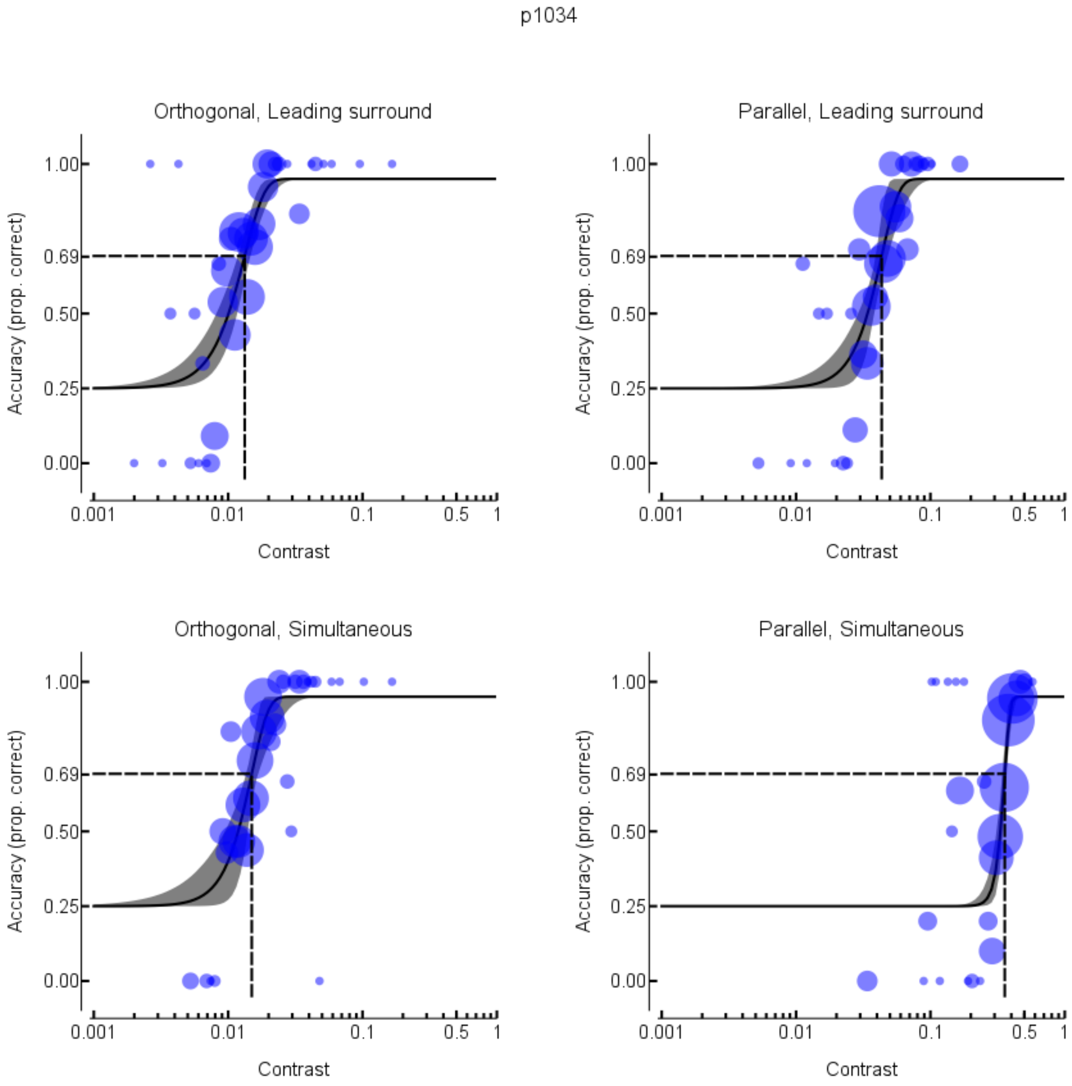
p1034
Orthogonal, Leading surround
Parallel, Leading surround
Orthogonal, Simultaneous
Parallel, Simultaneous
Accuracy (prop. correct)
1.00
0.69
0.50
0.25
0.00
0.001
0.01
0.1
0.5
1
Contrast

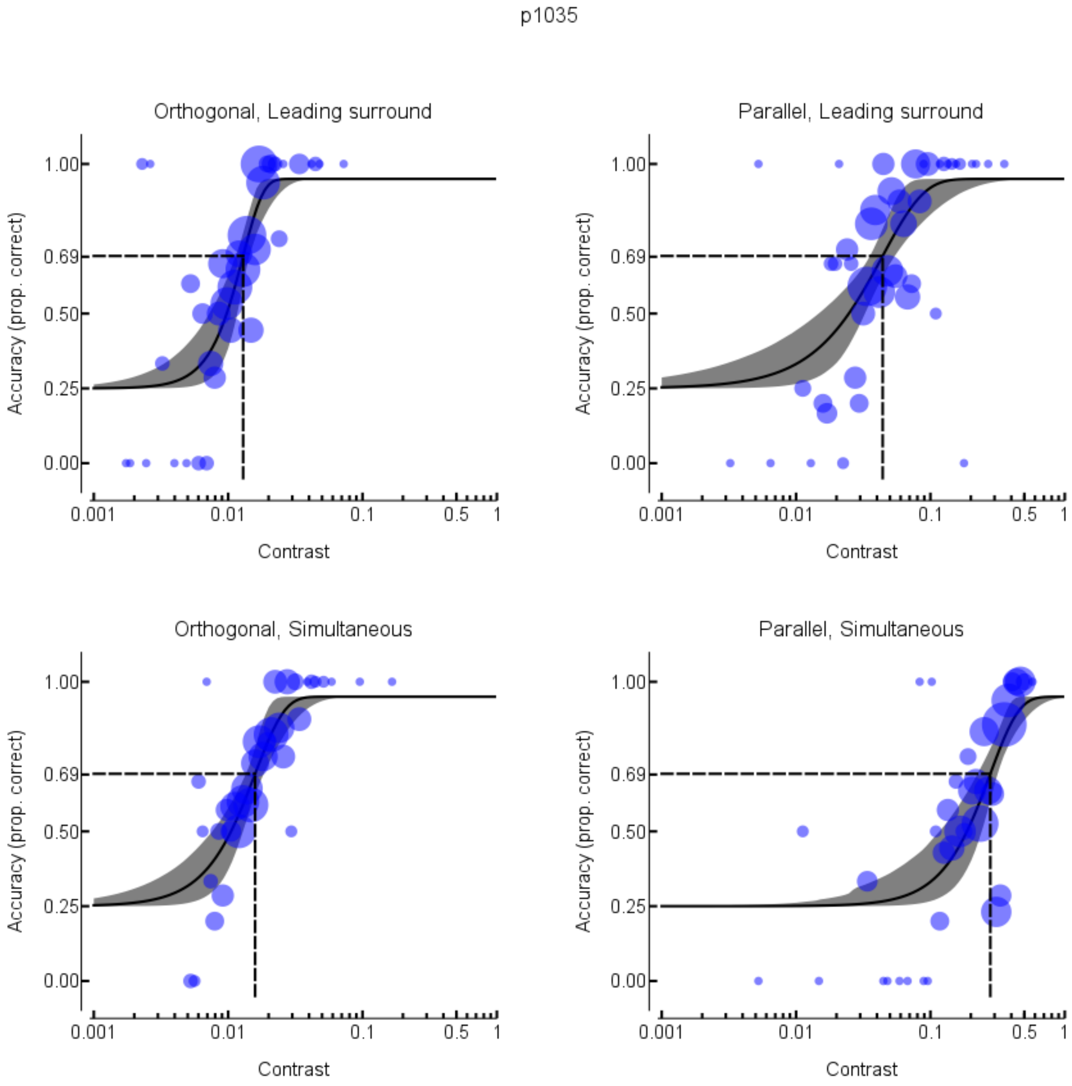
p1035
Orthogonal, Leading surround
Parallel, Leading surround
Orthogonal, Simultaneous
Parallel, Simultaneous
Accuracy (prop. correct)
1.00
0.69
0.50
0.25
0.00
0.001
0.01
0.1
0.5
1
Contrast

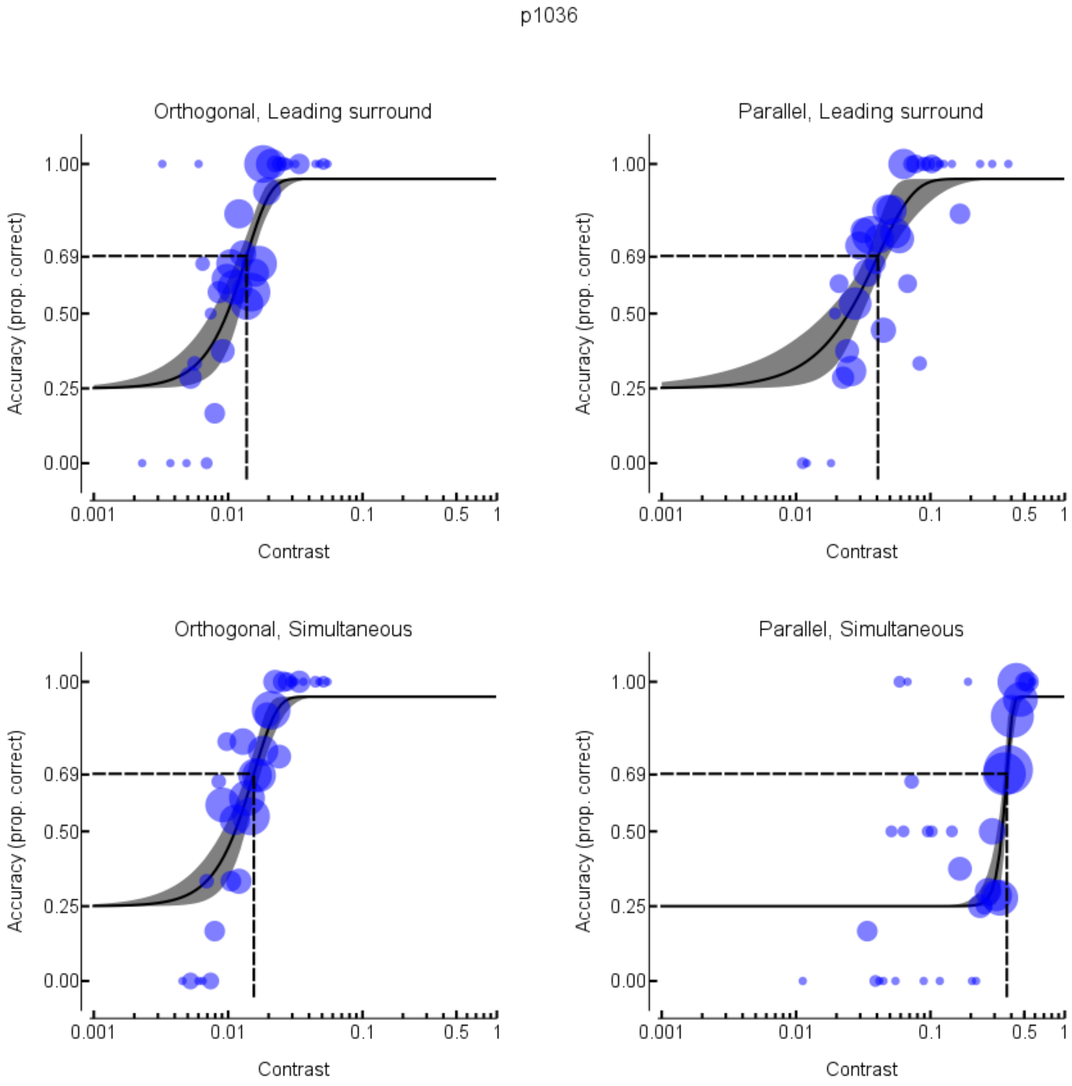
p1036
Orthogonal, Leading surround
Parallel, Leading surround
Orthogonal, Simultaneous
Parallel, Simultaneous
Accuracy (prop. correct)
1.00
0.69
0.50
0.25
0.00
0.001
0.01
0.1
0.5
1
Contrast

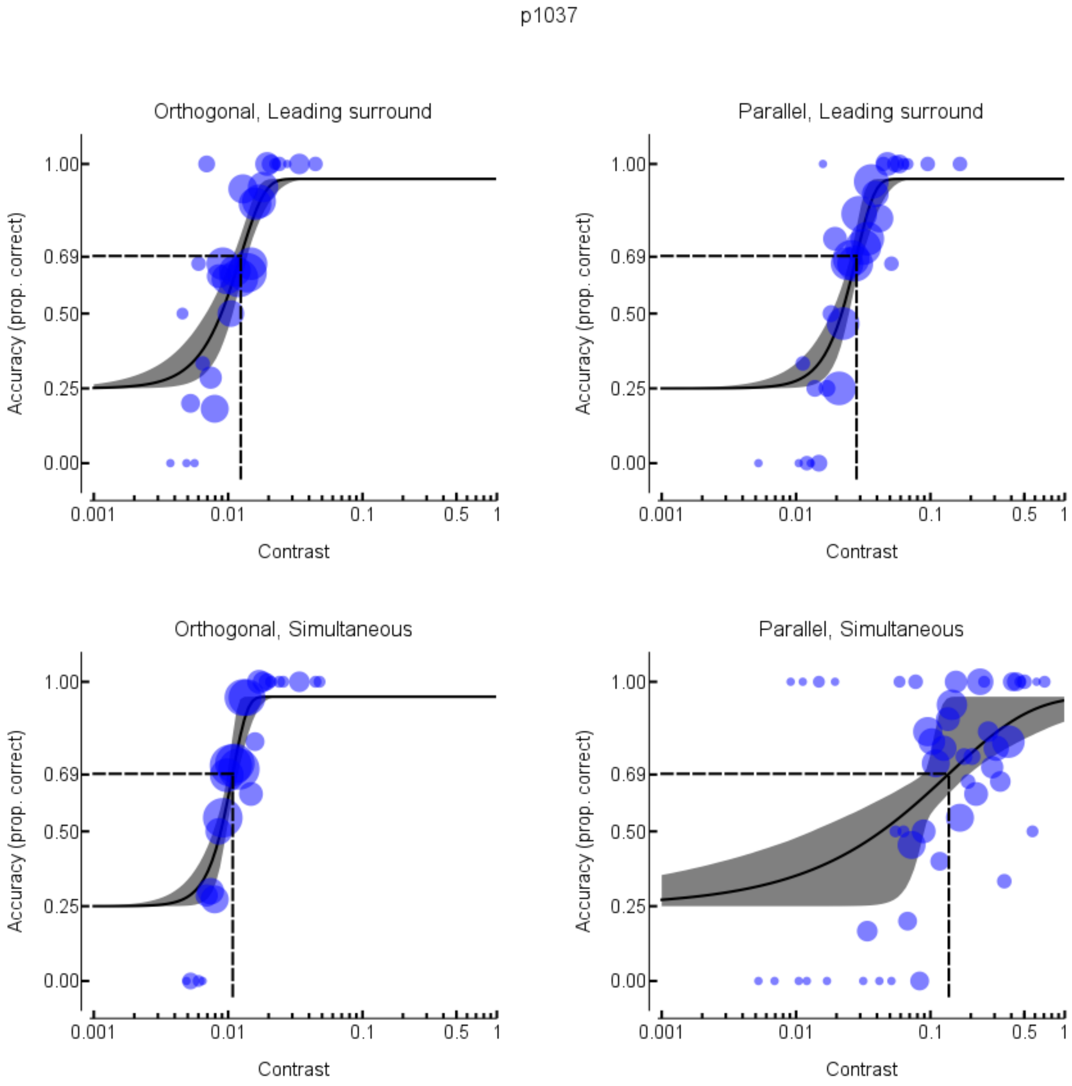
p1037
Orthogonal, Leading surround
Parallel, Leading surround
Orthogonal, Simultaneous
Parallel, Simultaneous
Accuracy (prop. correct)
1.00
0.69
0.50
0.25
0.00
0.001
0.01
0.1
0.5
1
Contrast

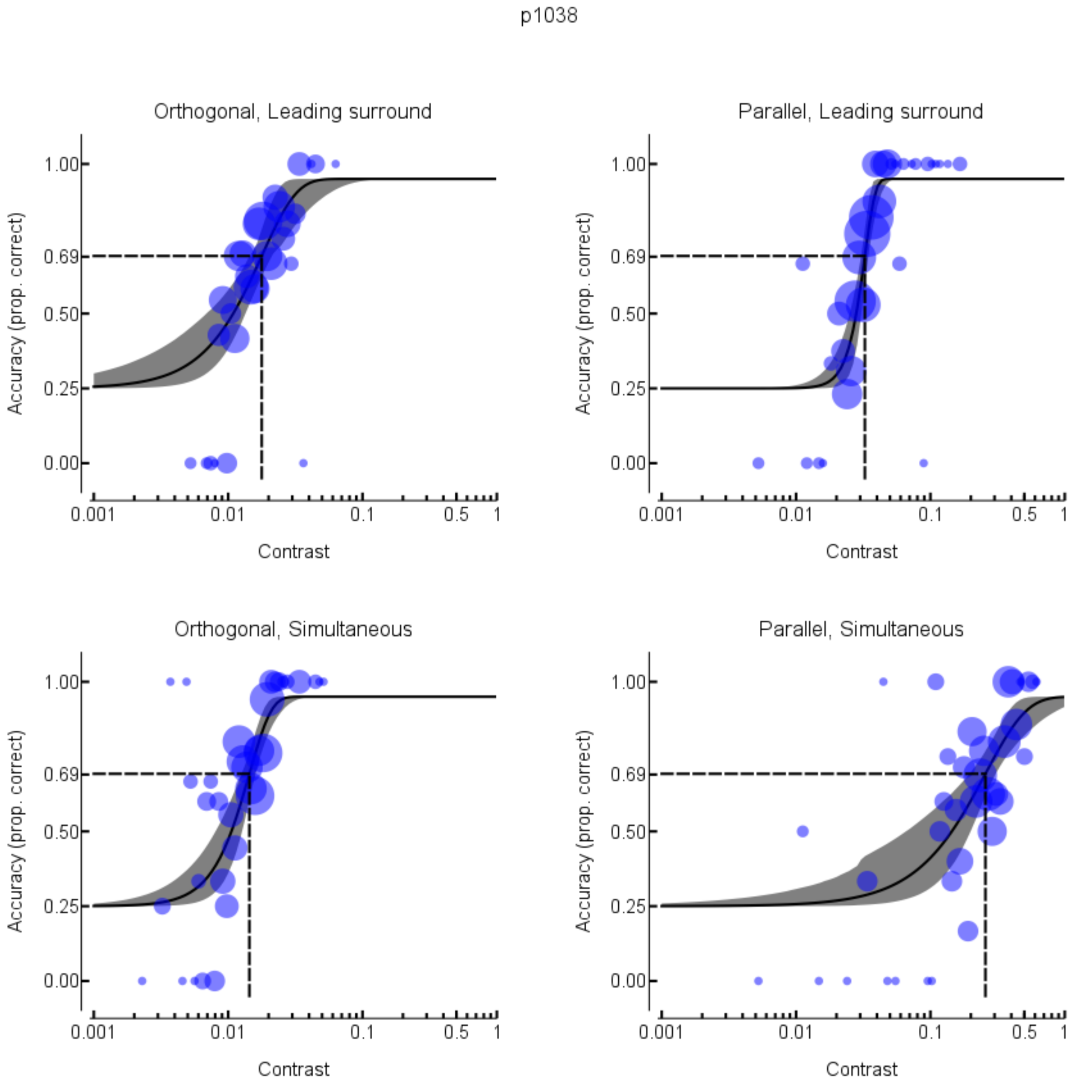
p1038
Orthogonal, Leading surround
Parallel, Leading surround
Orthogonal, Simultaneous
Parallel, Simultaneous
Accuracy (prop. correct)
1.00
0.69
0.50
0.25
0.00
Contrast
0.001
0.01
0.1
0.5
1

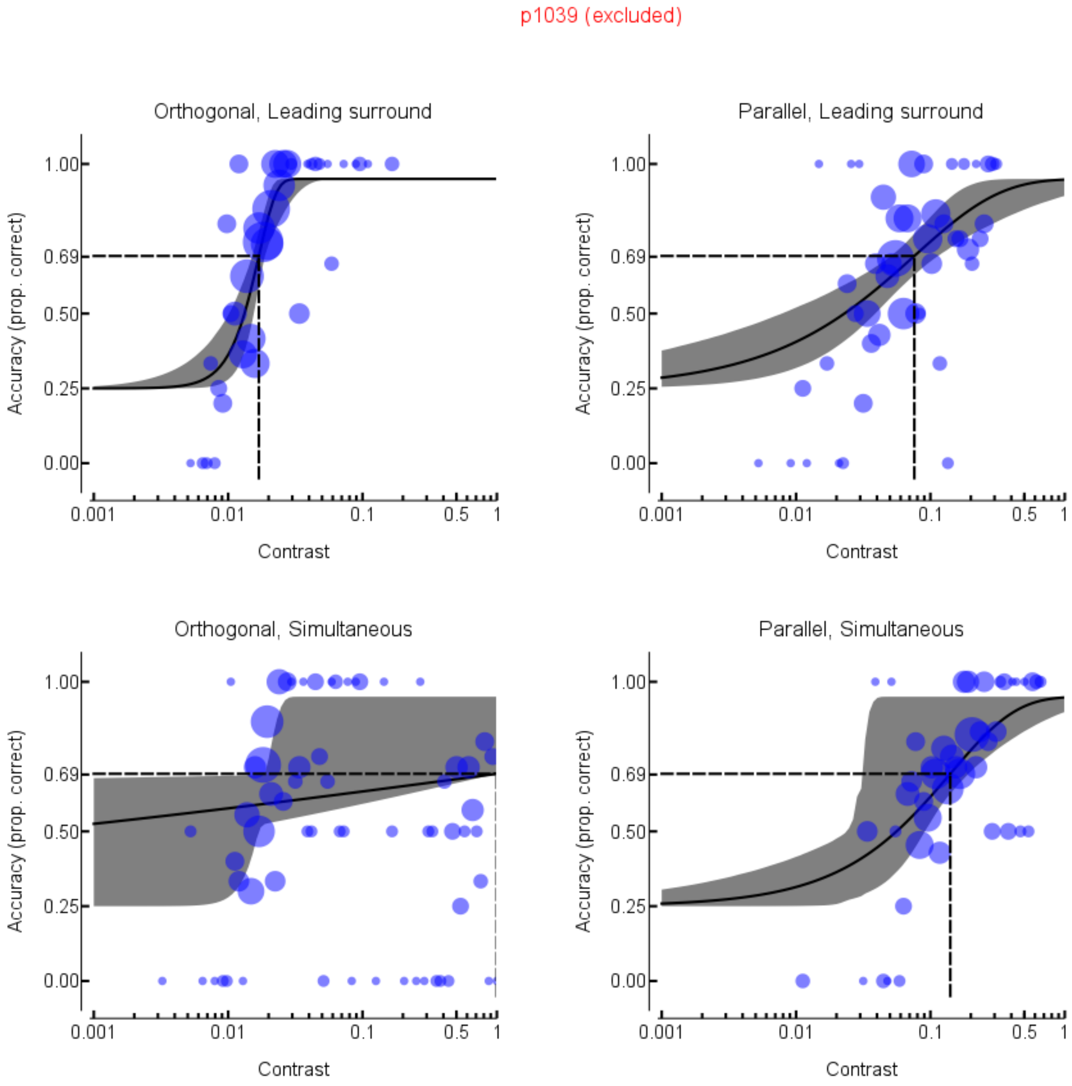
p1039 (excluded)
Orthogonal, Leading surround
Parallel, Leading surround
Orthogonal, Simultaneous
Parallel, Simultaneous
Accuracy (prop. correct)
1.00
0.69
0.50
0.25
0.00
0.001
0.01
0.1
0.5
1
Contrast

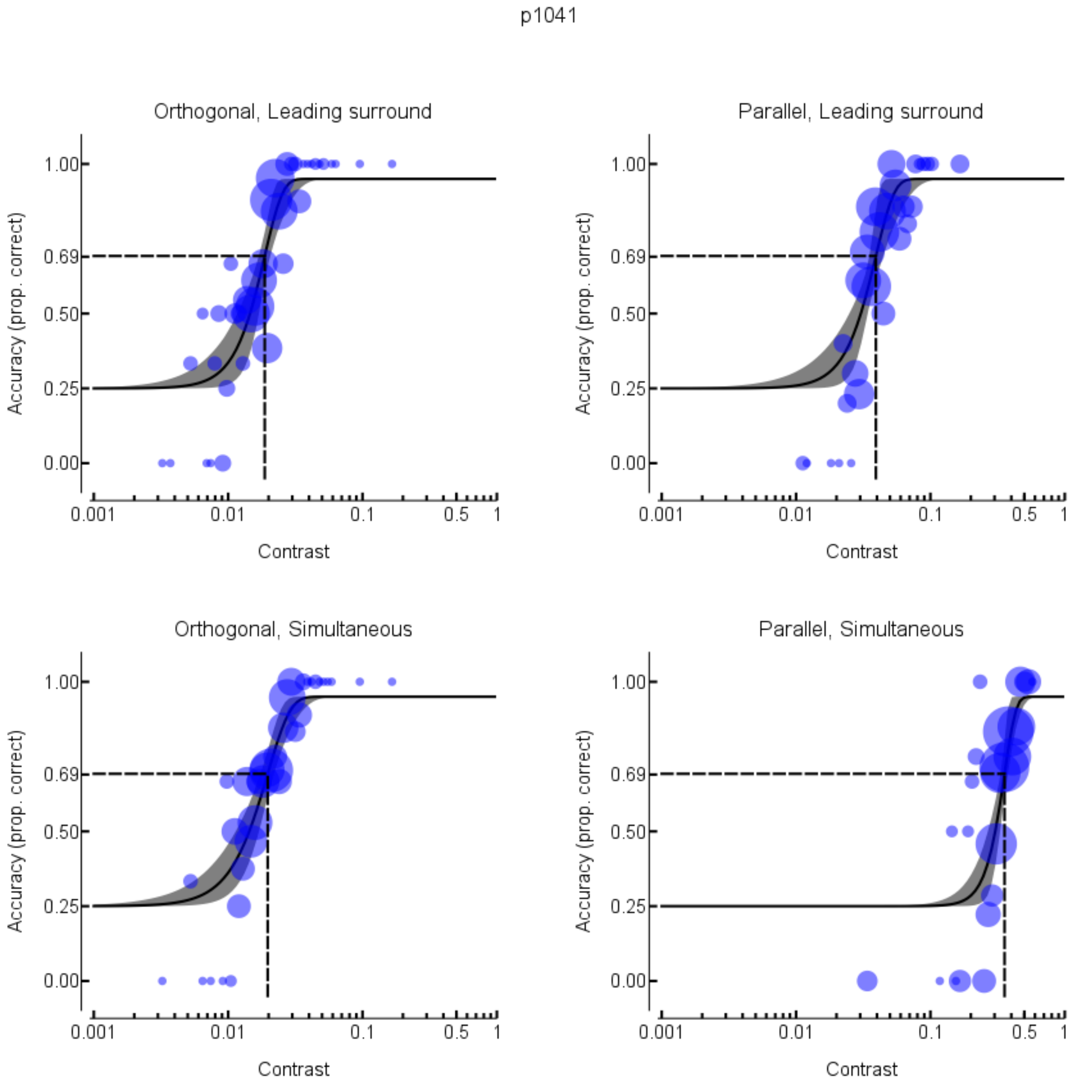
p1041
Orthogonal, Leading surround
Parallel, Leading surround
Orthogonal, Simultaneous
Parallel, Simultaneous
Accuracy (prop. correct)
1.00
0.69
0.50
0.25
0.00
0.001
0.01
0.1
0.5
1
Contrast

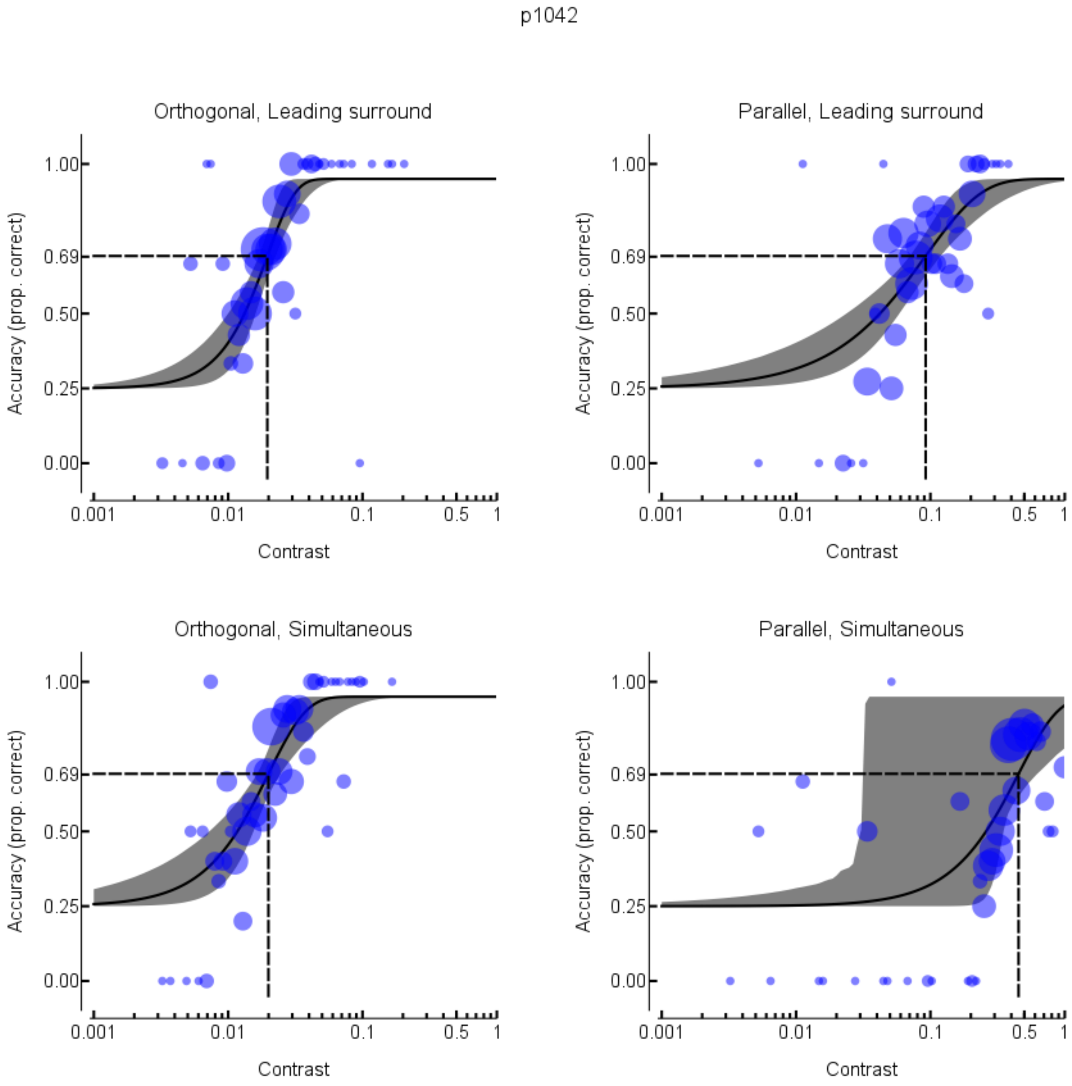
p1042
Orthogonal, Leading surround
Parallel, Leading surround
Orthogonal, Simultaneous
Parallel, Simultaneous
Accuracy (prop. correct)
1.00
0.69
0.50
0.25
0.00
Contrast
0.001
0.01
0.1
0.5
1

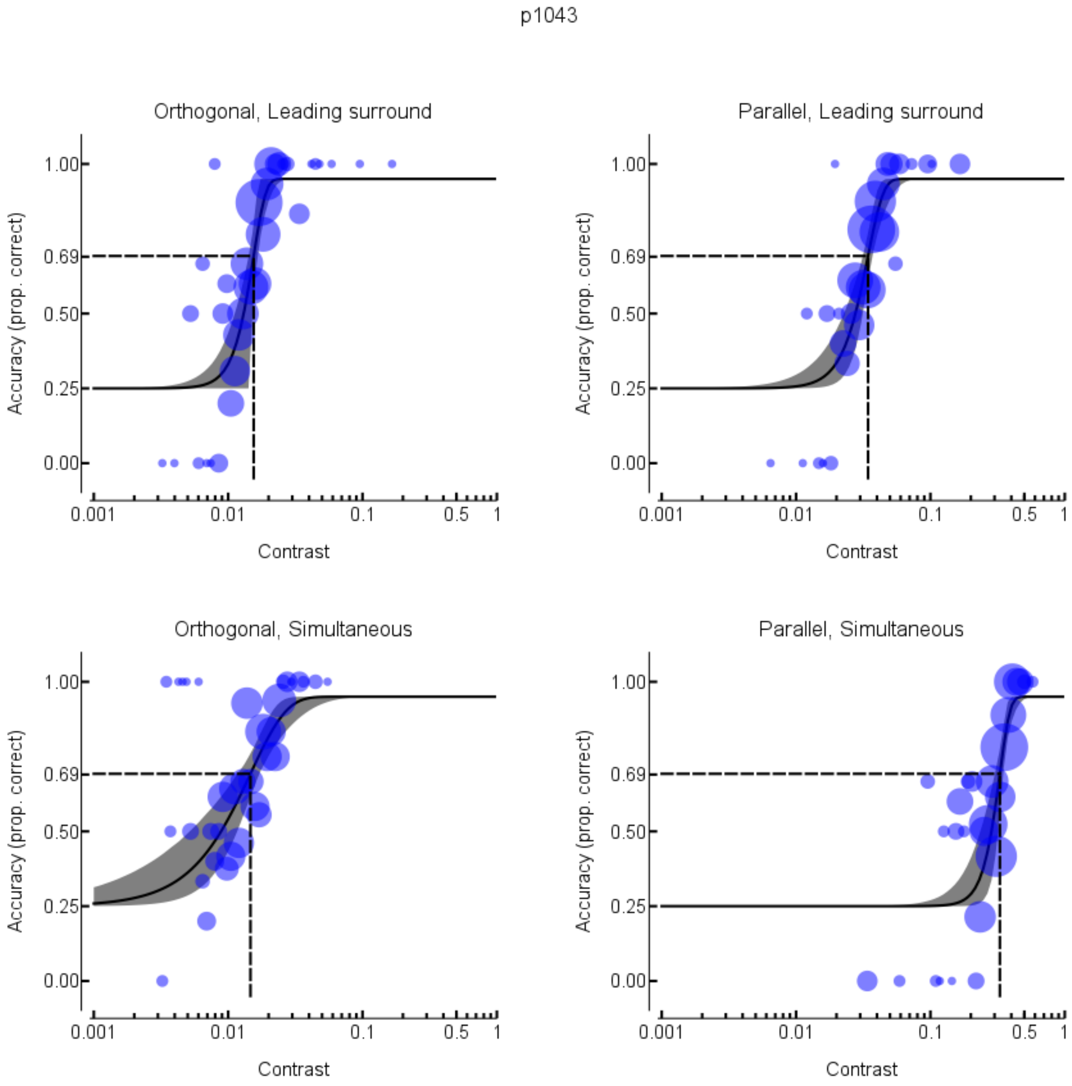
p1043
Orthogonal, Leading surround
Parallel, Leading surround
Orthogonal, Simultaneous
Parallel, Simultaneous
Accuracy (prop. correct)
1.00
0.69
0.50
0.25
0.00
0.001
0.01
0.1
0.5
1
Contrast

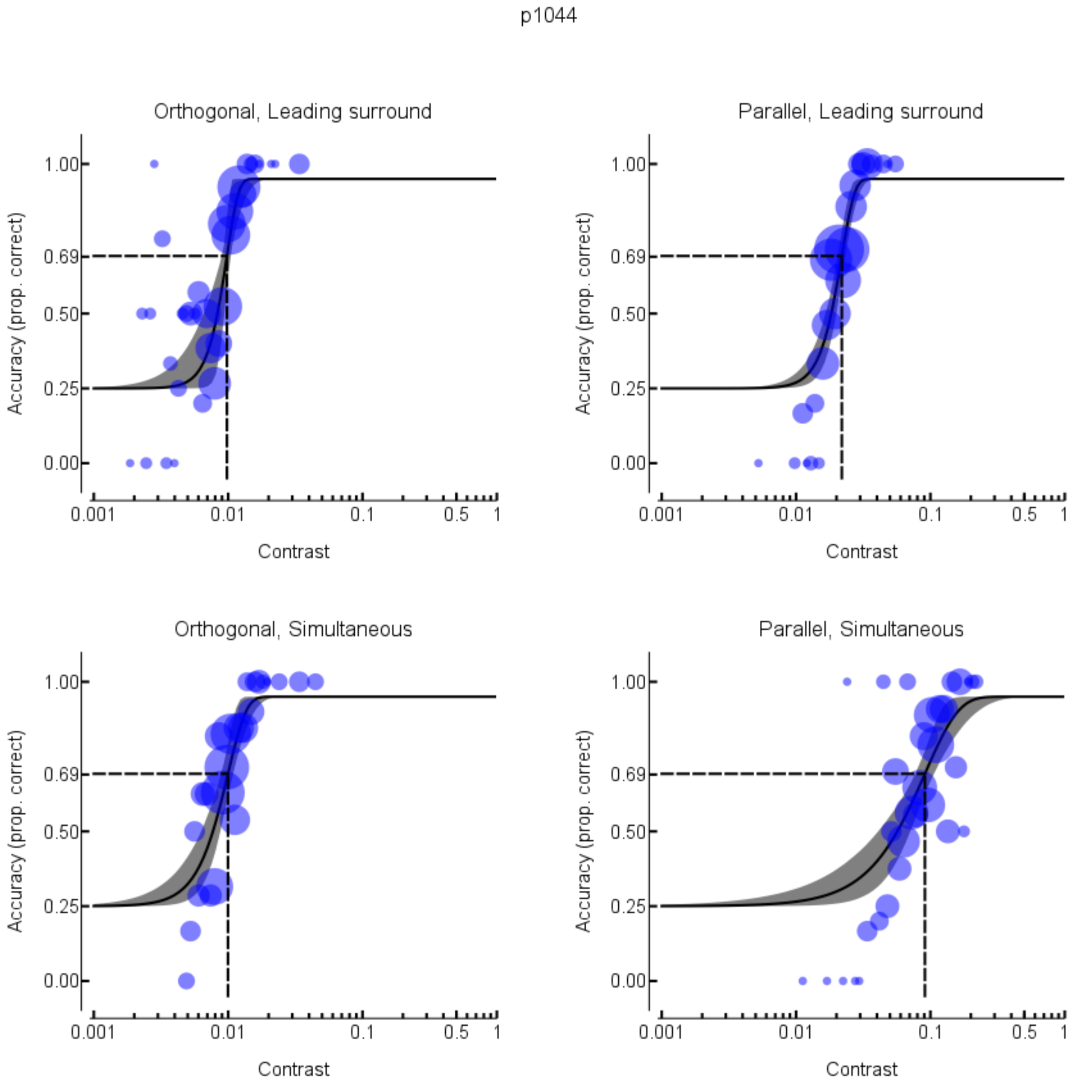
p1044
Orthogonal, Leading surround
Parallel, Leading surround
Orthogonal, Simultaneous
Parallel, Simultaneous
Accuracy (prop. correct)
1.00
0.69
0.50
0.25
0.00
0.001
0.01
0.1
0.5
1
Contrast

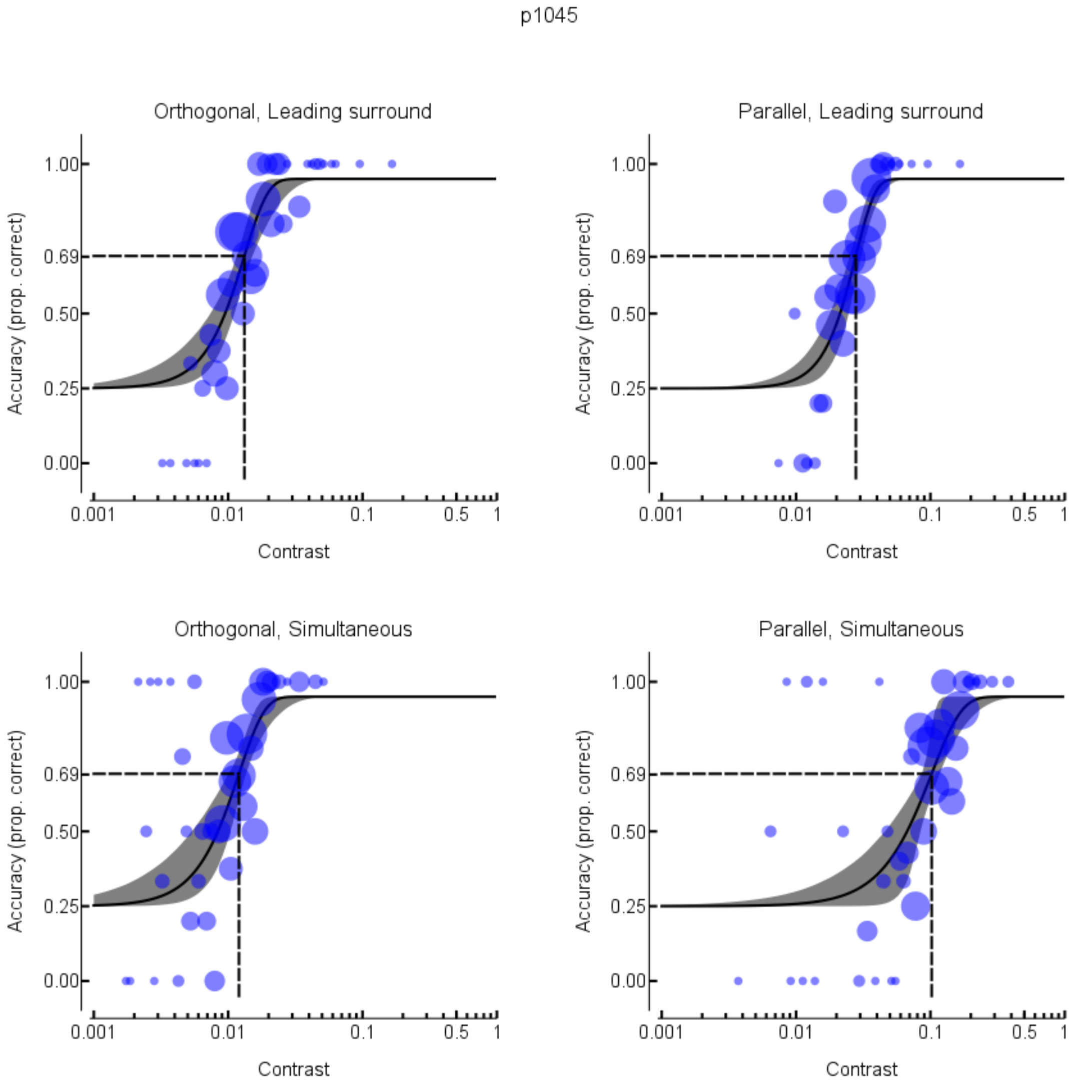
p1045
Orthogonal, Leading surround
Parallel, Leading surround
Orthogonal, Simultaneous
Parallel, Simultaneous
Accuracy (prop. correct)
1.00
0.69
0.50
0.25
0.00
0.001
0.01
0.1
0.5
1
Contrast

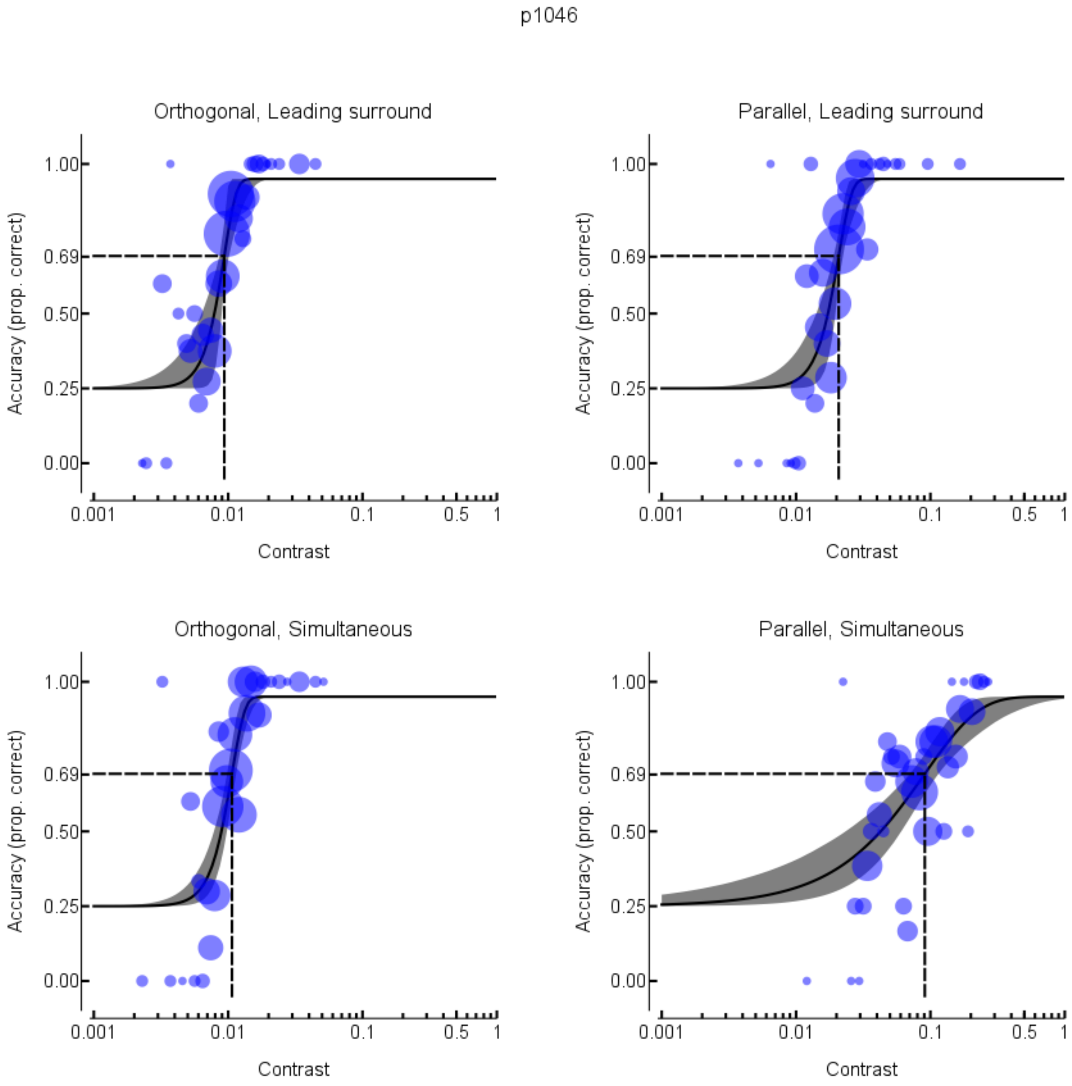
p1046
Orthogonal, Leading surround
Parallel, Leading surround
Orthogonal, Simultaneous
Parallel, Simultaneous
Accuracy (prop. correct)
1.00
0.69
0.50
0.25
0.00
0.001
0.01
0.1
0.5
1
Contrast

p1047

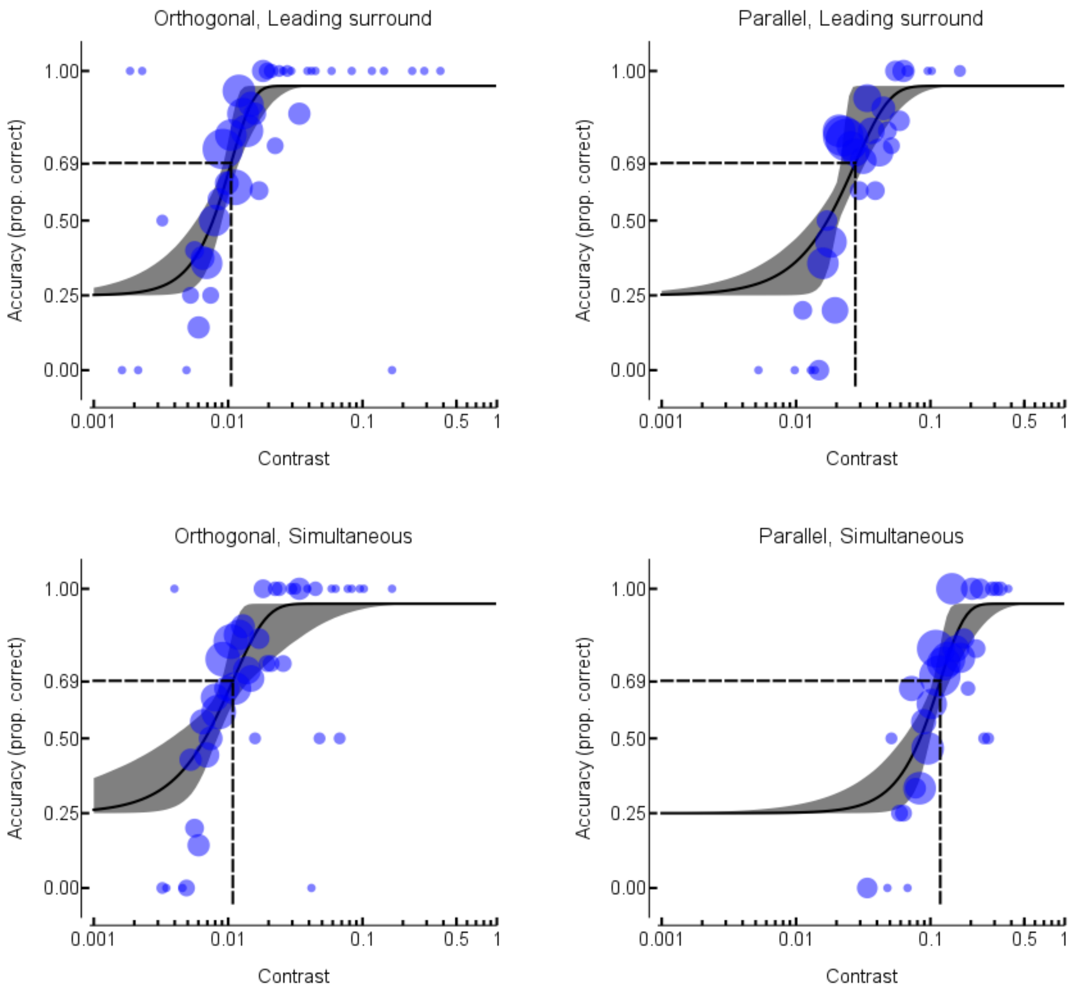
Orthogonal, Leading surround
Parallel, Leading surround
Orthogonal, Simultaneous
Parallel, Simultaneous
Accuracy (prop. correct)
1.00
0.69
0.50
0.25
0.00
0.001
0.01
0.1
0.5
1
Contrast

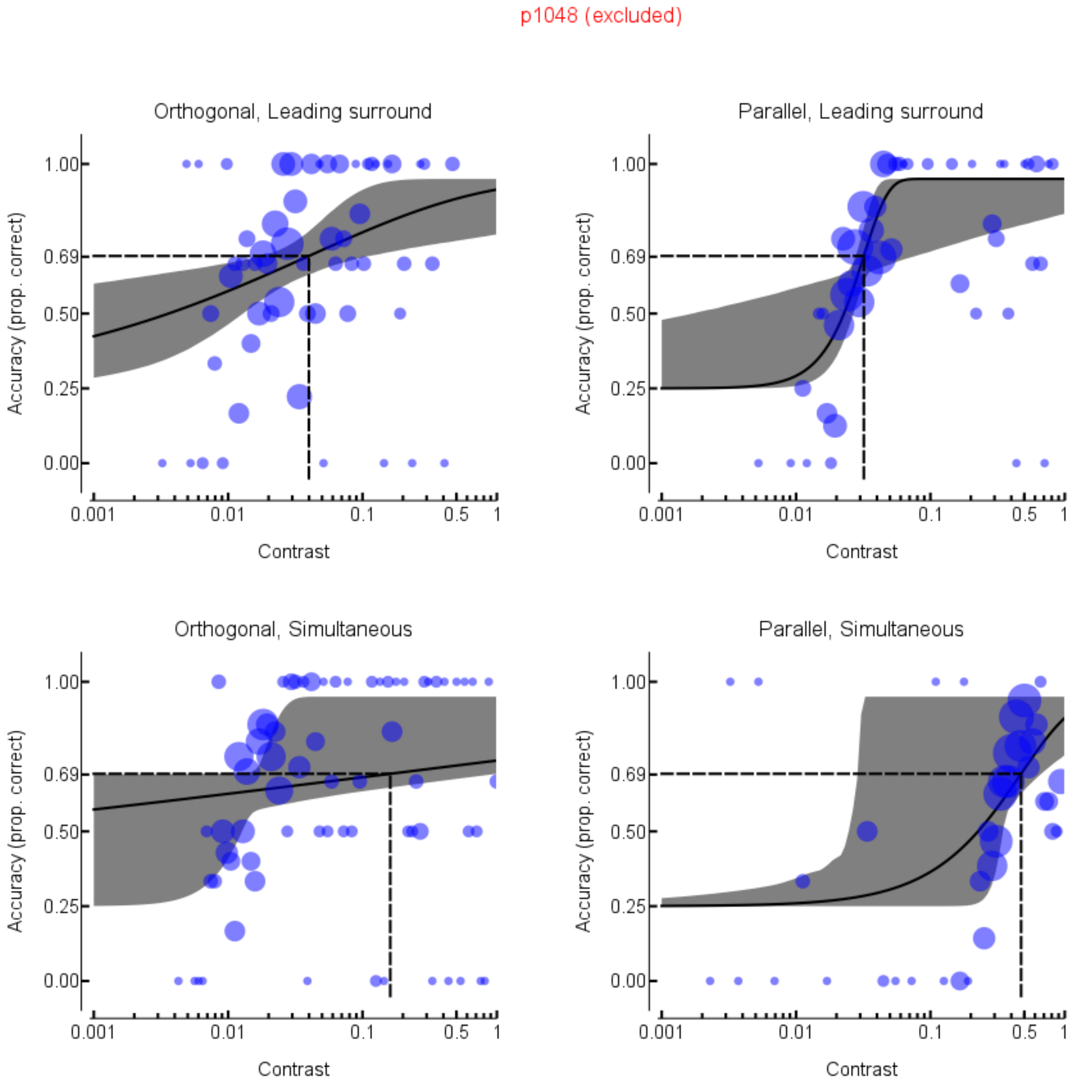
p1048 (excluded)
Orthogonal, Leading surround
Parallel, Leading surround
Orthogonal, Simultaneous
Parallel, Simultaneous
Accuracy (prop. correct)
1.00
0.69
0.50
0.25
0.00
0.001
0.01
0.1
0.5
1
Contrast

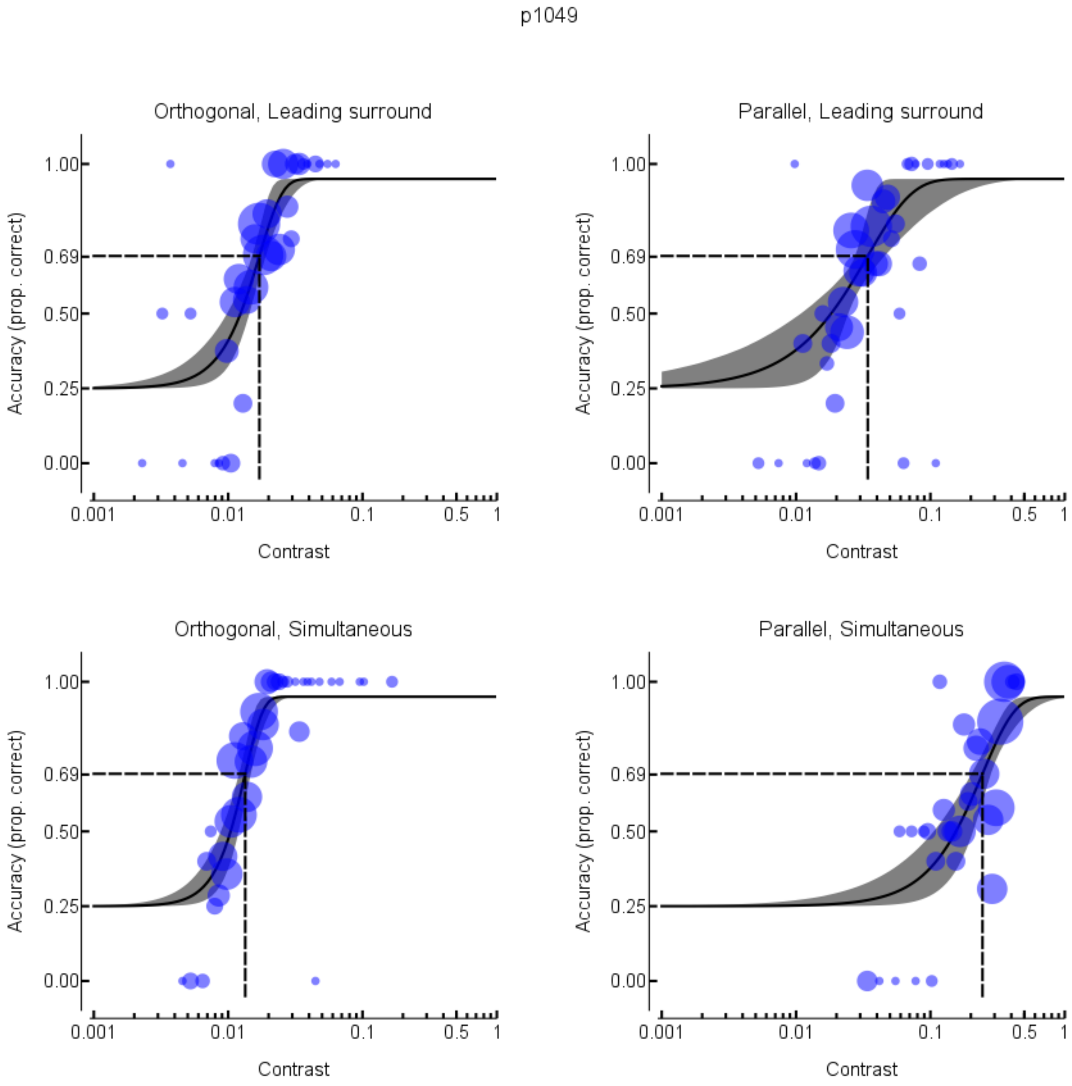
p1049
Orthogonal, Leading surround
Parallel, Leading surround
Orthogonal, Simultaneous
Parallel, Simultaneous
Accuracy (prop. correct)
1.00
0.69
0.50
0.25
0.00
0.001
0.01
0.1
0.5
1
Contrast

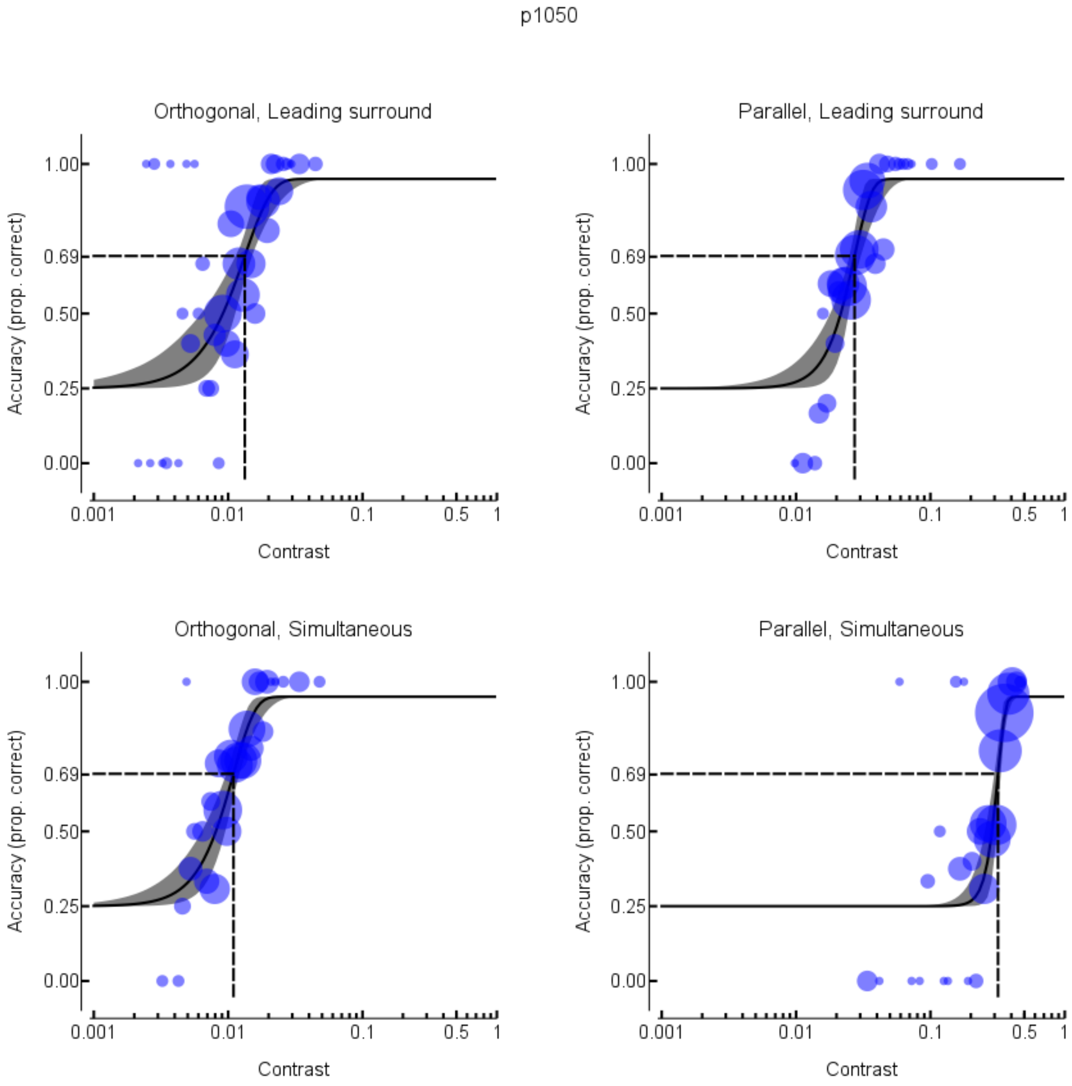
p1050
Orthogonal, Leading surround
Parallel, Leading surround
Orthogonal, Simultaneous
Parallel, Simultaneous
Accuracy (prop. correct)
1.00
0.69
0.50
0.25
0.00
0.001
0.01
0.1
0.5
1
Contrast

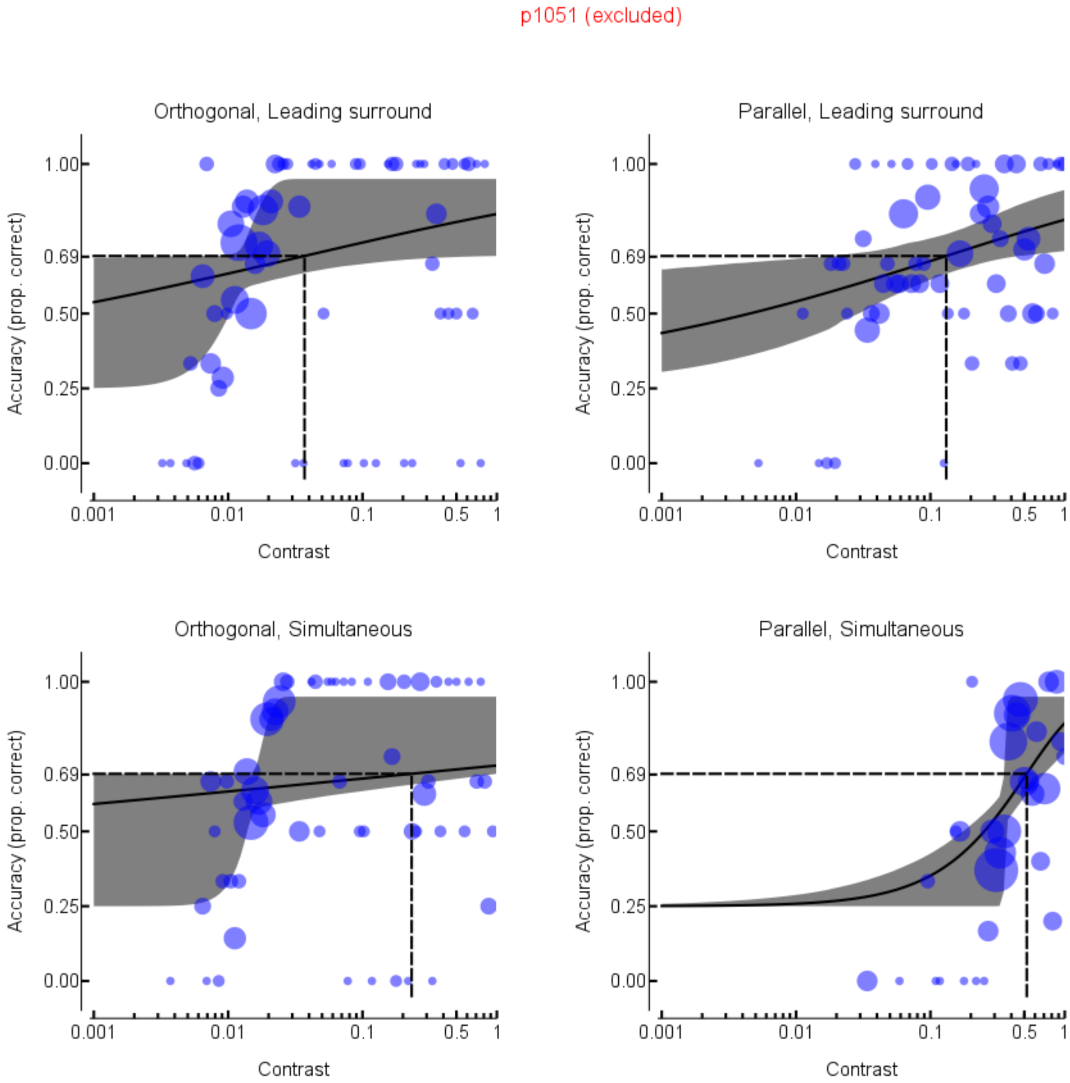
p1051 (excluded)
Orthogonal, Leading surround
Parallel, Leading surround
Orthogonal, Simultaneous
Parallel, Simultaneous
Accuracy (prop. correct)
1.00
0.69
0.50
0.25
0.00
Contrast
0.001
0.01
0.1
0.5
1

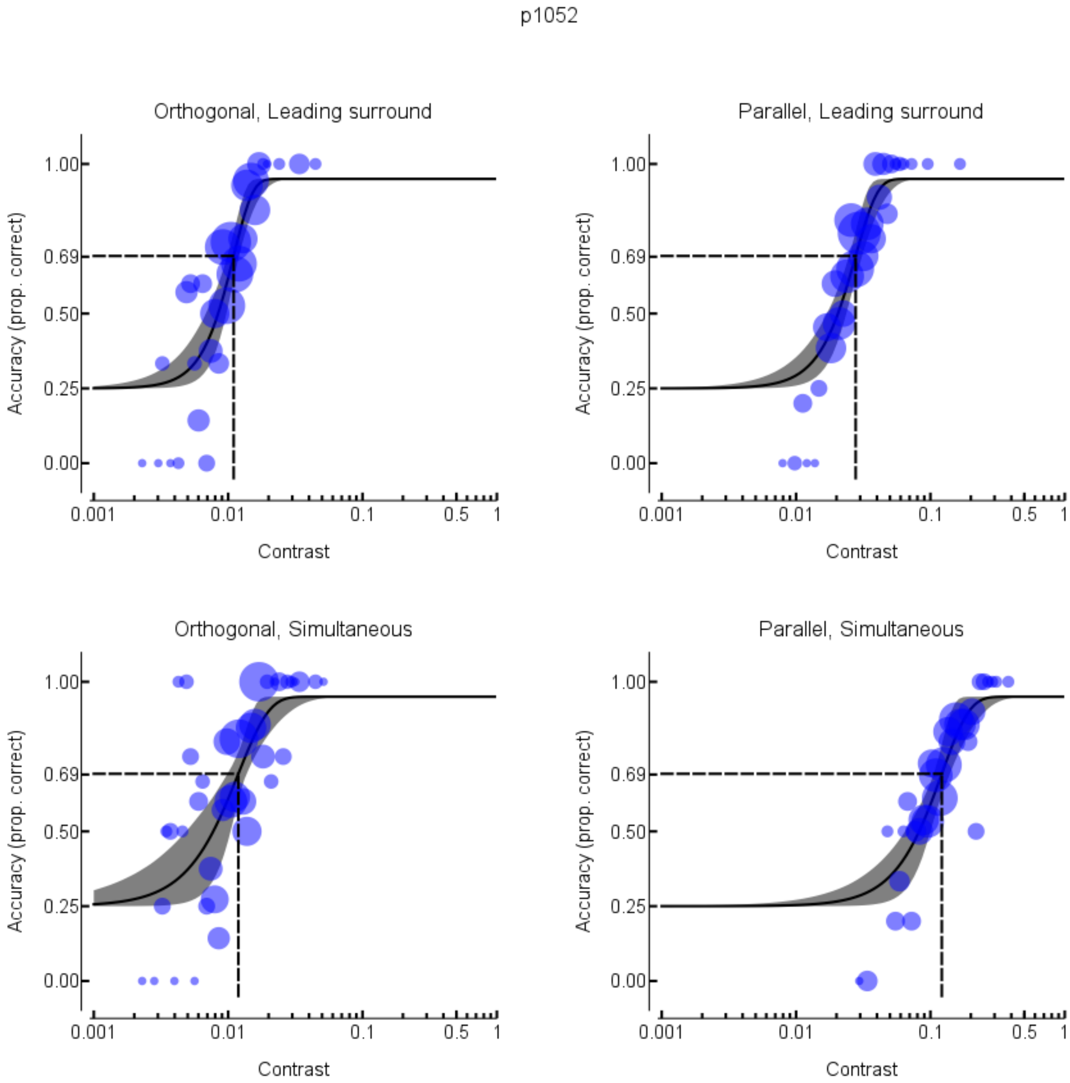
p1052
Orthogonal, Leading surround
Parallel, Leading surround
Orthogonal, Simultaneous
Parallel, Simultaneous
Accuracy (prop. correct)
1.00
0.69
0.50
0.25
0.00
0.001
0.01
0.1
0.5
1
Contrast

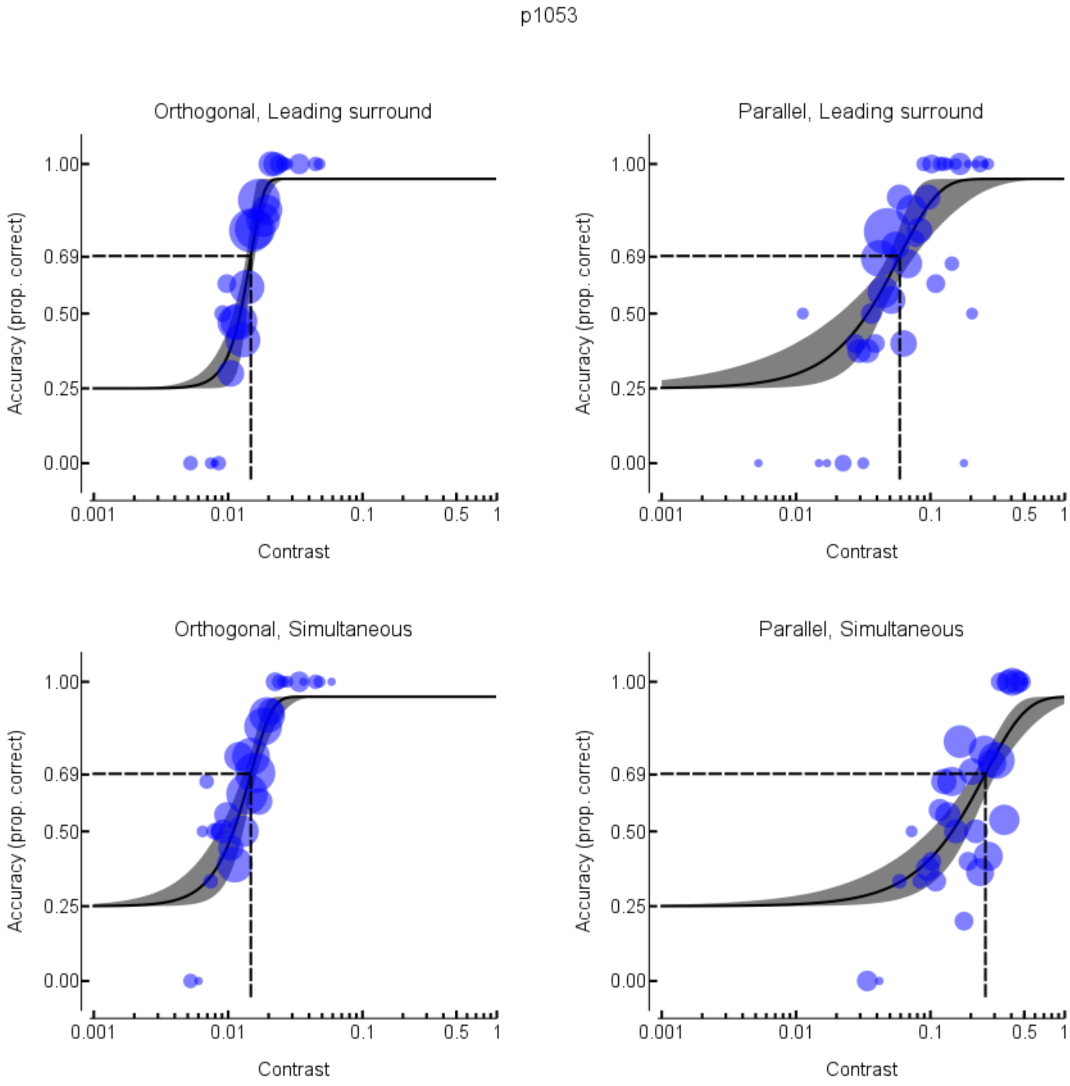
p1053
Orthogonal, Leading surround
Parallel, Leading surround
Orthogonal, Simultaneous
Parallel, Simultaneous
Accuracy (prop. correct)
1.00
0.69
0.50
0.25
0.00
0.001
0.01
0.1
0.5
1
Contrast

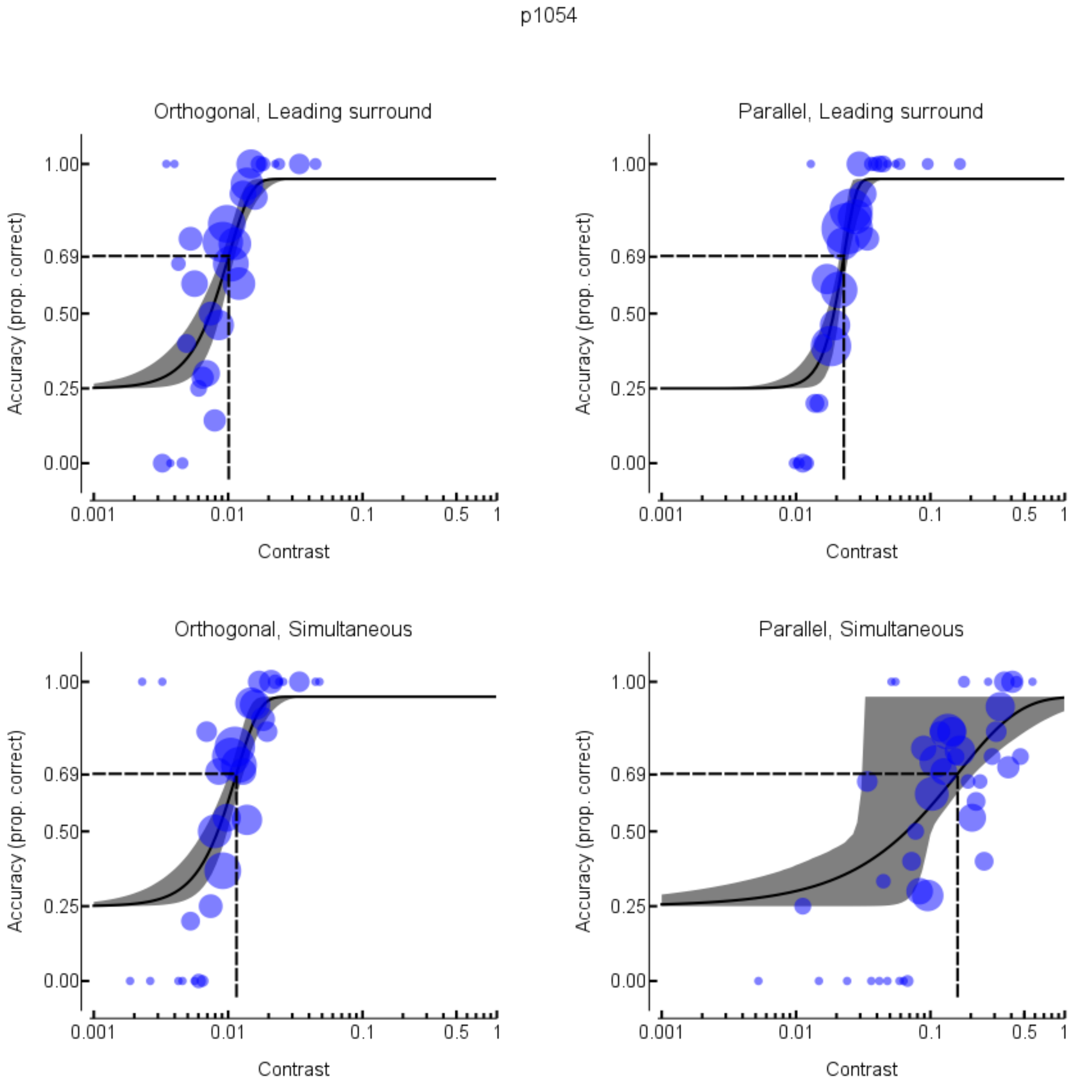

p1054
Orthogonal, Leading surround
Parallel, Leading surround
Orthogonal, Simultaneous
Parallel, Simultaneous
Accuracy (prop. correct)
1.00
0.69
0.50
0.25
0.00
0.001
0.01
0.1
0.5
1
Contrast

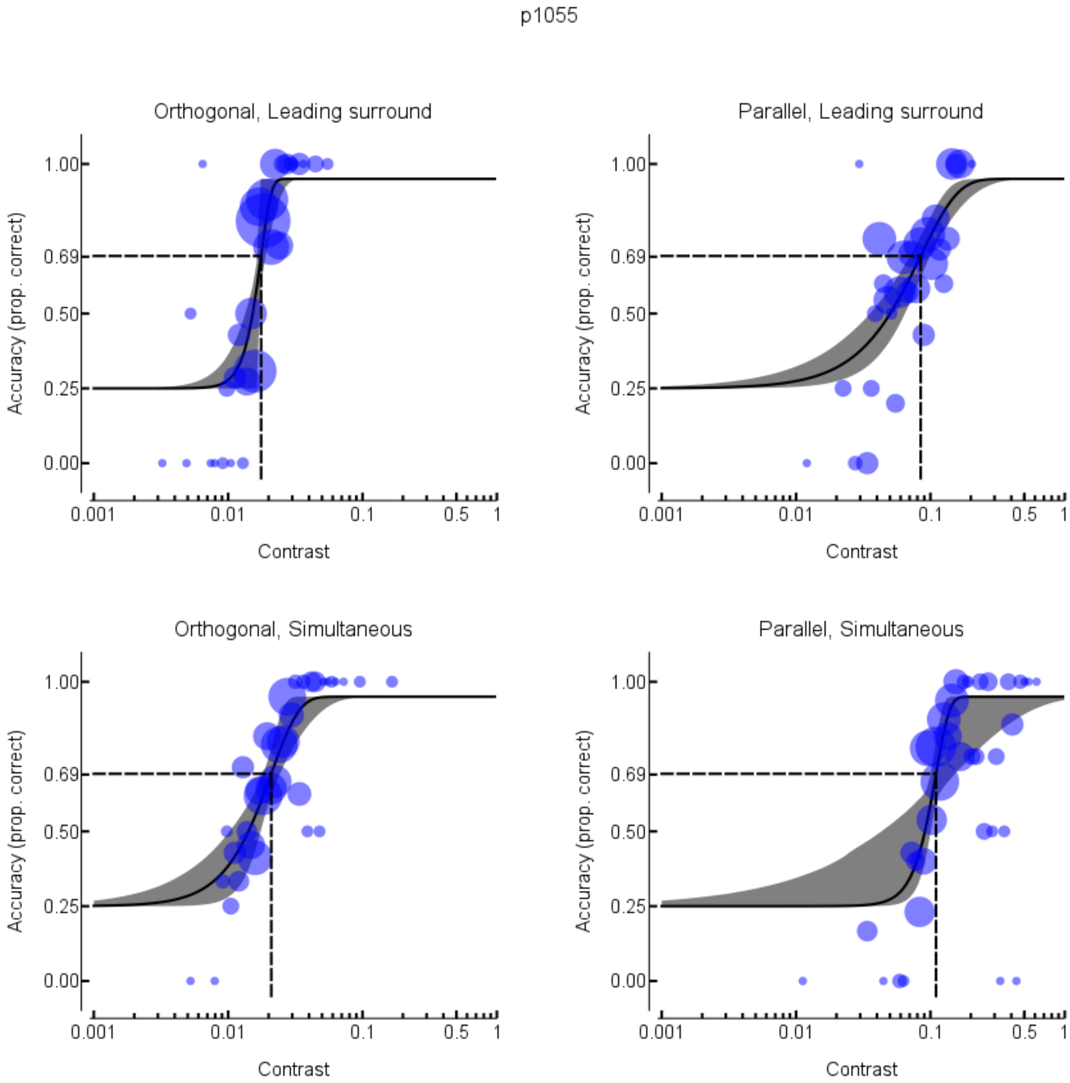
p1055
Orthogonal, Leading surround
Parallel, Leading surround
Orthogonal, Simultaneous
Parallel, Simultaneous
Accuracy (prop. correct)
1.00
0.69
0.50
0.25
0.00
Contrast
0.001
0.01
0.1
0.5
1

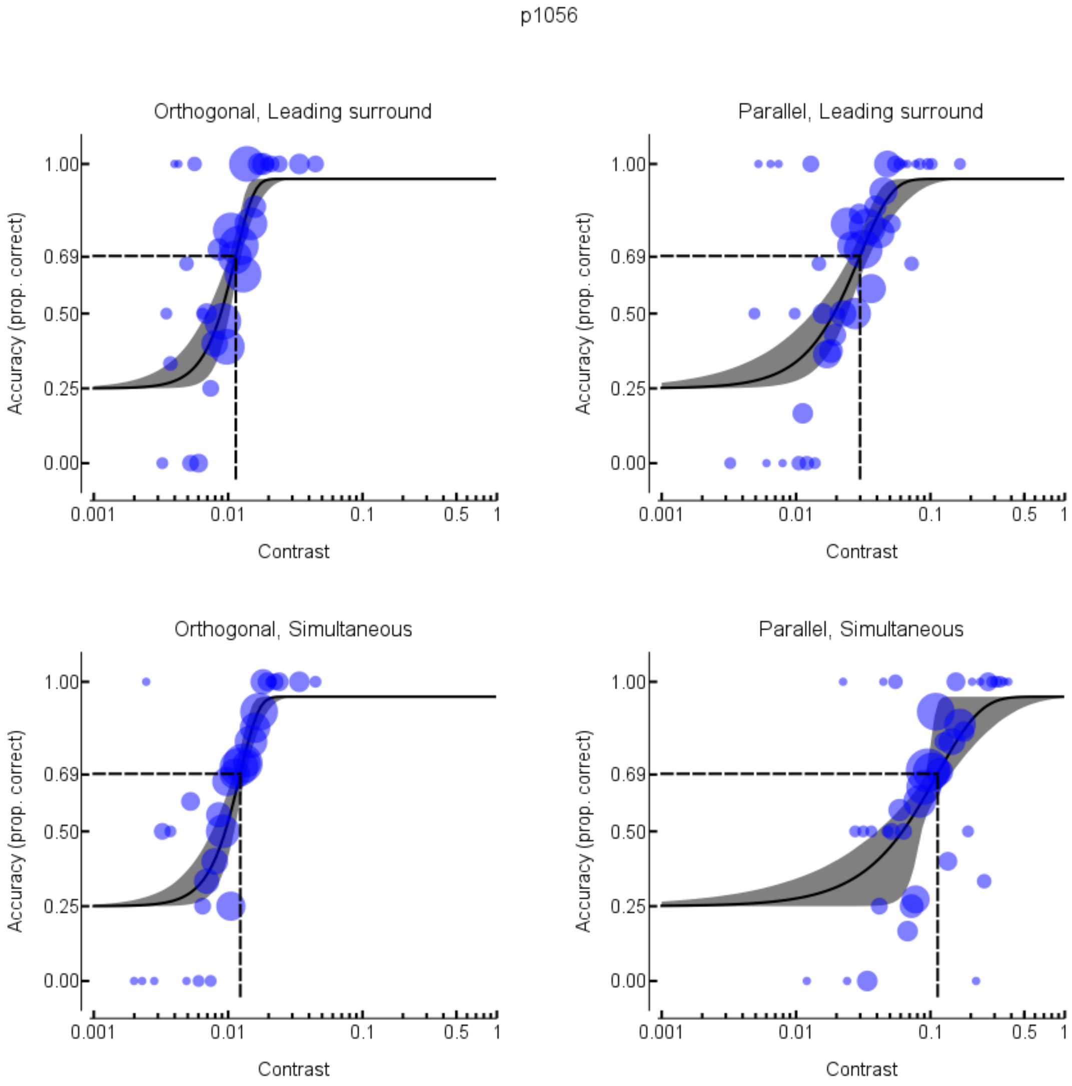
p1056
Orthogonal, Leading surround
Parallel, Leading surround
Orthogonal, Simultaneous
Parallel, Simultaneous
Accuracy (prop. correct)
1.00
0.69
0.50
0.25
0.00
0.001
0.01
0.1
0.5
1
Contrast

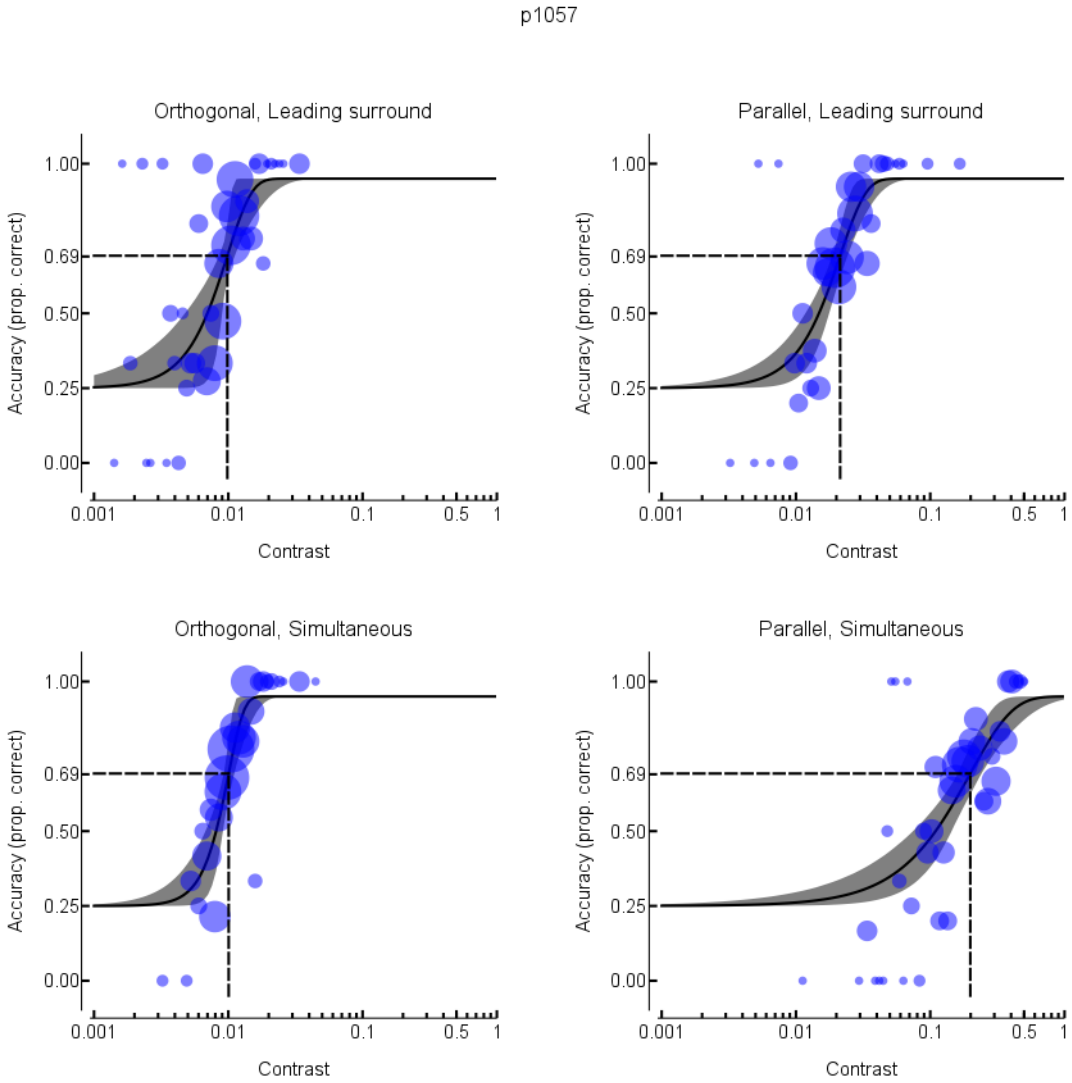
p1057
Orthogonal, Leading surround
Parallel, Leading surround
Orthogonal, Simultaneous
Parallel, Simultaneous
Accuracy (prop. correct)
1.00
0.69
0.50
0.25
0.00
Contrast
0.001
0.01
0.1
0.5
1

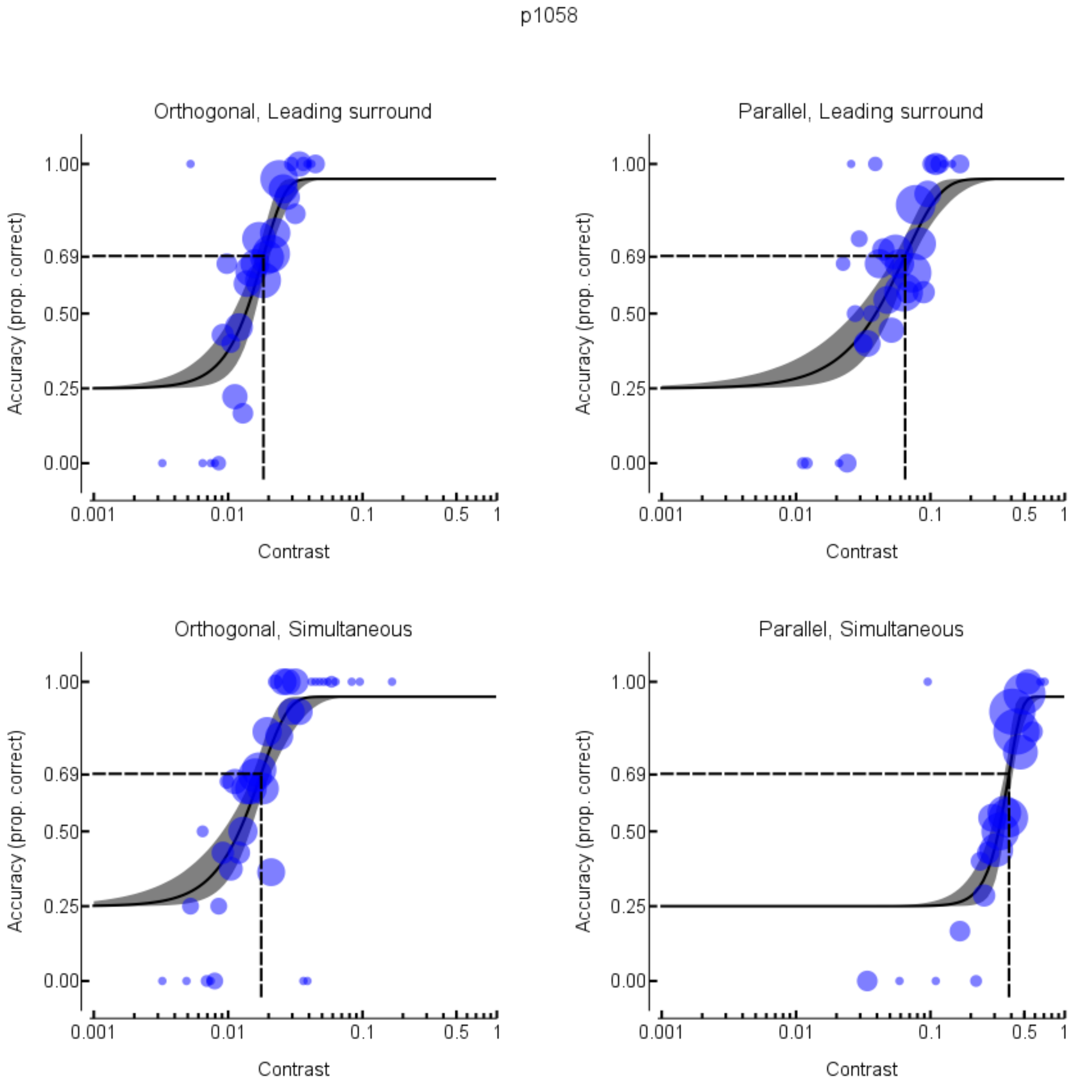
p1058
Orthogonal, Leading surround
Parallel, Leading surround
Orthogonal, Simultaneous
Parallel, Simultaneous
Accuracy (prop. correct)
1.00
0.69
0.50
0.25
0.00
Contrast
0.001
0.01
0.1
0.5
1

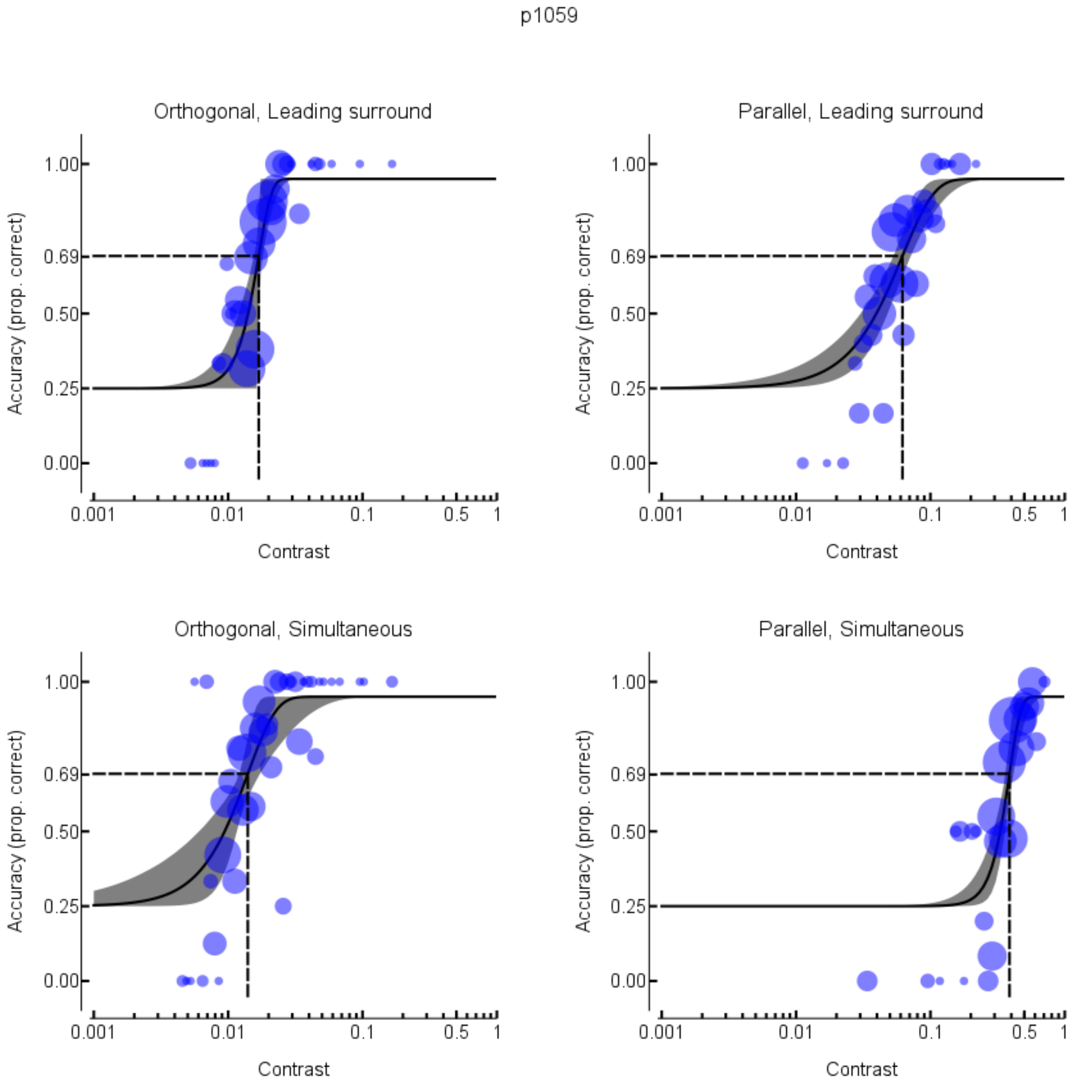
p1059
Orthogonal, Leading surround
Parallel, Leading surround
Orthogonal, Simultaneous
Parallel, Simultaneous
Accuracy (prop. correct)
1.00
0.69
0.50
0.25
0.00
0.001
0.01
0.1
0.5
1
Contrast

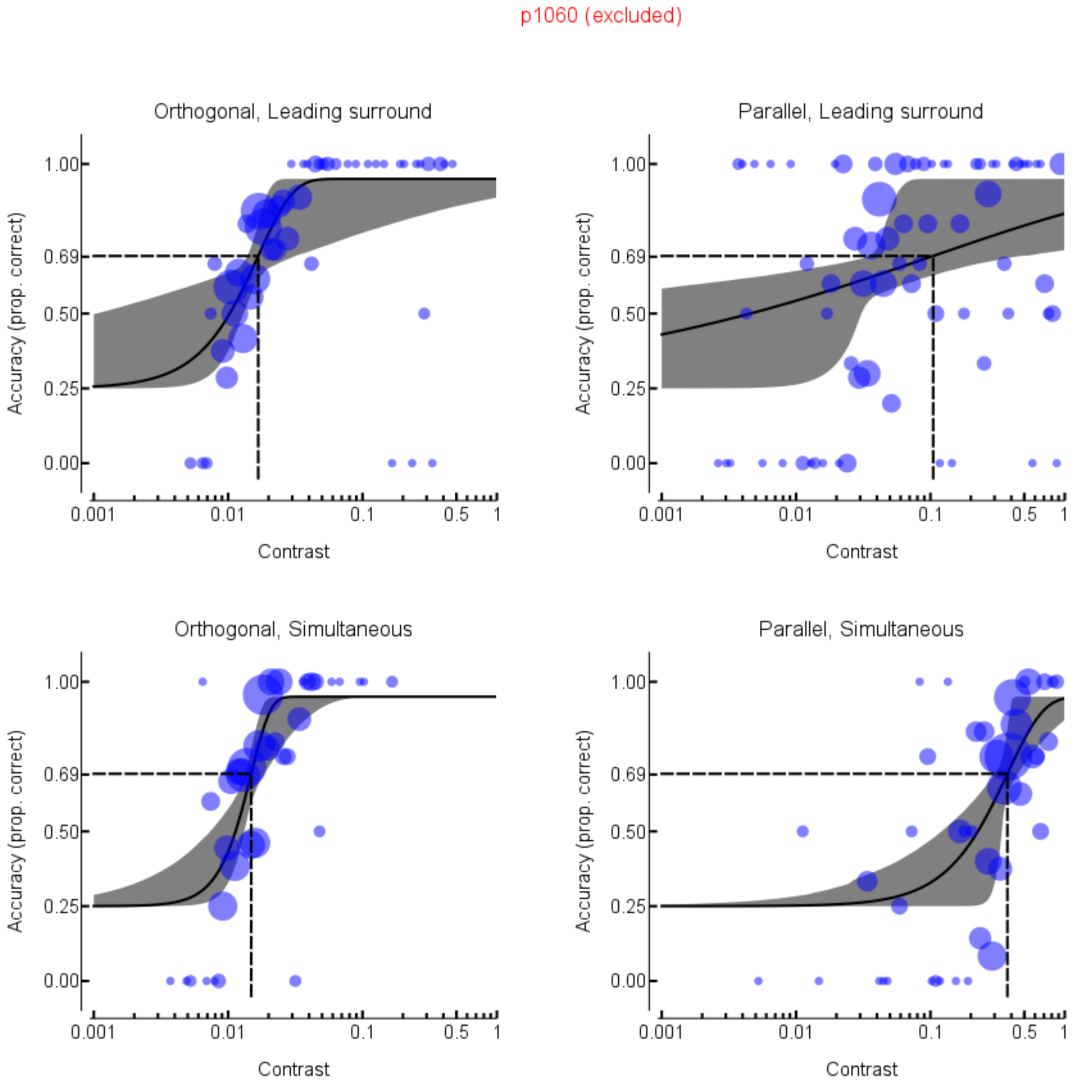
p1060 (excluded)
Orthogonal, Leading surround
Parallel, Leading surround
Orthogonal, Simultaneous
Parallel, Simultaneous
Accuracy (prop. correct)
1.00
0.69
0.50
0.25
0.00
Contrast
0.001
0.01
0.1
0.5
1

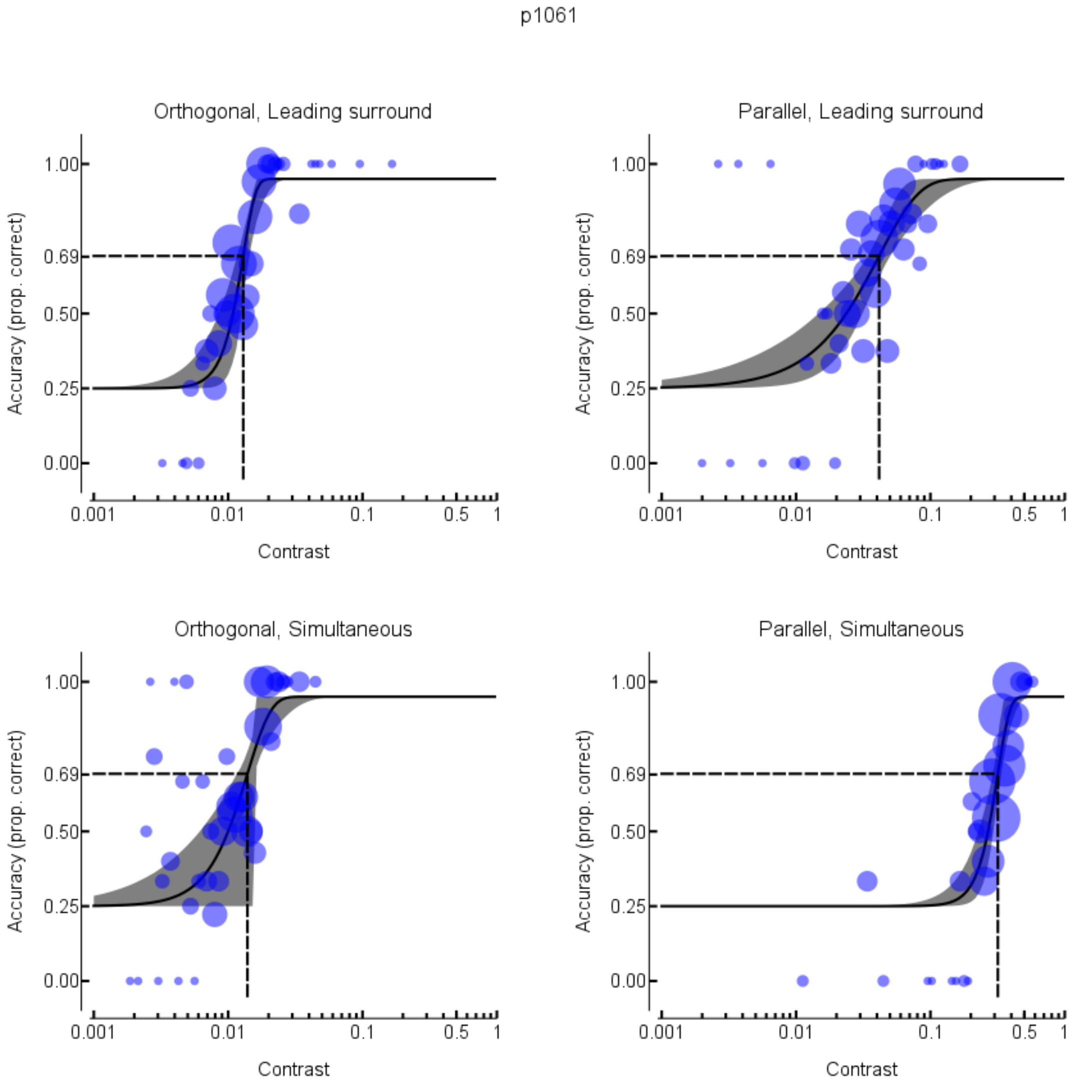
p1061
Orthogonal, Leading surround
Parallel, Leading surround
Orthogonal, Simultaneous
Parallel, Simultaneous
Accuracy (prop. correct)
1.00
0.69
0.50
0.25
0.00
0.001
0.01
0.1
0.5
1
Contrast

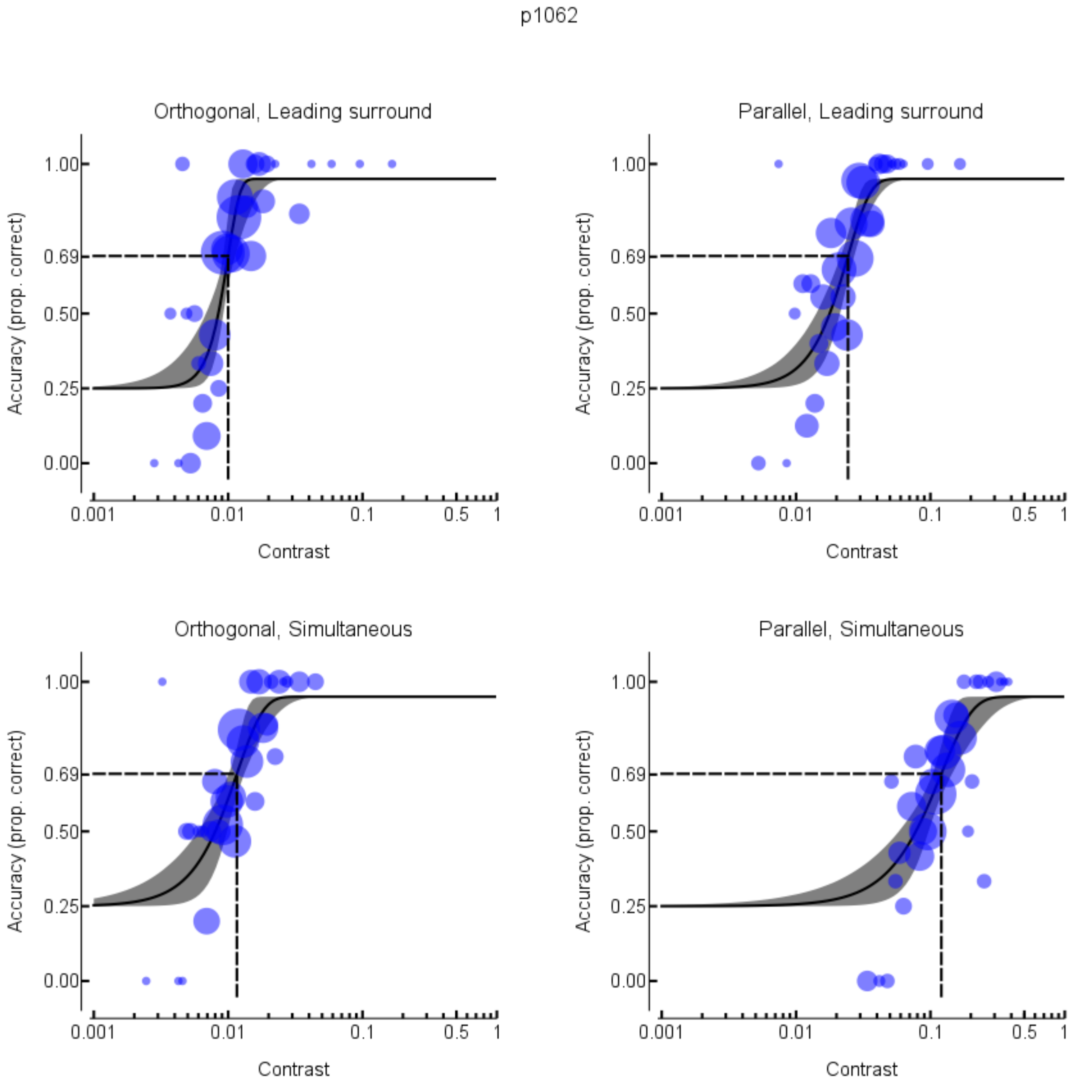
p1062
Orthogonal, Leading surround
Parallel, Leading surround
Orthogonal, Simultaneous
Parallel, Simultaneous
Accuracy (prop. correct)
1.00
0.69
0.50
0.25
0.00
Contrast
0.001
0.01
0.1
0.5
1

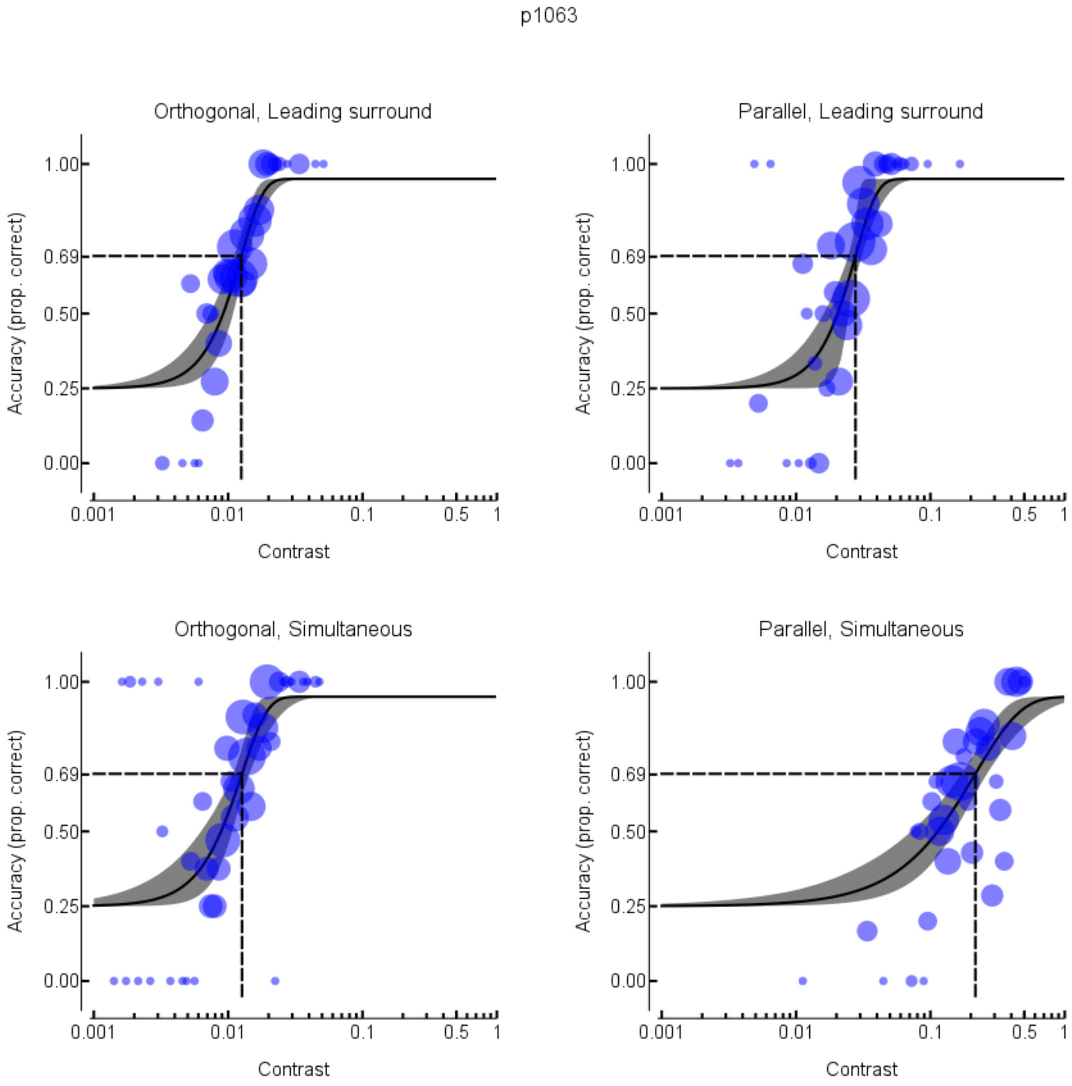
p1063
Orthogonal, Leading surround
Parallel, Leading surround
Orthogonal, Simultaneous
Parallel, Simultaneous
Accuracy (prop. correct)
1.00
0.69
0.50
0.25
0.00
0.001
0.01
0.1
0.5
1
Contrast

p1064

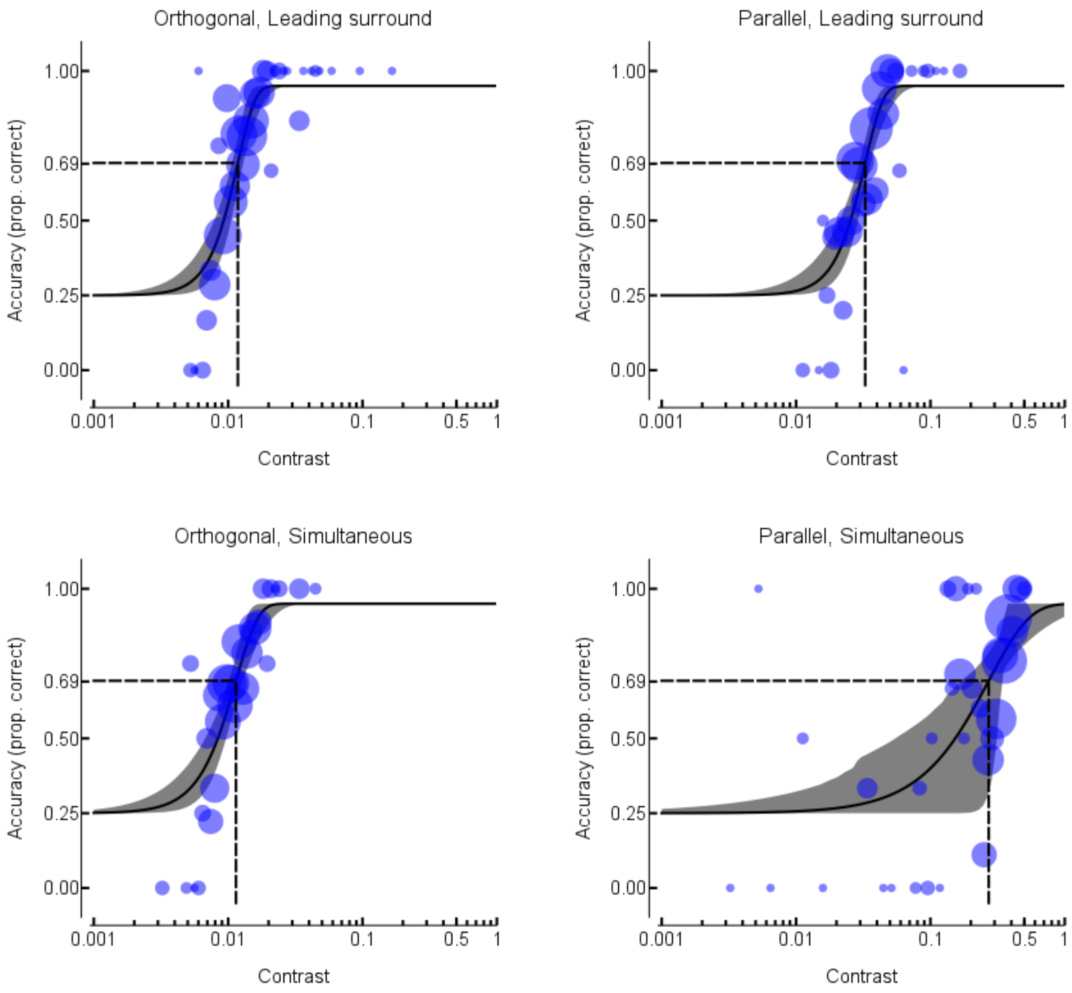
Orthogonal, Leading surround
Parallel, Leading surround
Orthogonal, Simultaneous
Parallel, Simultaneous
Accuracy (prop. correct)
1.00
0.69
0.50
0.25
0.00
0.001
0.01
0.1
0.5
1
Contrast

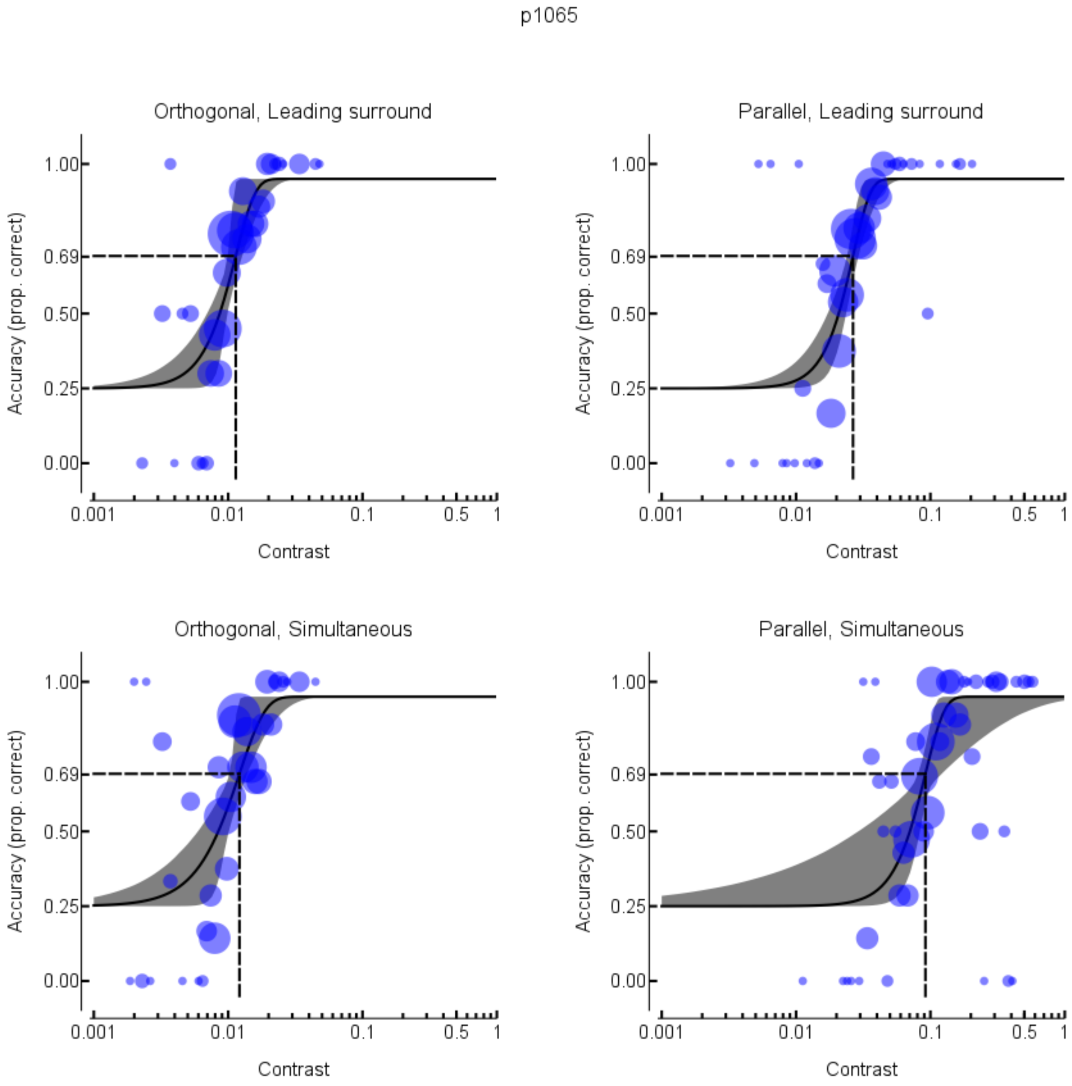
p1065
Orthogonal, Leading surround
Parallel, Leading surround
Orthogonal, Simultaneous
Parallel, Simultaneous
Accuracy (prop. correct)
1.00
0.69
0.50
0.25
0.00
0.001
0.01
0.1
0.5
1
Contrast

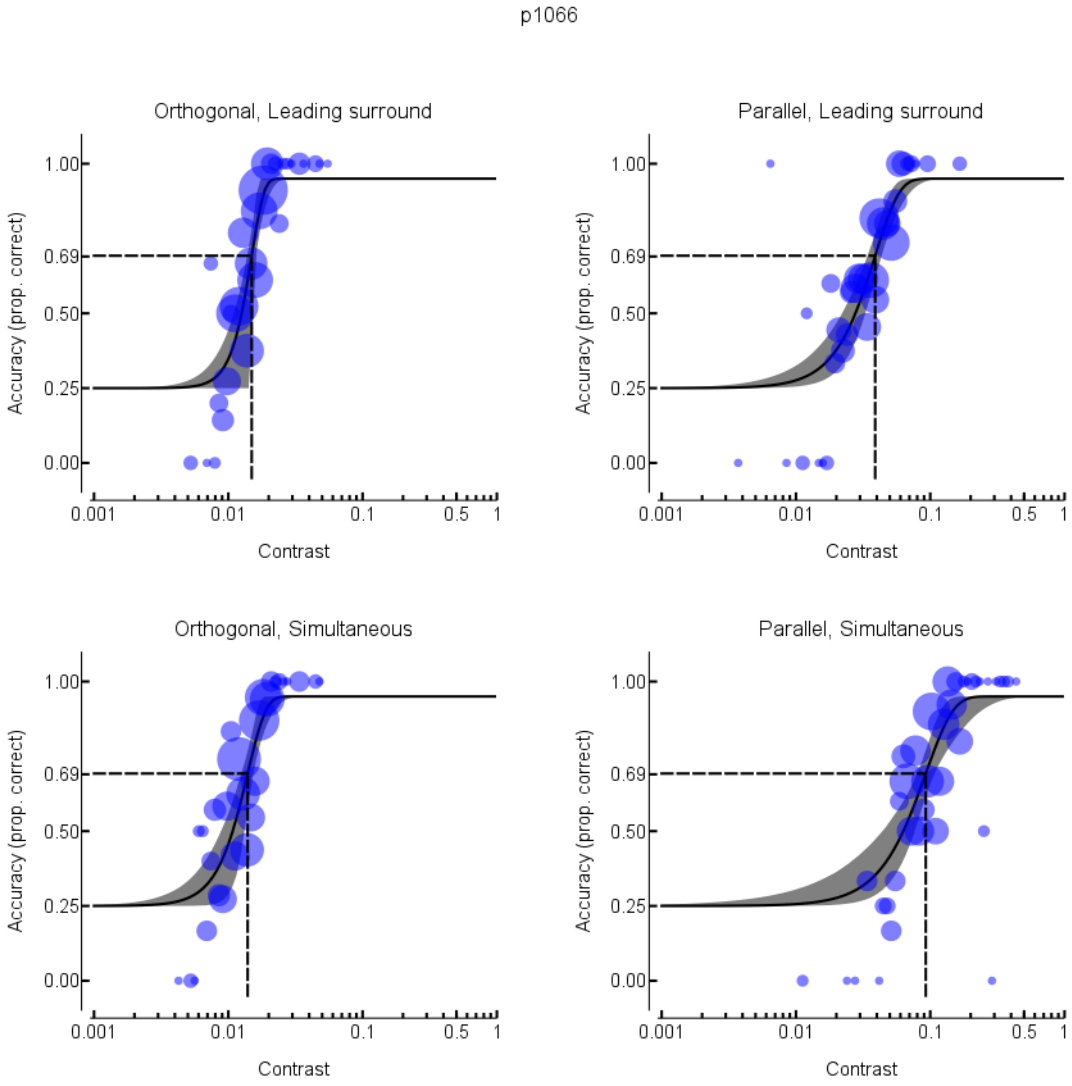
p1066
Orthogonal, Leading surround
Parallel, Leading surround
Orthogonal, Simultaneous
Parallel, Simultaneous
Accuracy (prop. correct)
1.00
0.69
0.50
0.25
0.00
0.001
0.01
0.1
0.5
1
Contrast

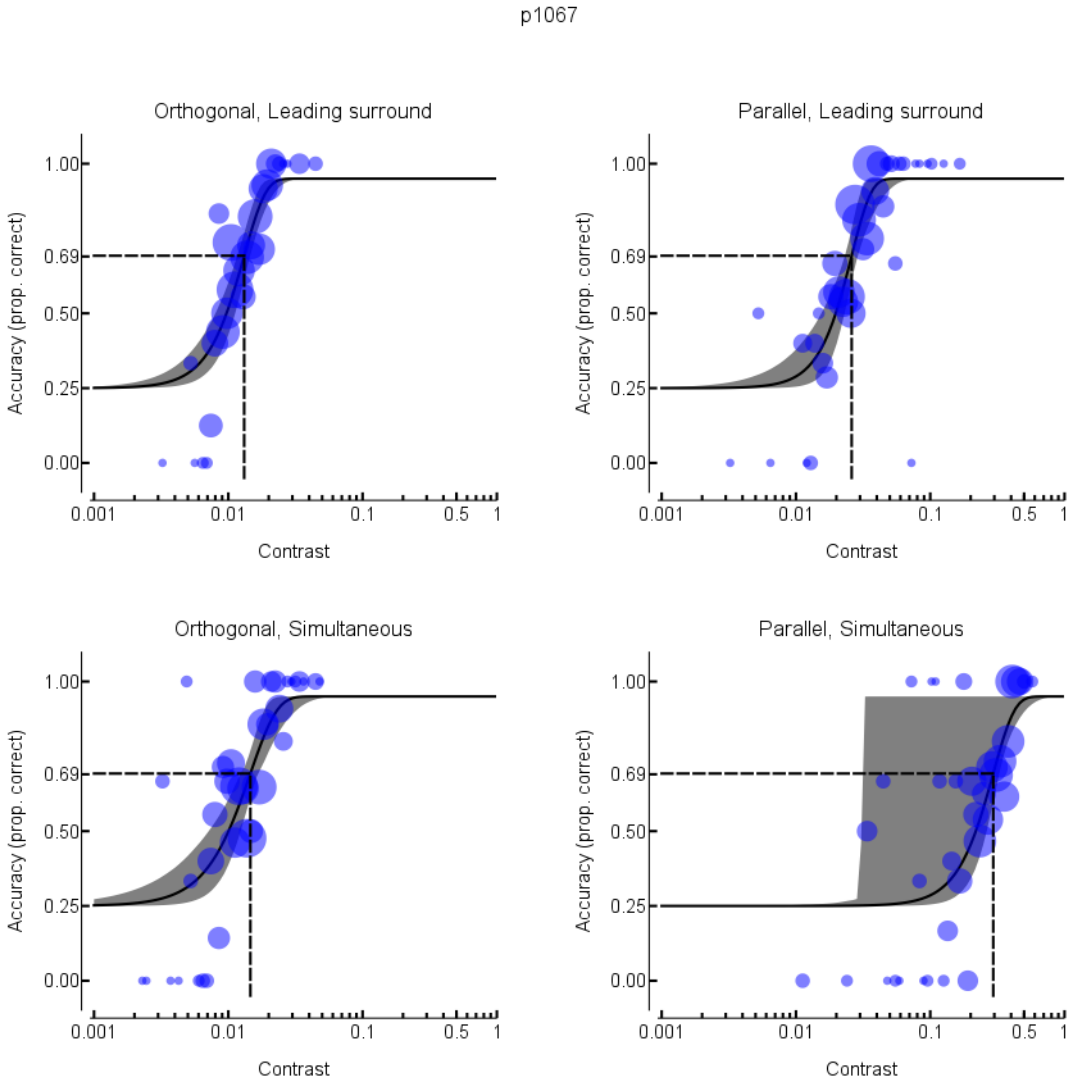
p1067
Orthogonal, Leading surround
Parallel, Leading surround
Orthogonal, Simultaneous
Parallel, Simultaneous
Accuracy (prop. correct)
1.00
0.69
0.50
0.25
0.00
0.001
0.01
0.1
0.5
1
Contrast

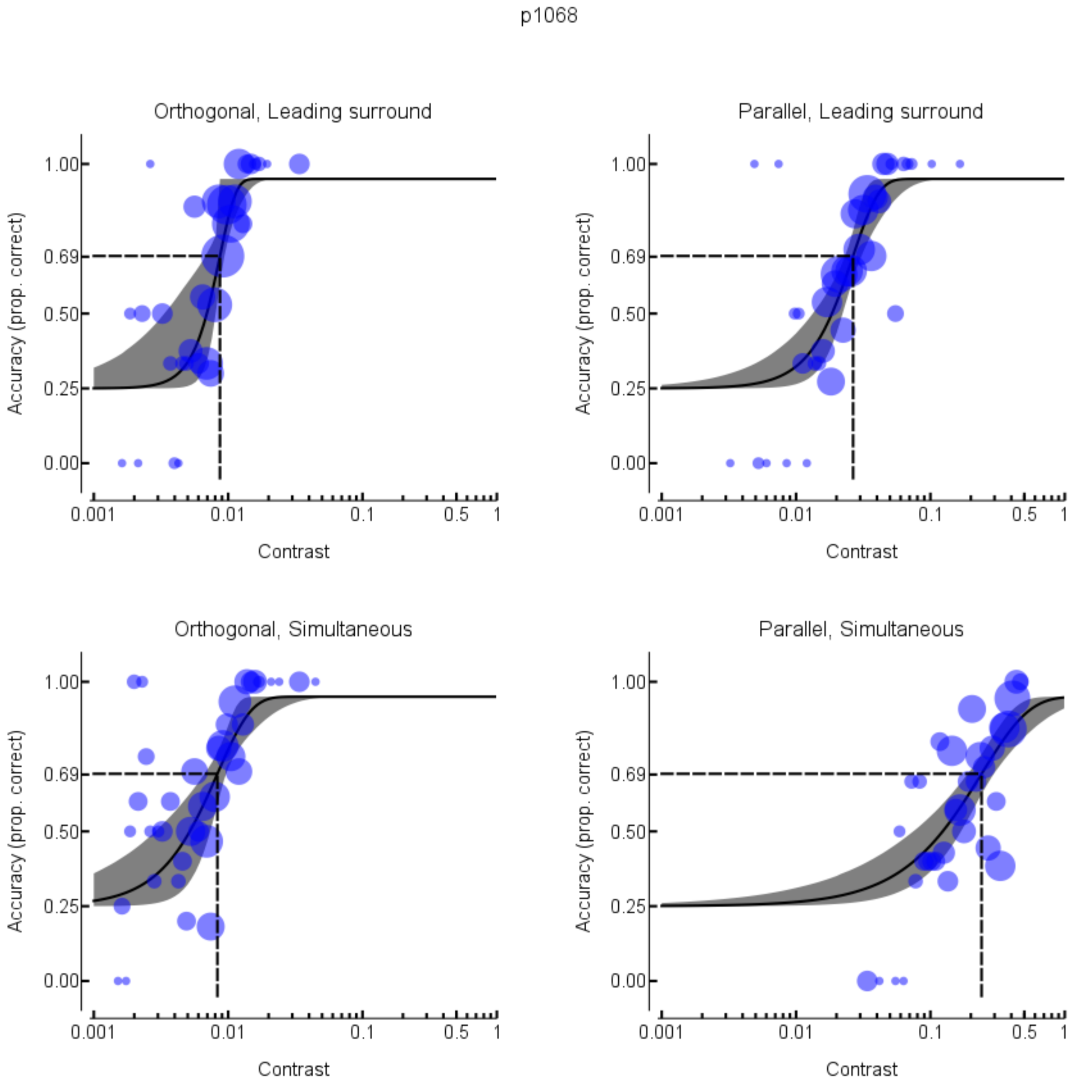
p1068
Orthogonal, Leading surround
Parallel, Leading surround
Orthogonal, Simultaneous
Parallel, Simultaneous
Accuracy (prop. correct)
1.00
0.69
0.50
0.25
0.00
Contrast
0.001
0.01
0.1
0.5
1

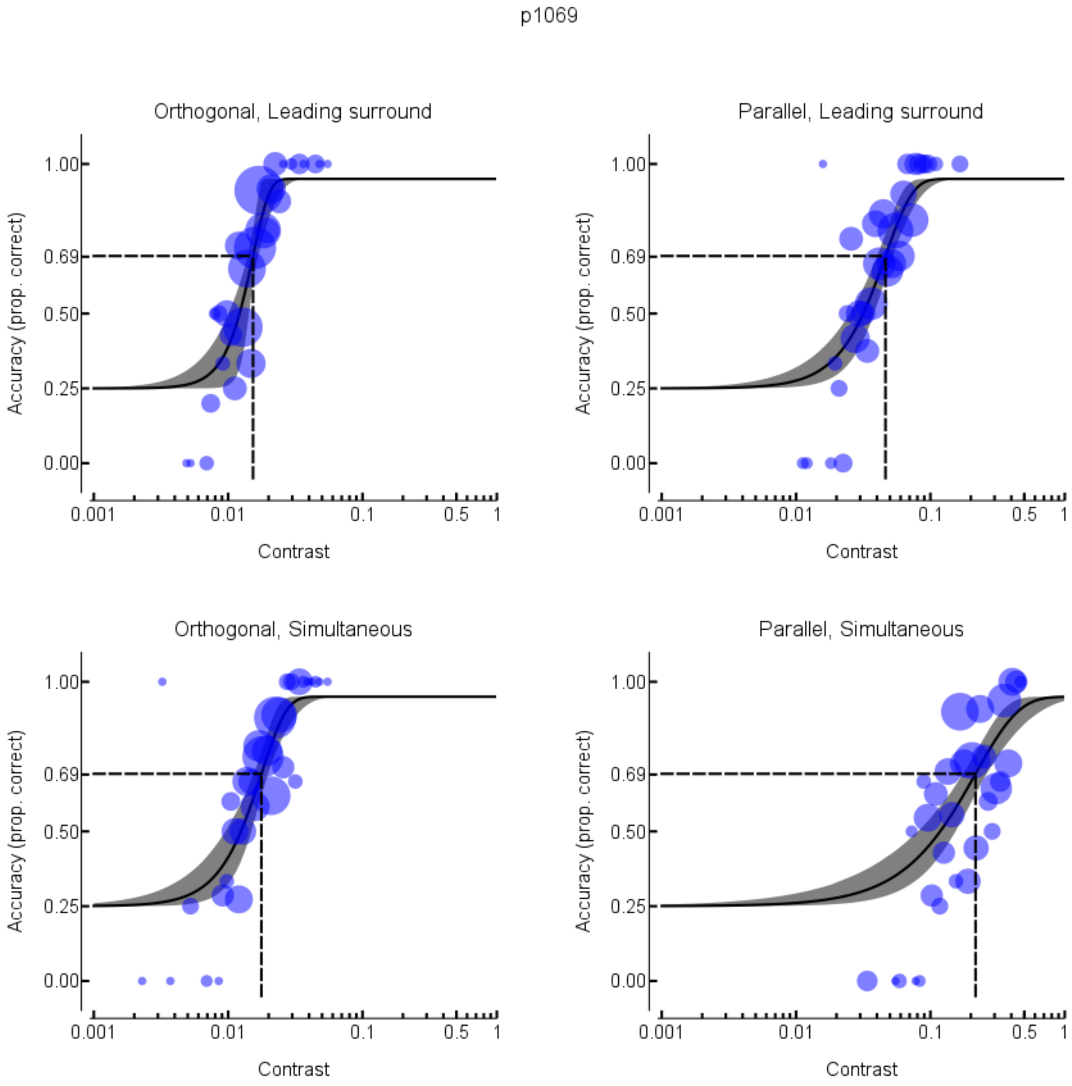
p1069
Orthogonal, Leading surround
Parallel, Leading surround
Orthogonal, Simultaneous
Parallel, Simultaneous
Accuracy (prop. correct)
1.00
0.69
0.50
0.25
0.00
Contrast
0.001
0.01
0.1
0.5
1

p1070

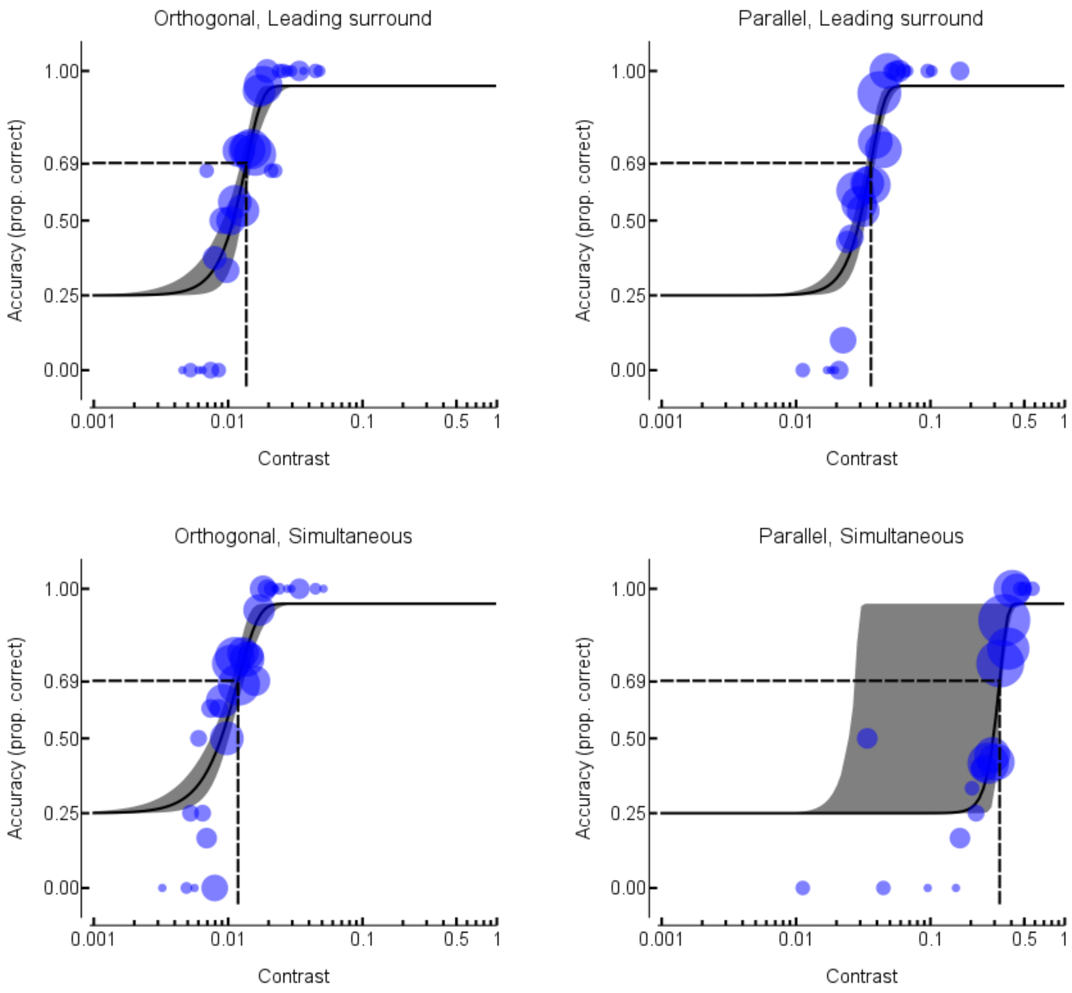
Orthogonal, Leading surround
Parallel, Leading surround
Orthogonal, Simultaneous
Parallel, Simultaneous
Accuracy (prop. correct)
1.00
0.69
0.50
0.25
0.00
Contrast
0.001
0.01
0.1
0.5
1

p1071

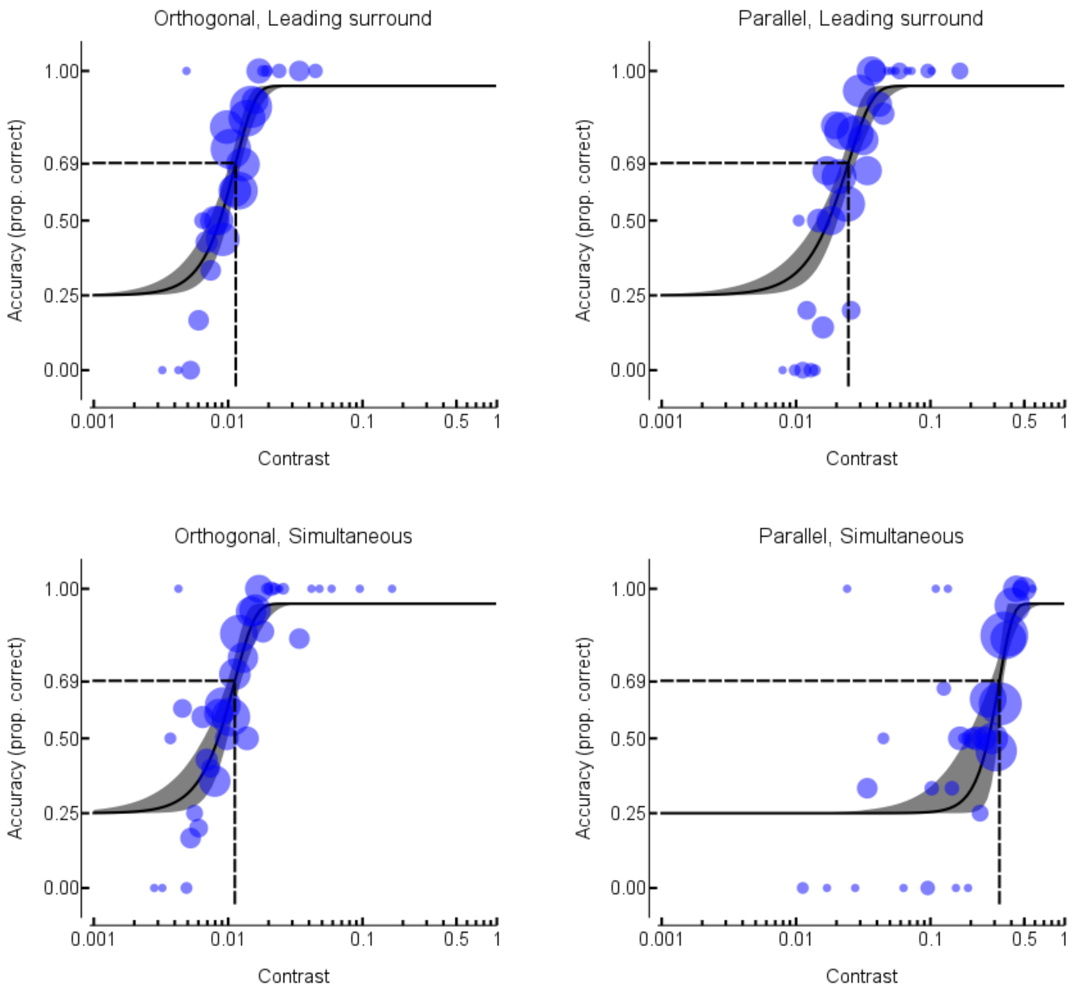
Orthogonal, Leading surround
Parallel, Leading surround
Orthogonal, Simultaneous
Parallel, Simultaneous
Accuracy (prop. correct)
1.00
0.69
0.50
0.25
0.00
0.001
0.01
0.1
0.5
1
Contrast

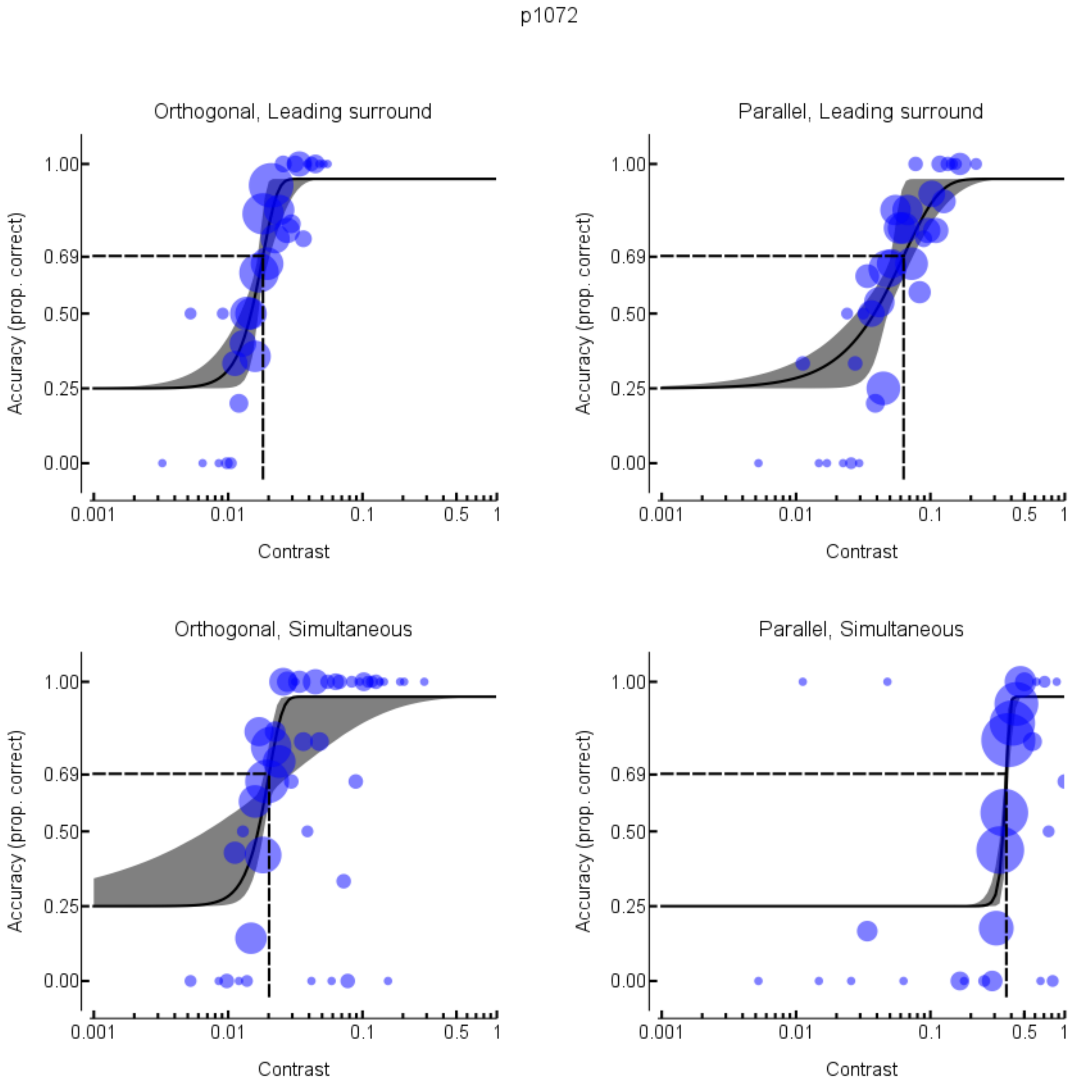
p1072
Orthogonal, Leading surround
Parallel, Leading surround
Orthogonal, Simultaneous
Parallel, Simultaneous
Accuracy (prop. correct)
1.00
0.69
0.50
0.25
0.00
0.001
0.01
0.1
0.5
1
Contrast

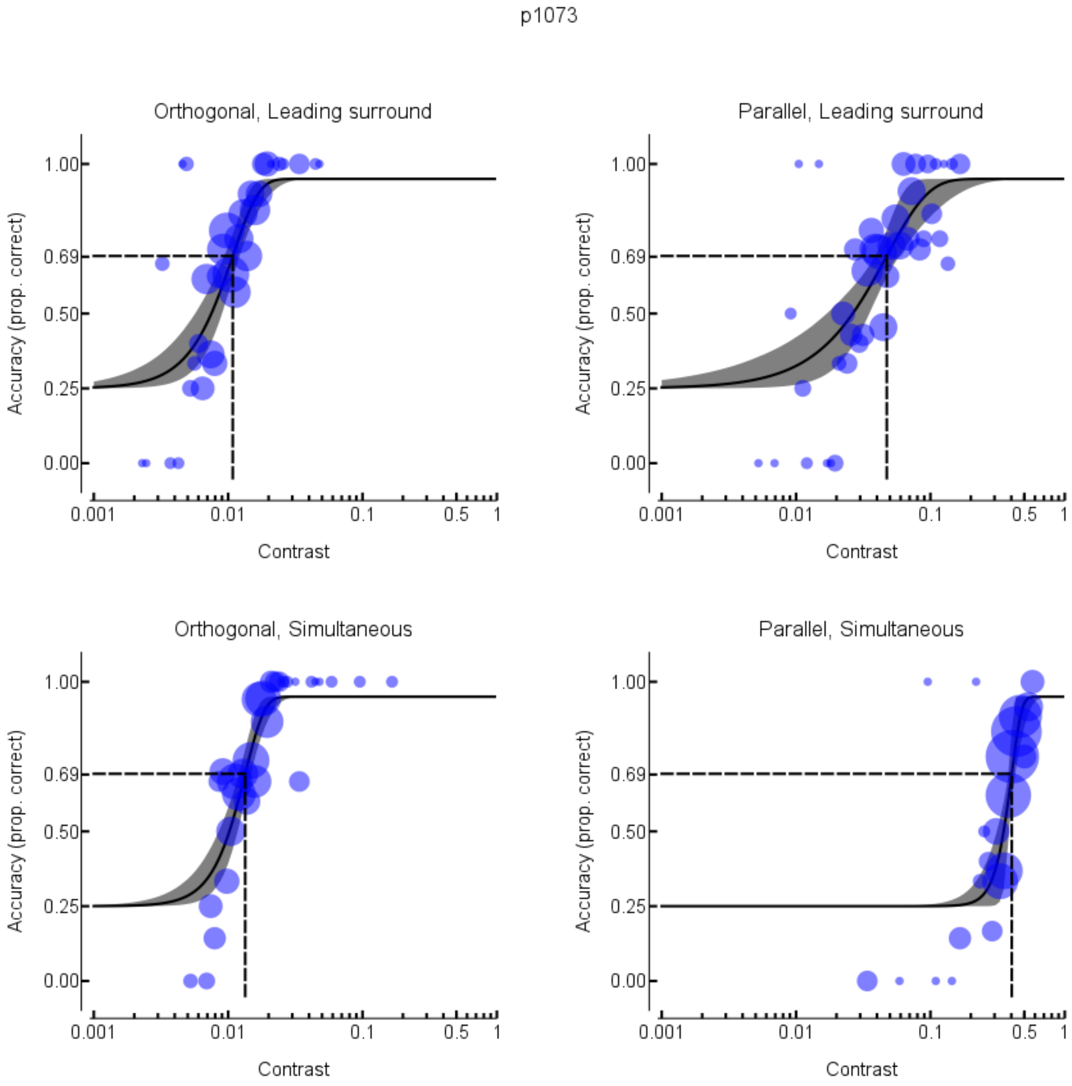
p1073
Orthogonal, Leading surround
Parallel, Leading surround
Orthogonal, Simultaneous
Parallel, Simultaneous
Accuracy (prop. correct)
1.00
0.69
0.50
0.25
0.00
0.001
0.01
0.1
0.5
1
Contrast

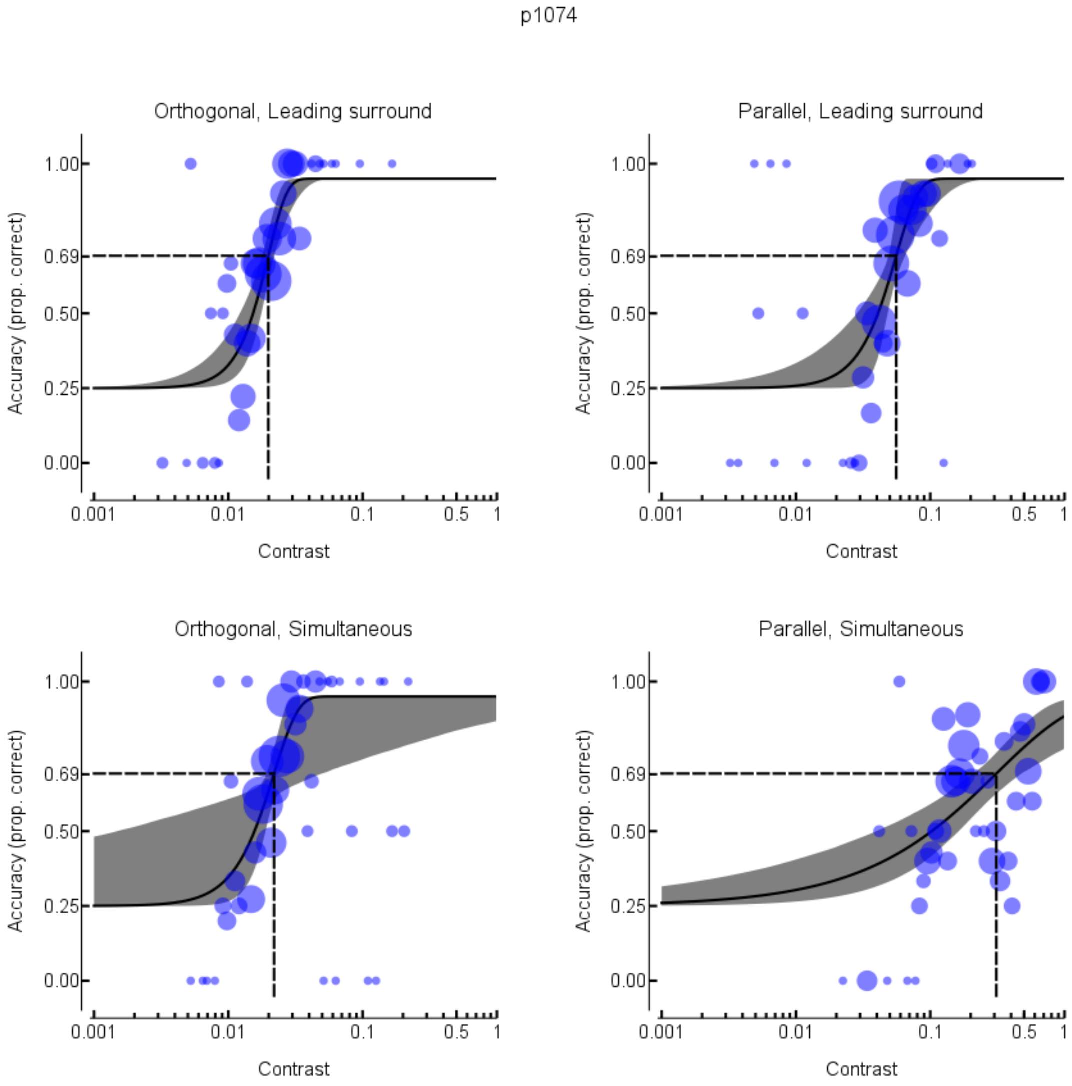
p1074
Orthogonal, Leading surround
Parallel, Leading surround
Orthogonal, Simultaneous
Parallel, Simultaneous
Accuracy (prop. correct)
1.00
0.69
0.50
0.25
0.00
0.001
0.01
0.1
0.5
1
Contrast

p1075

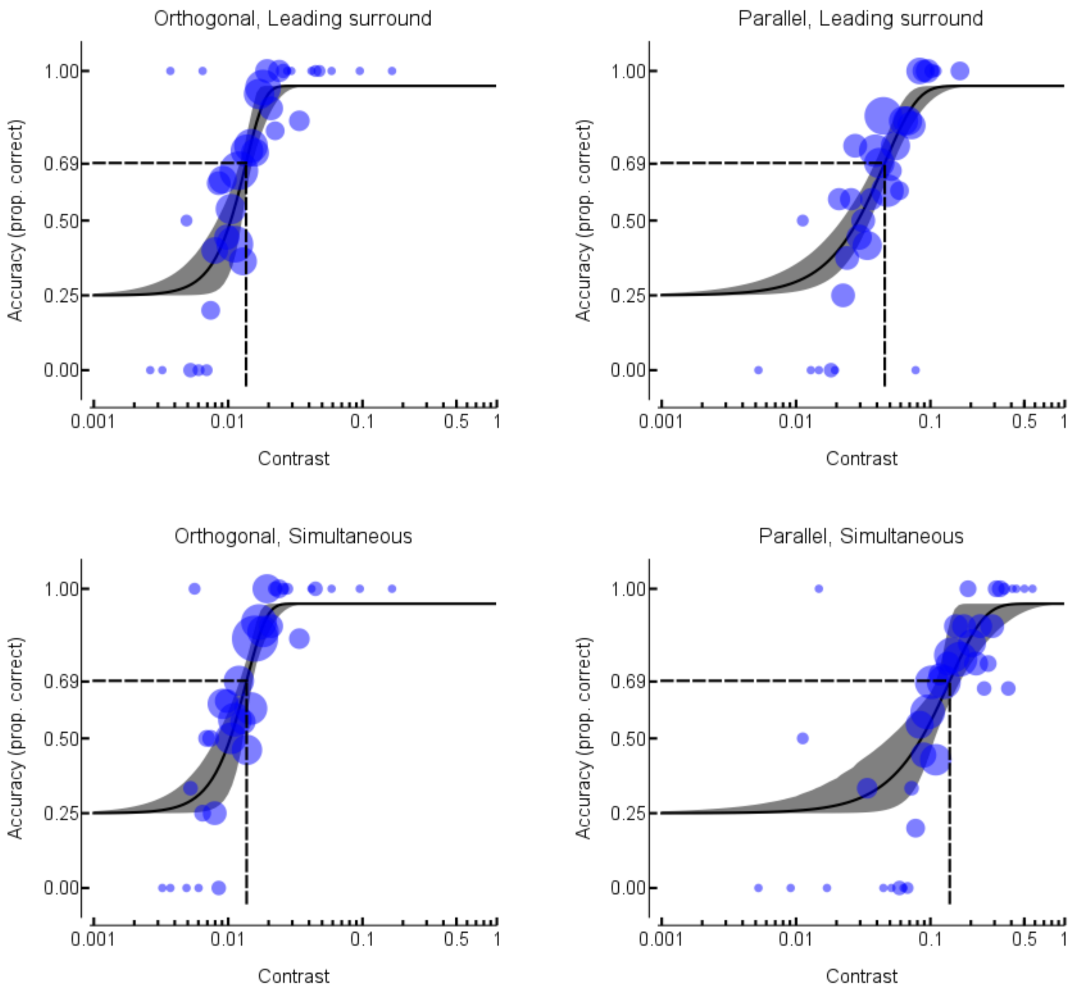
Orthogonal, Leading surround
Parallel, Leading surround
Orthogonal, Simultaneous
Parallel, Simultaneous
Accuracy (prop. correct)
1.00
0.69
0.50
0.25
0.00
0.001
0.01
0.1
0.5
1
Contrast

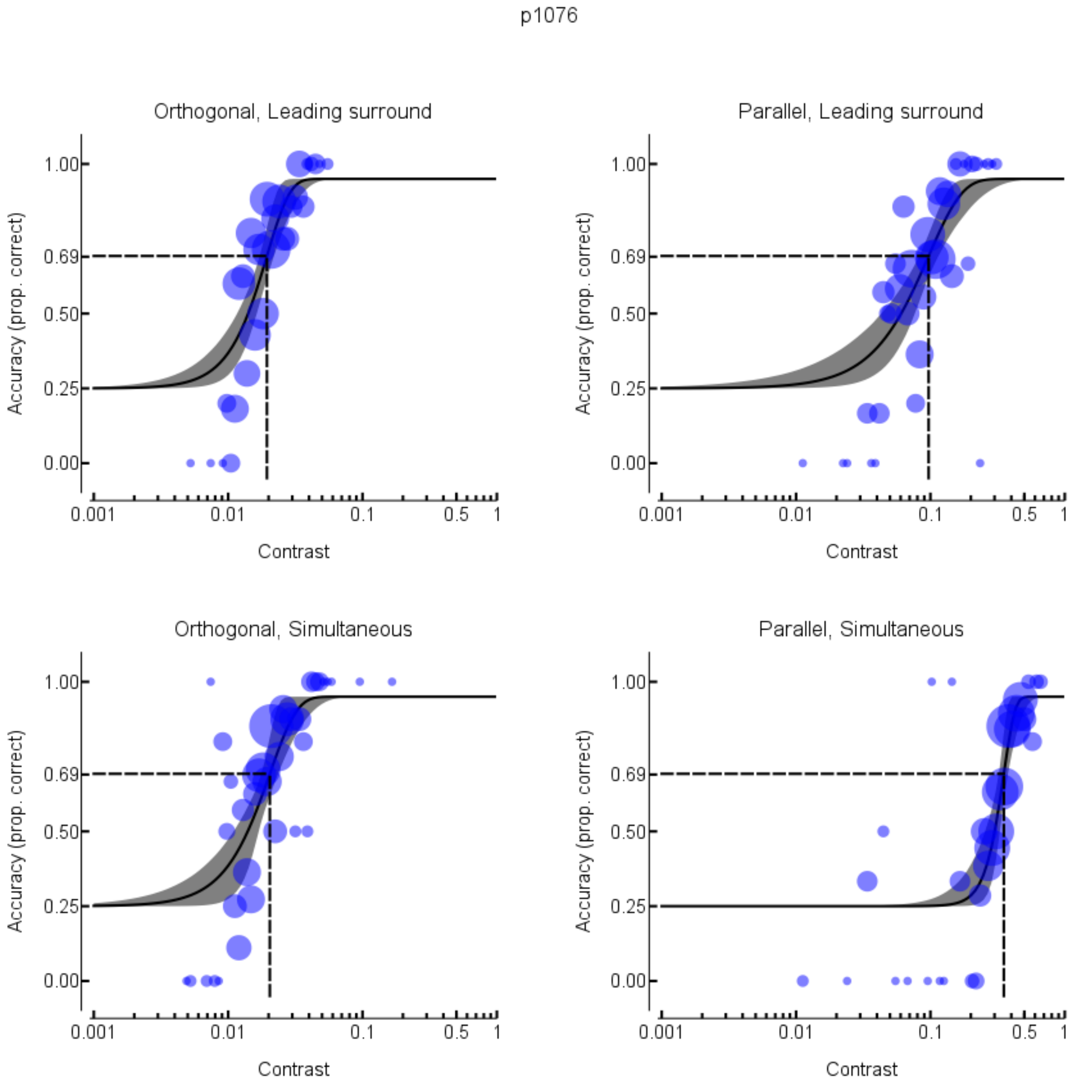
p1076
Orthogonal, Leading surround
Parallel, Leading surround
Orthogonal, Simultaneous
Parallel, Simultaneous
Accuracy (prop. correct)
1.00
0.69
0.50
0.25
0.00
Contrast
0.001
0.01
0.1
0.5
1

p1077

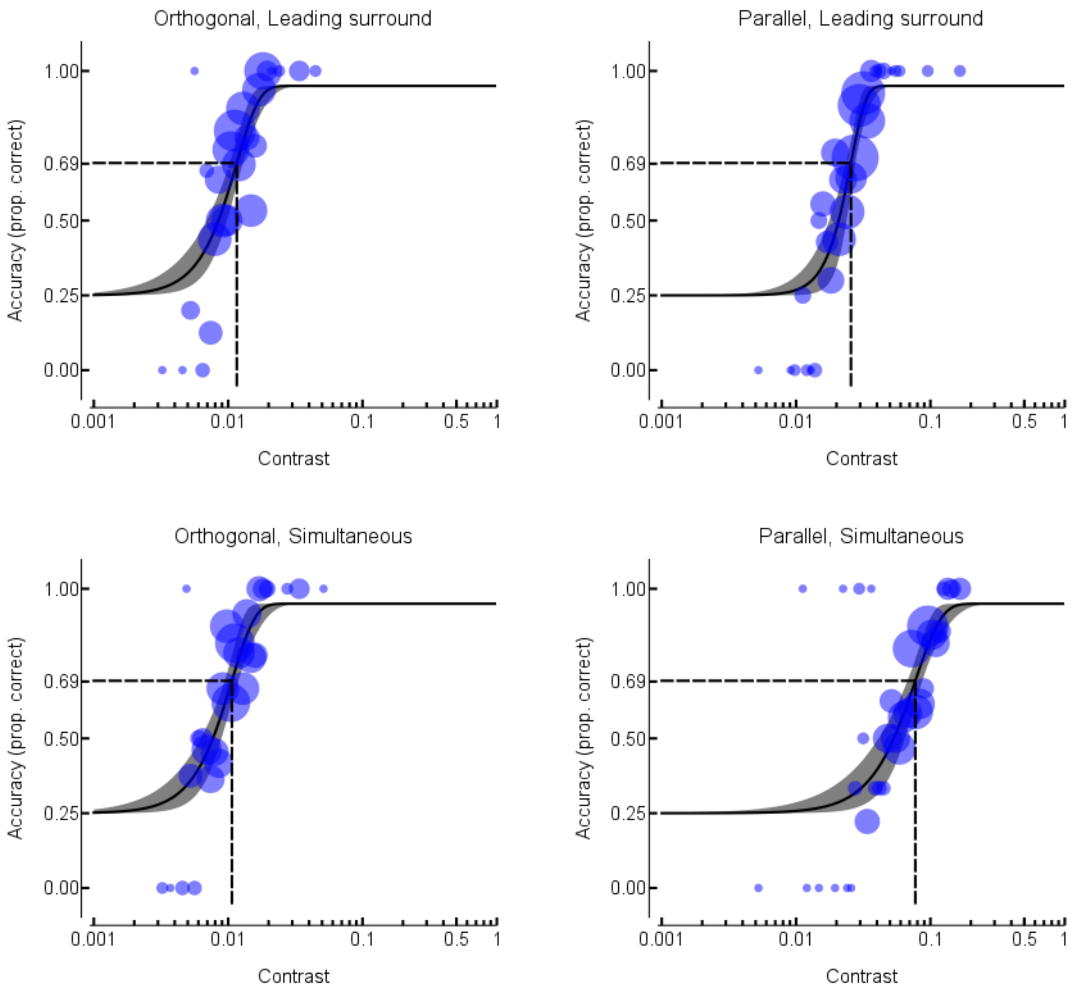
Orthogonal, Leading surround
Parallel, Leading surround
Orthogonal, Simultaneous
Parallel, Simultaneous
Accuracy (prop. correct)
1.00
0.69
0.50
0.25
0.00
0.001
0.01
0.1
0.5
1
Contrast

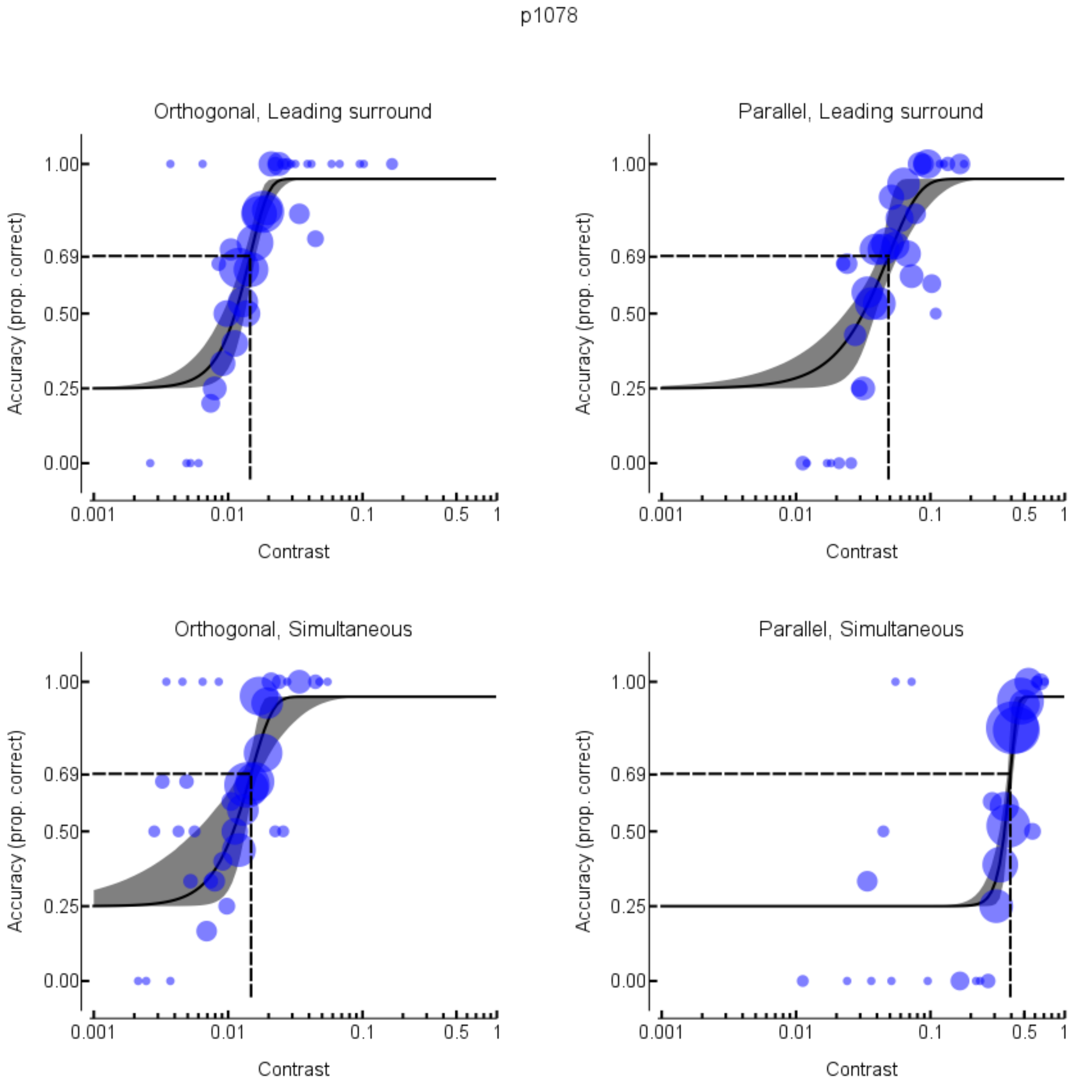
p1078
Orthogonal, Leading surround
Parallel, Leading surround
Orthogonal, Simultaneous
Parallel, Simultaneous
Accuracy (prop. correct)
1.00
0.69
0.50
0.25
0.00
Contrast
0.001
0.01
0.1
0.5
1

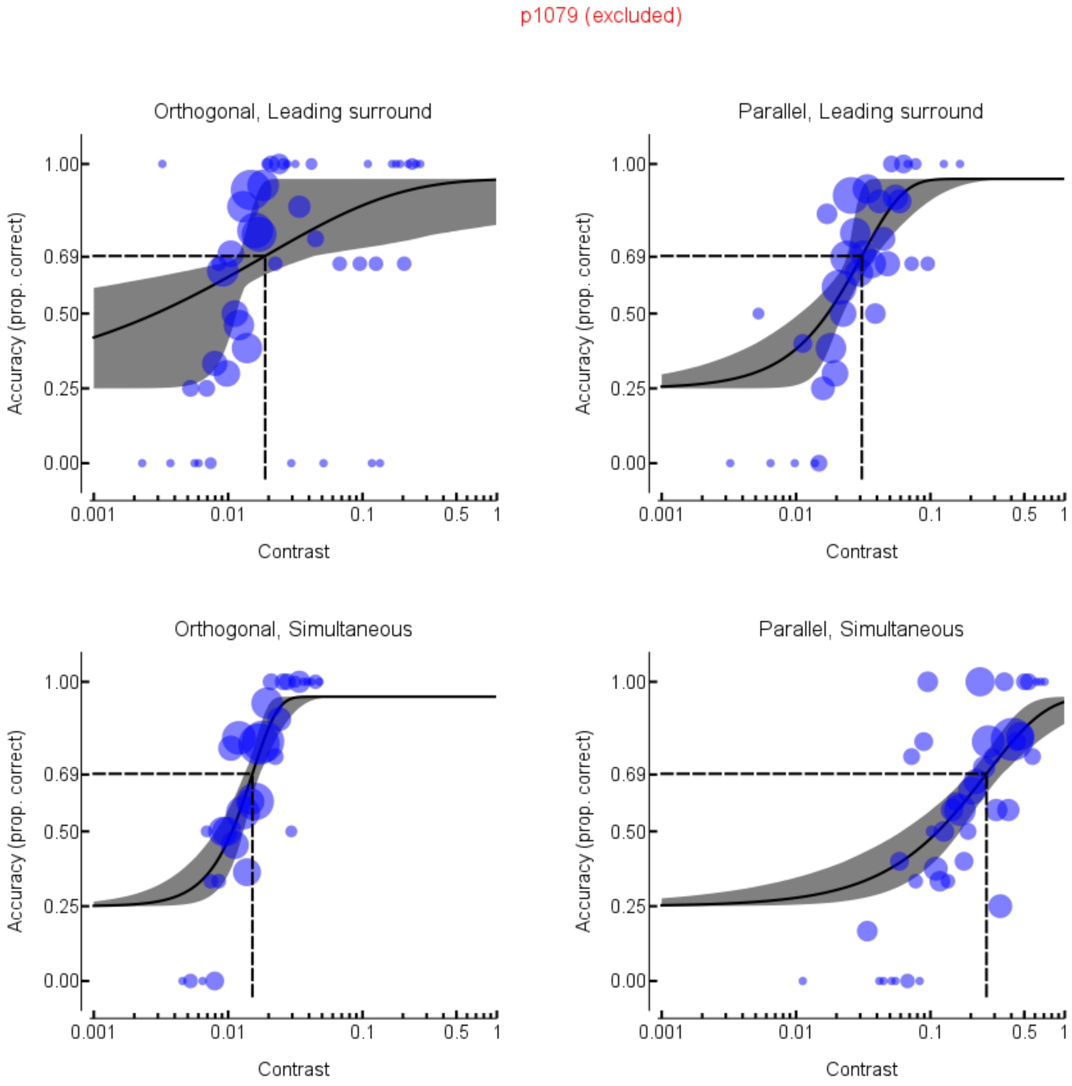
p1079 (excluded)
Orthogonal, Leading surround
Parallel, Leading surround
Orthogonal, Simultaneous
Parallel, Simultaneous
Accuracy (prop. correct)
1.00
0.69
0.50
0.25
0.00
0.001
0.01
0.1
0.5
1
Contrast

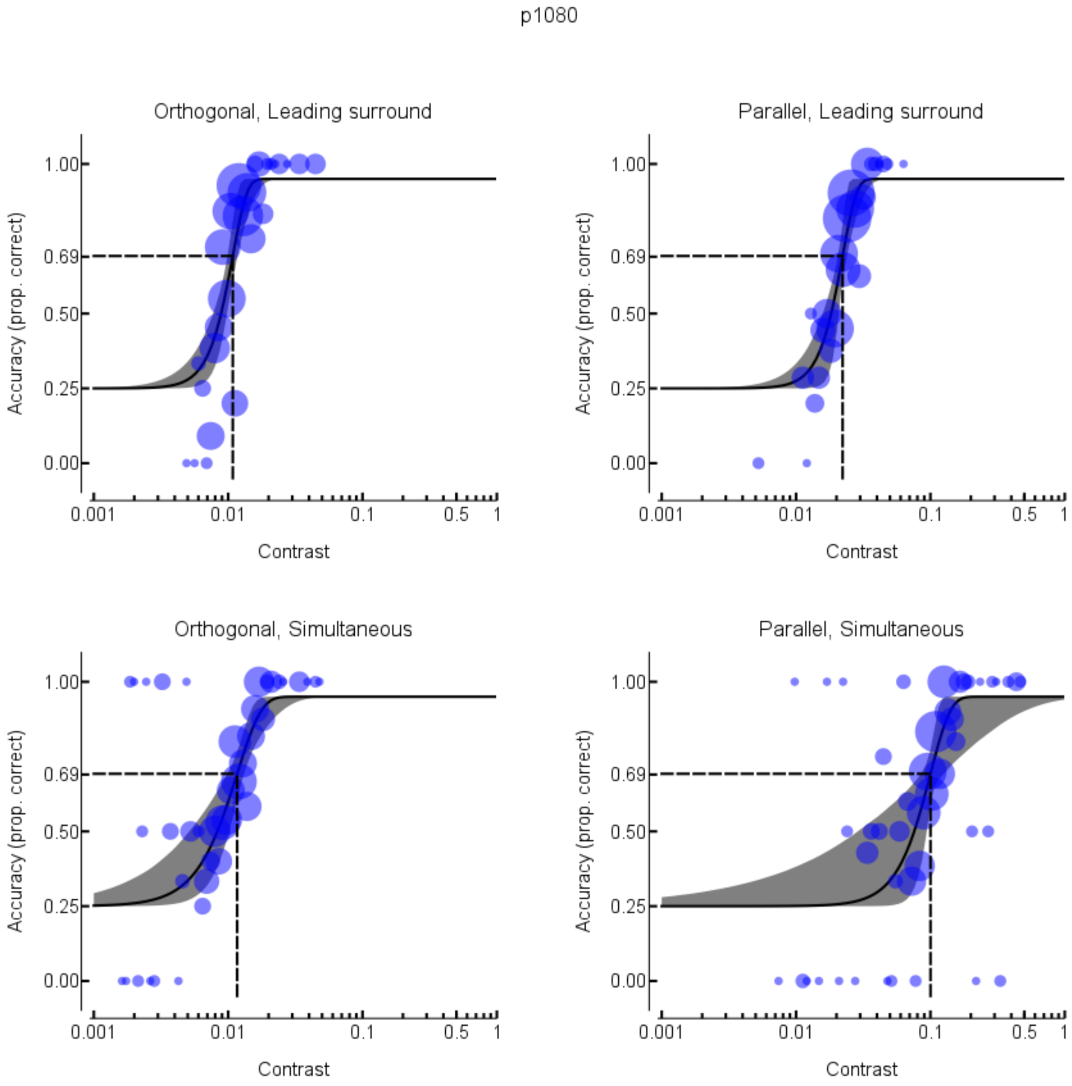
p1080
Orthogonal, Leading surround
Parallel, Leading surround
Orthogonal, Simultaneous
Parallel, Simultaneous
Accuracy (prop. correct)
1.00
0.69
0.50
0.25
0.00
0.001
0.01
0.1
0.5
1
Contrast

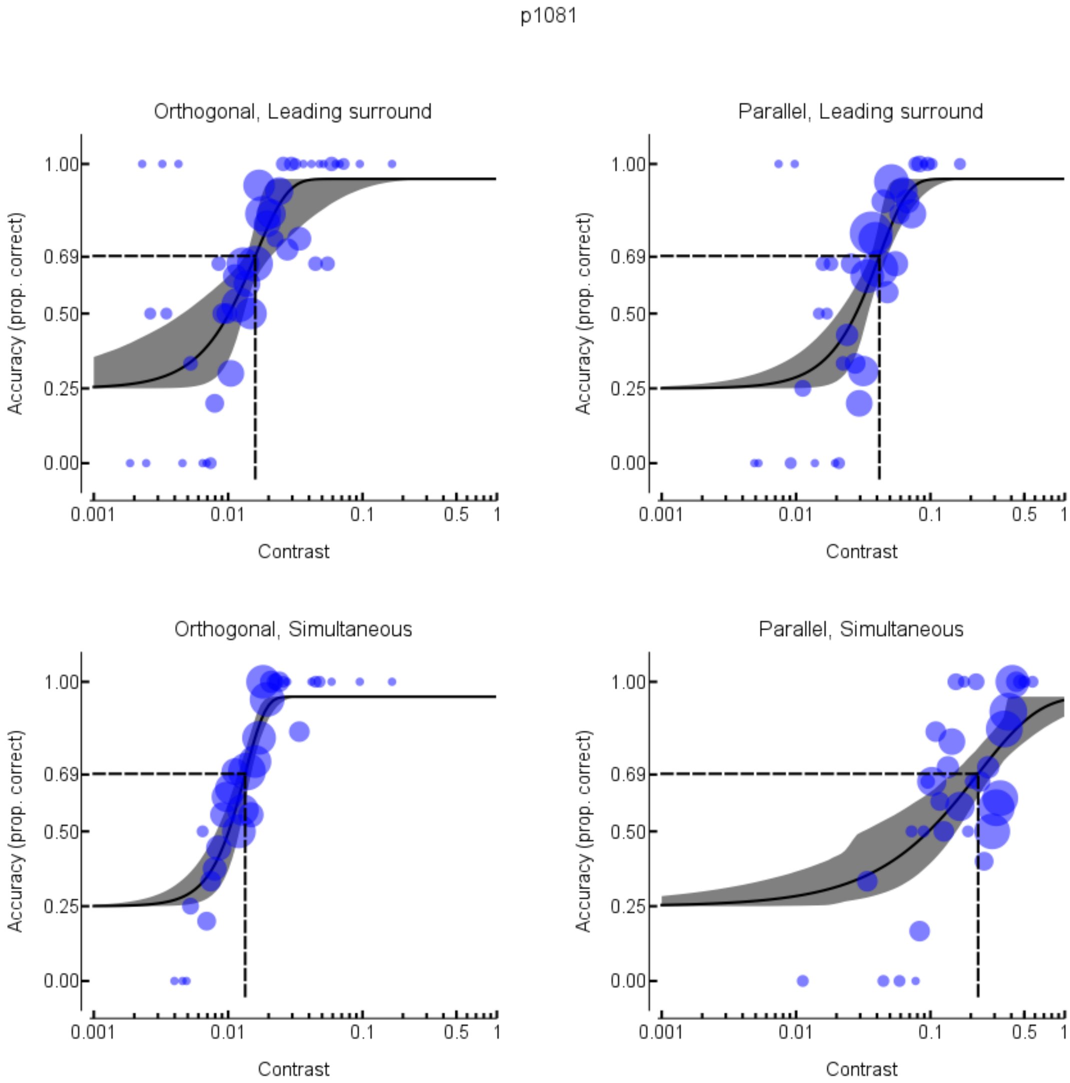
p1081
Orthogonal, Leading surround
Parallel, Leading surround
Orthogonal, Simultaneous
Parallel, Simultaneous
Accuracy (prop. correct)
1.00
0.69
0.50
0.25
0.00
0.001
0.01
0.1
0.5
1
Contrast

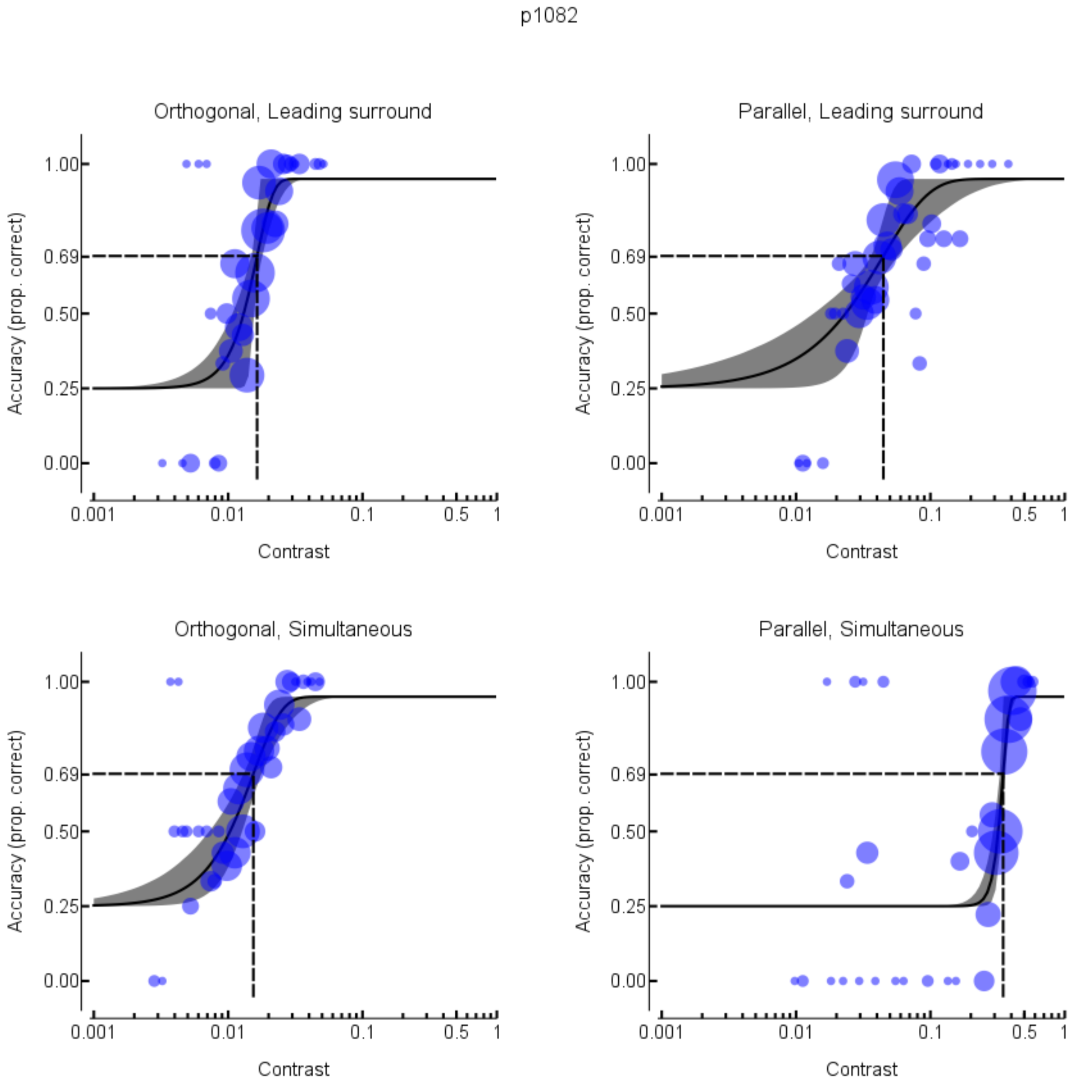
p1082
Orthogonal, Leading surround
Parallel, Leading surround
Orthogonal, Simultaneous
Parallel, Simultaneous
Accuracy (prop. correct)
1.00
0.69
0.50
0.25
0.00
0.001
0.01
0.1
0.5
1
Contrast

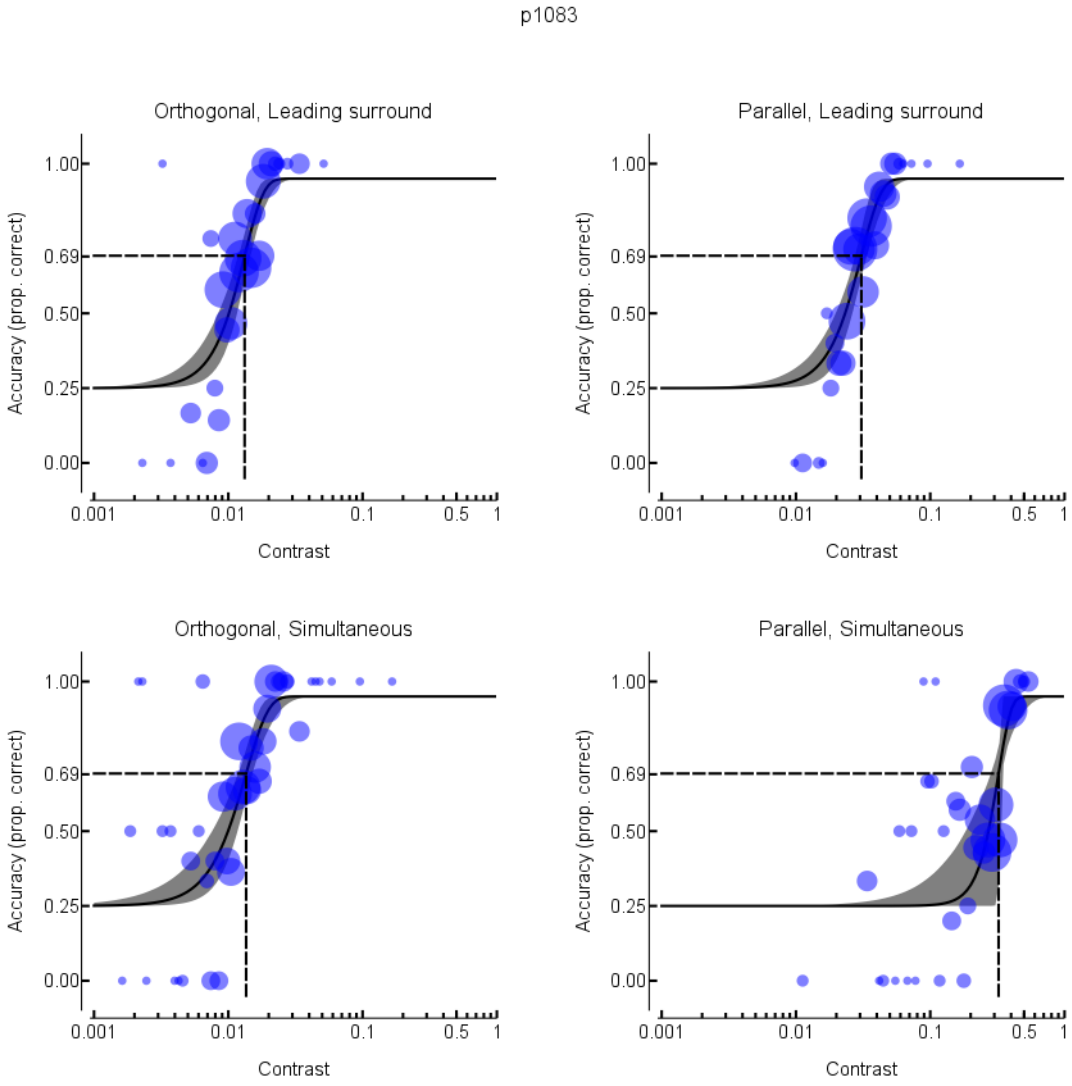
p1083
Orthogonal, Leading surround
Parallel, Leading surround
Orthogonal, Simultaneous
Parallel, Simultaneous
Accuracy (prop. correct)
1.00
0.69
0.50
0.25
0.00
0.001
0.01
0.1
0.5
1
Contrast

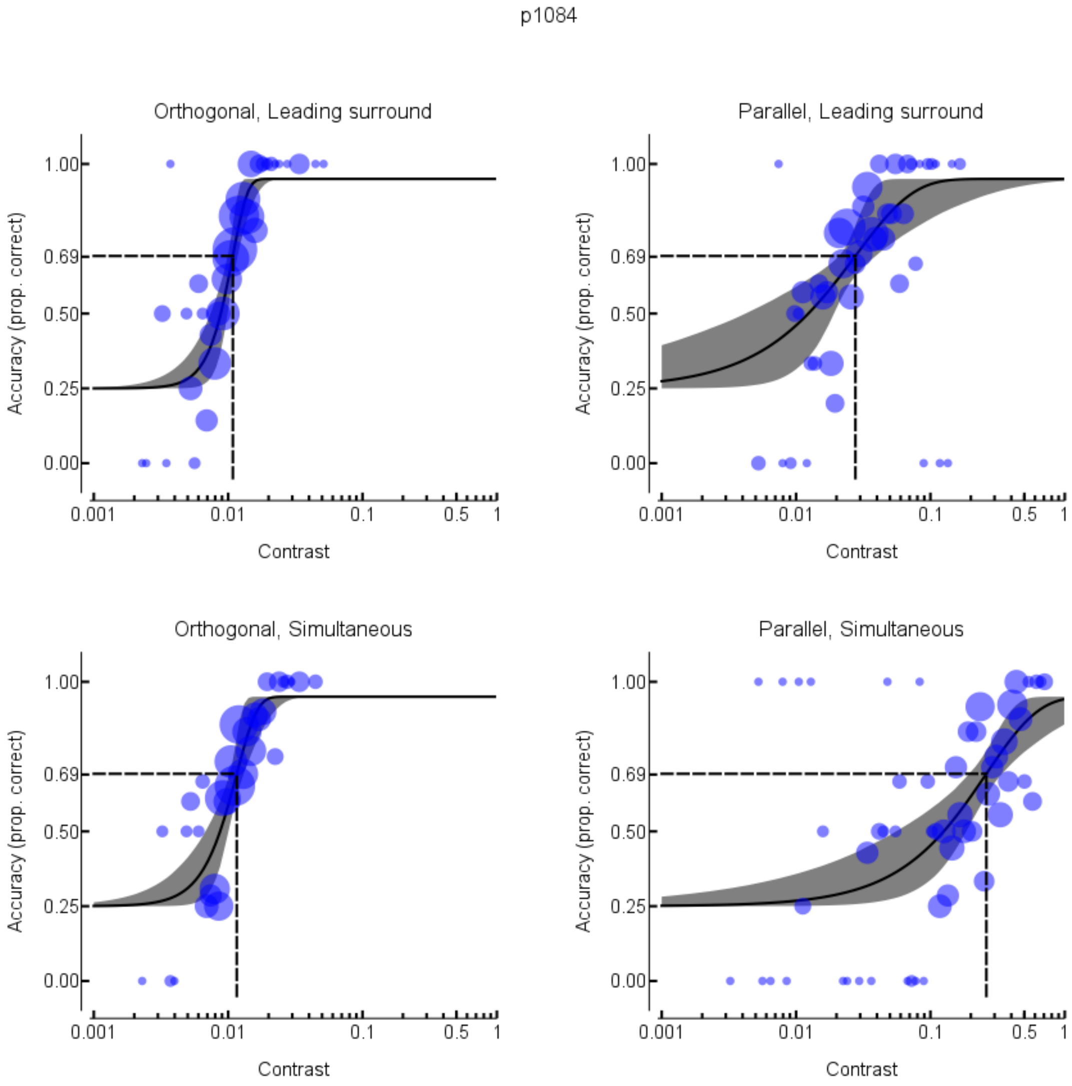
p1084
Orthogonal, Leading surround
Parallel, Leading surround
Orthogonal, Simultaneous
Parallel, Simultaneous
Accuracy (prop. correct)
1.00
0.69
0.50
0.25
0.00
Contrast
0.001
0.01
0.1
0.5
1

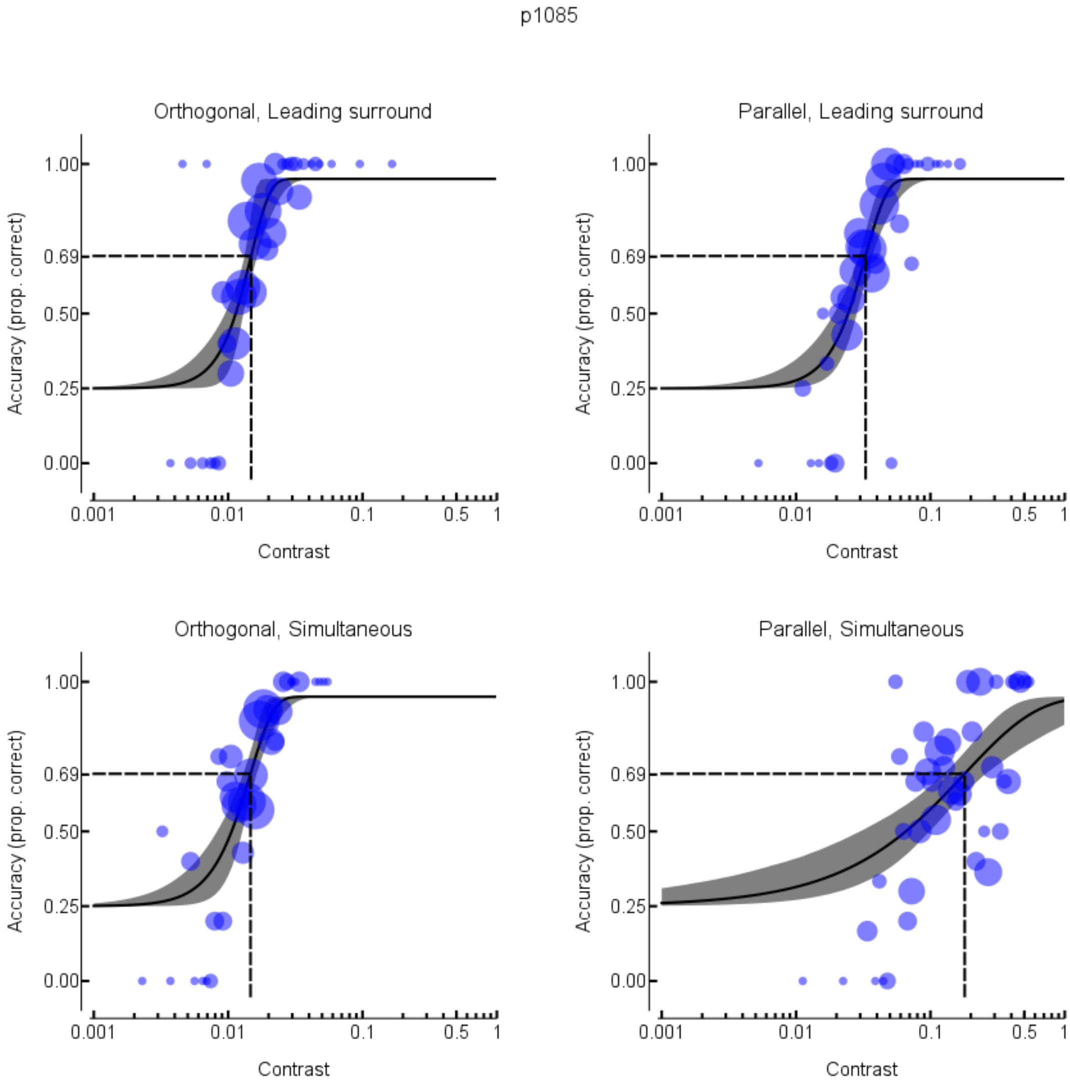
p1085
Orthogonal, Leading surround
Parallel, Leading surround
Orthogonal, Simultaneous
Parallel, Simultaneous
Accuracy (prop. correct)
1.00
0.69
0.50
0.25
0.00
0.001
0.01
0.1
0.5
1
Contrast

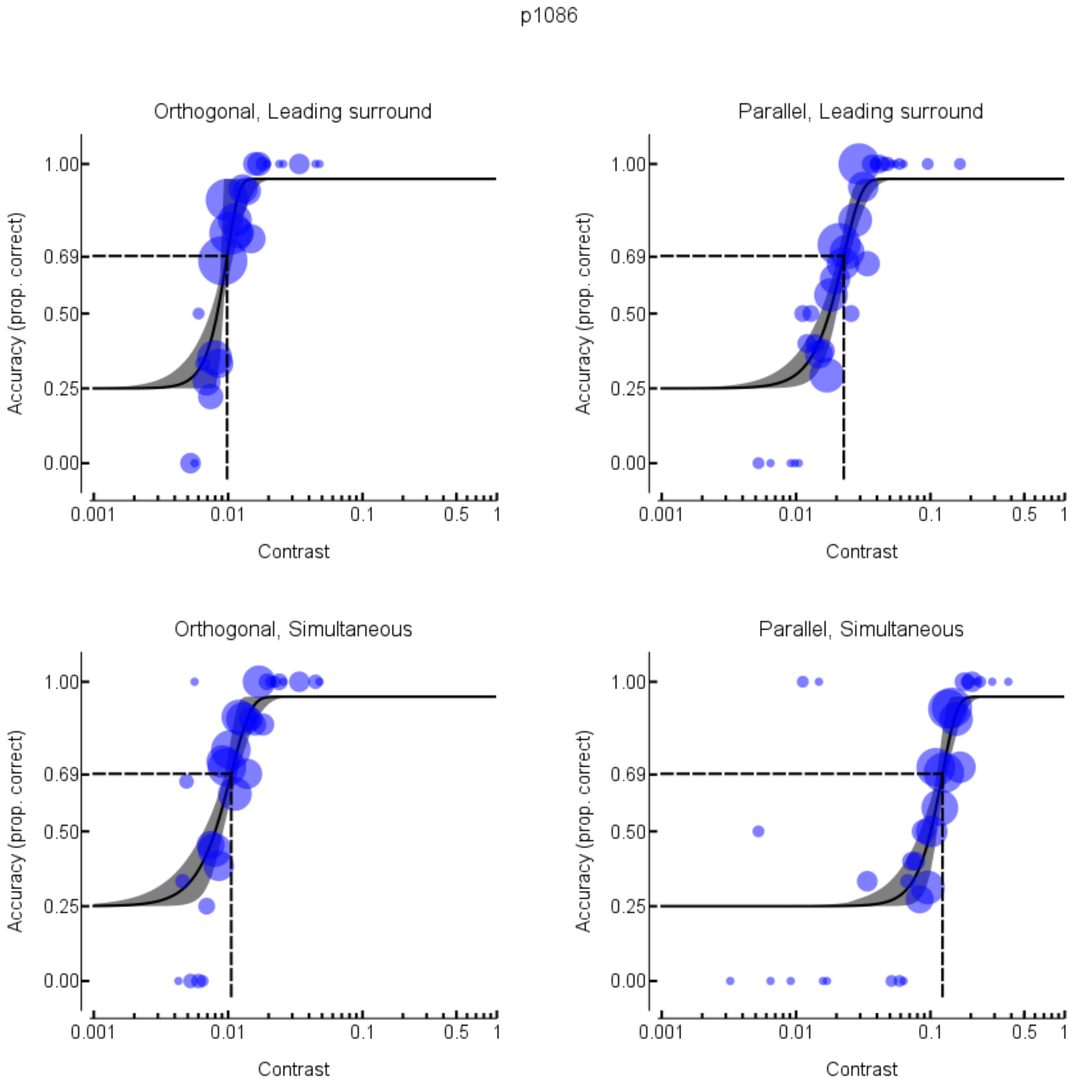

p1086
Orthogonal, Leading surround
Parallel, Leading surround
Orthogonal, Simultaneous
Parallel, Simultaneous
Accuracy (prop. correct)
1.00
0.69
0.50
0.25
0.00
0.001
0.01
0.1
0.5
1
Contrast

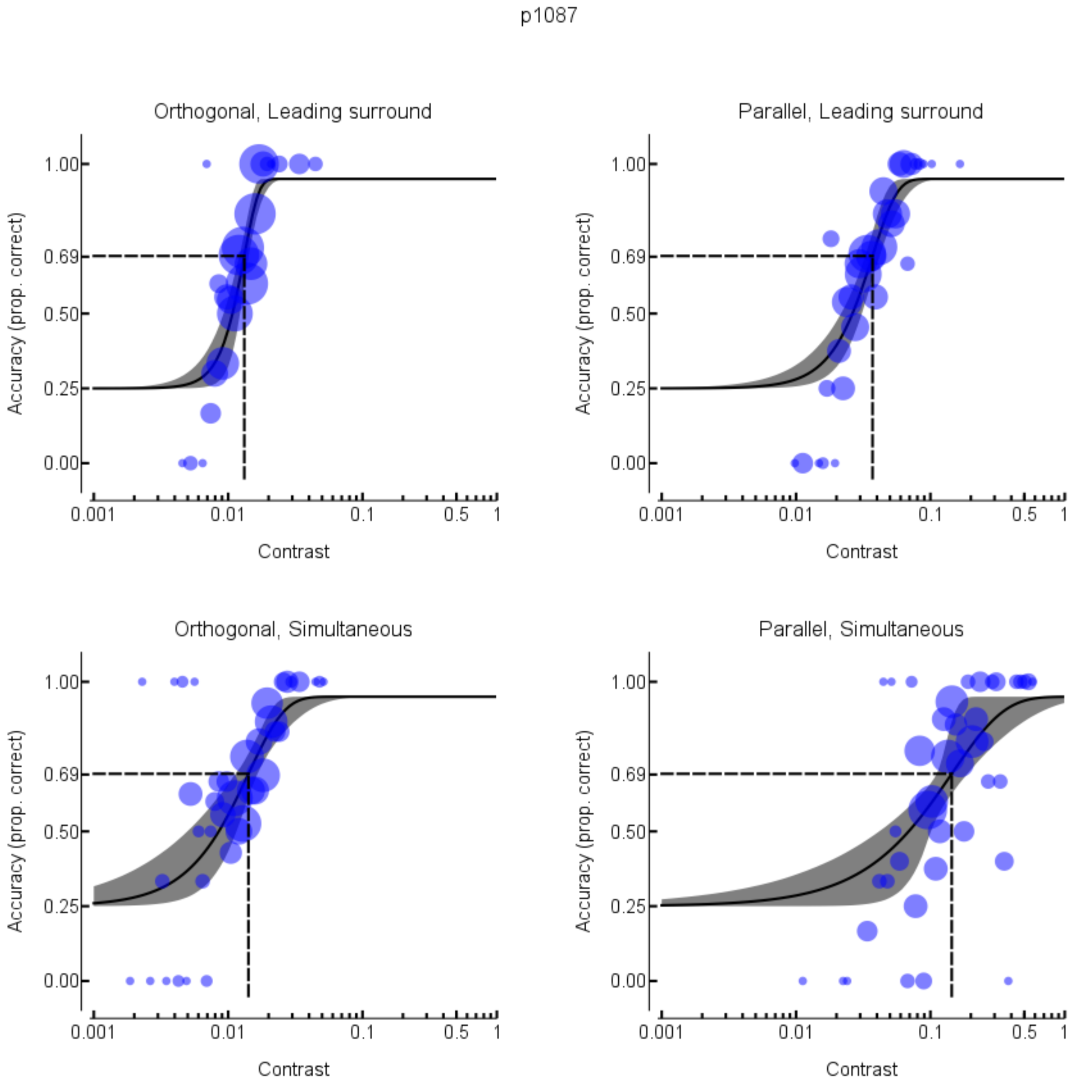

p1087
Orthogonal, Leading surround
Parallel, Leading surround
Orthogonal, Simultaneous
Parallel, Simultaneous
Accuracy (prop. correct)
1.00
0.69
0.50
0.25
0.00
0.001
0.01
0.1
0.5
1
Contrast

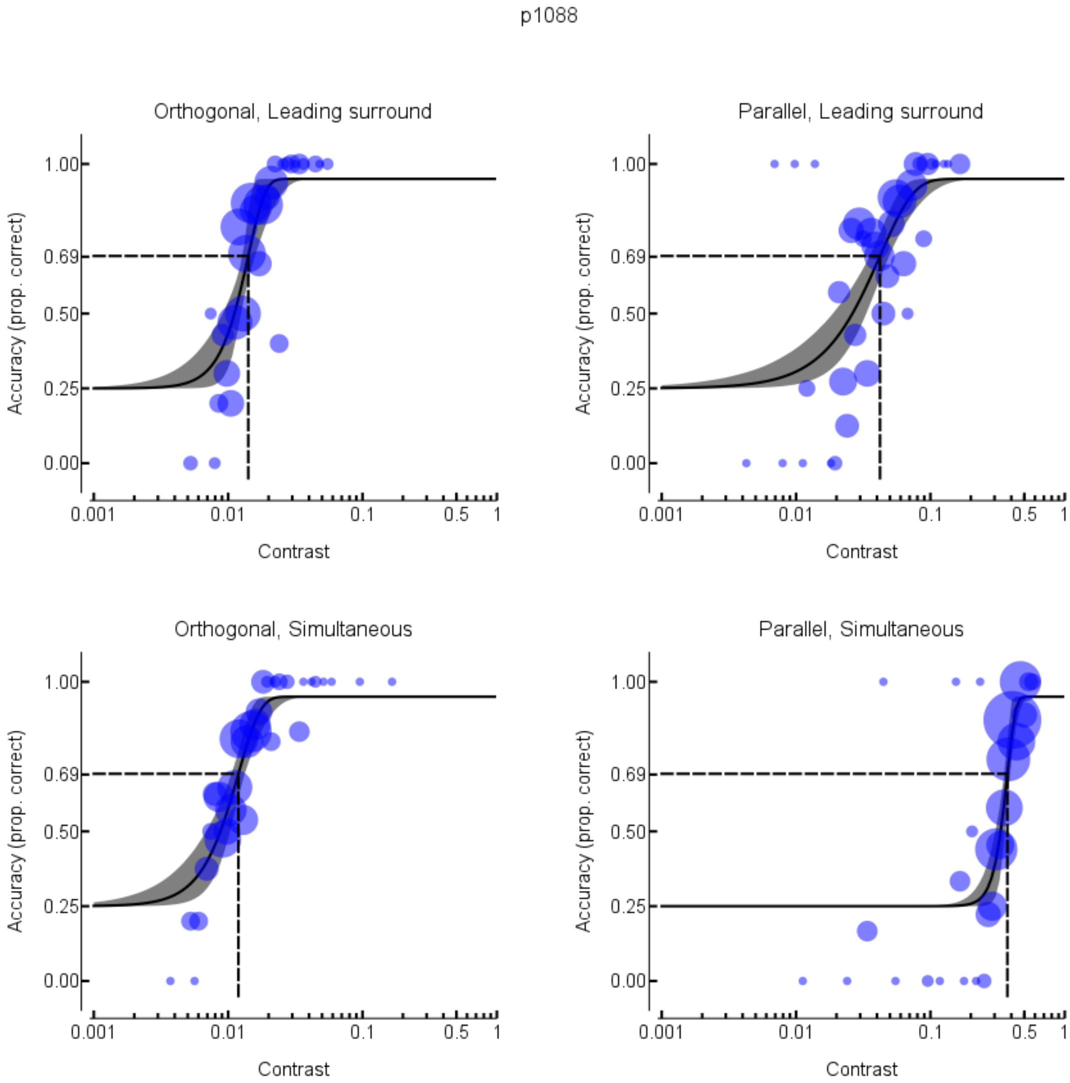
p1088
Orthogonal, Leading surround
Parallel, Leading surround
Orthogonal, Simultaneous
Parallel, Simultaneous
Accuracy (prop. correct)
1.00
0.69
0.50
0.25
0.00
0.001
0.01
0.1
0.5
1
Contrast

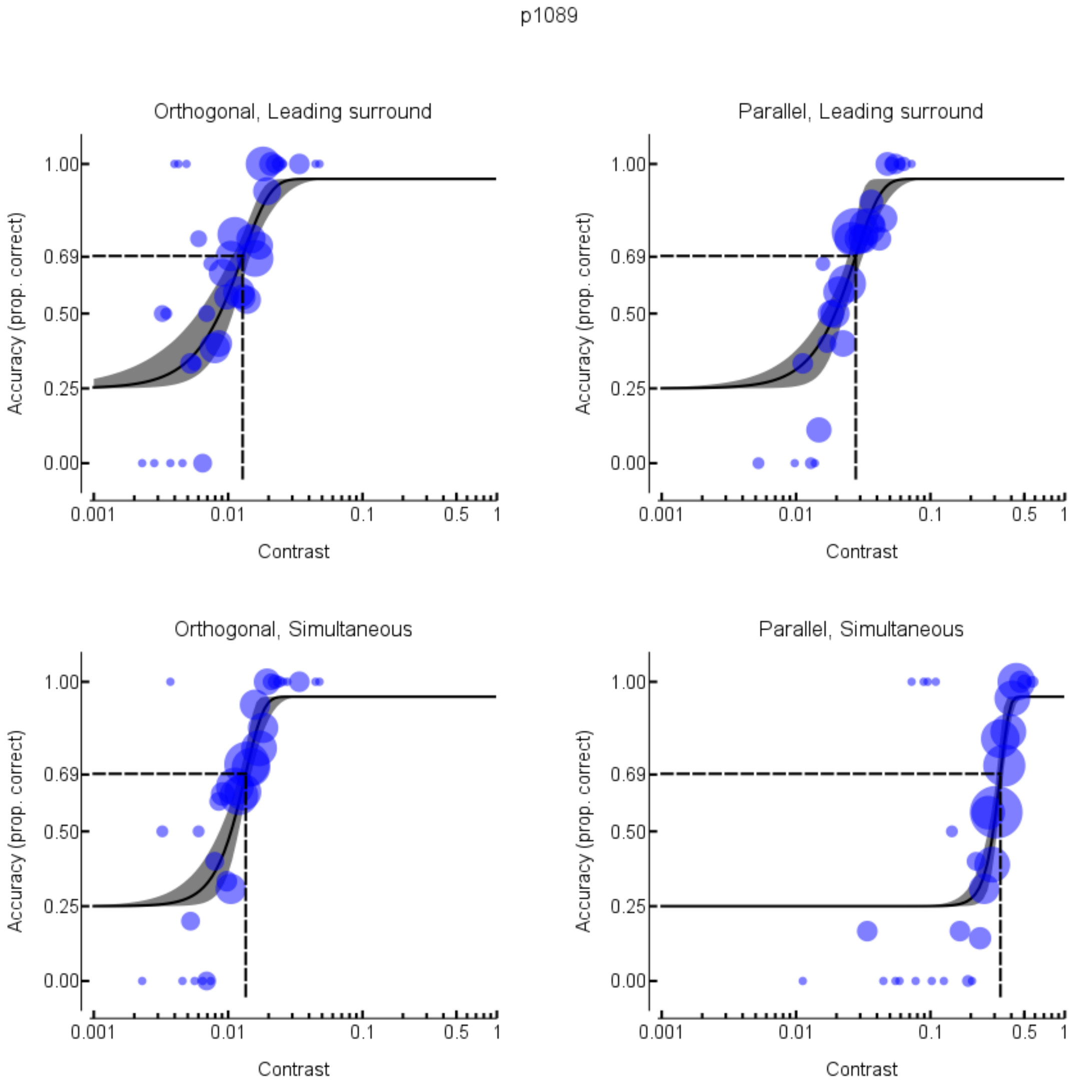
p1089
Orthogonal, Leading surround
Parallel, Leading surround
Orthogonal, Simultaneous
Parallel, Simultaneous
Accuracy (prop. correct)
1.00
0.69
0.50
0.25
0.00
Contrast
0.001
0.01
0.1
0.5
1

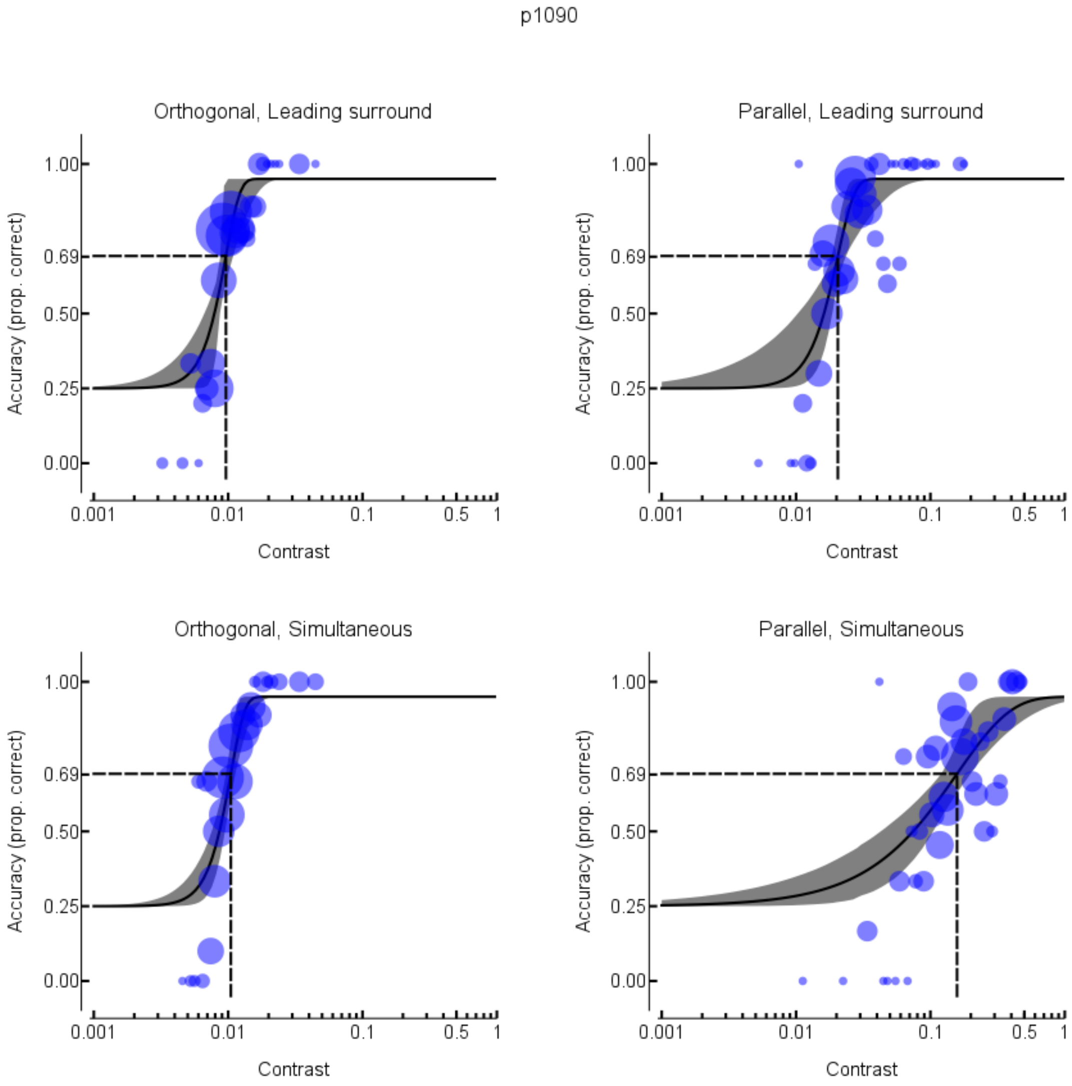
p1090
Orthogonal, Leading surround
Parallel, Leading surround
Orthogonal, Simultaneous
Parallel, Simultaneous
Accuracy (prop. correct)
1.00
0.69
0.50
0.25
0.00
0.001
0.01
0.1
0.5
1
Contrast

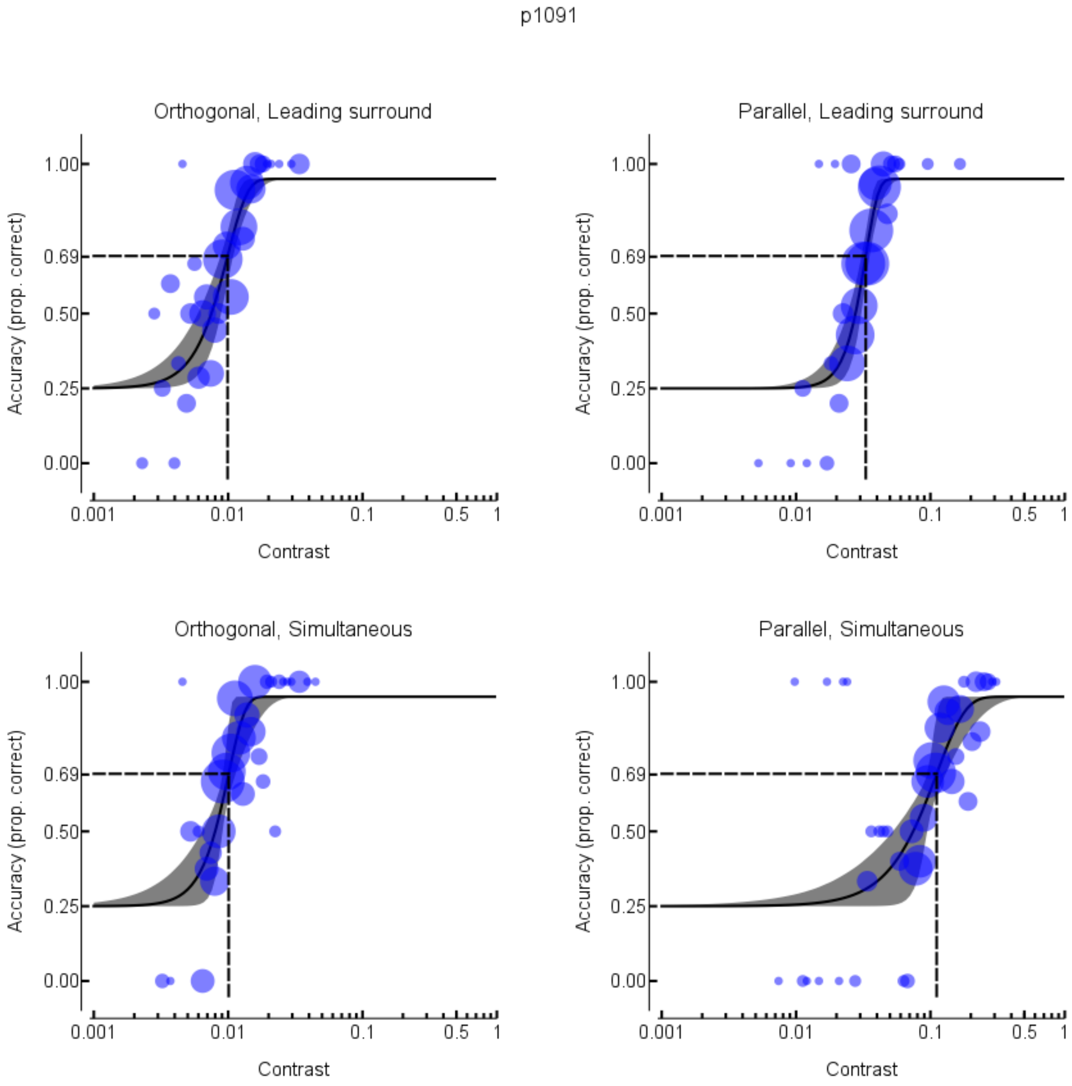
p1091
Orthogonal, Leading surround
Parallel, Leading surround
Orthogonal, Simultaneous
Parallel, Simultaneous
Accuracy (prop. correct)
1.00
0.69
0.50
0.25
0.00
Contrast
0.001
0.01
0.1
0.5
1

p1092

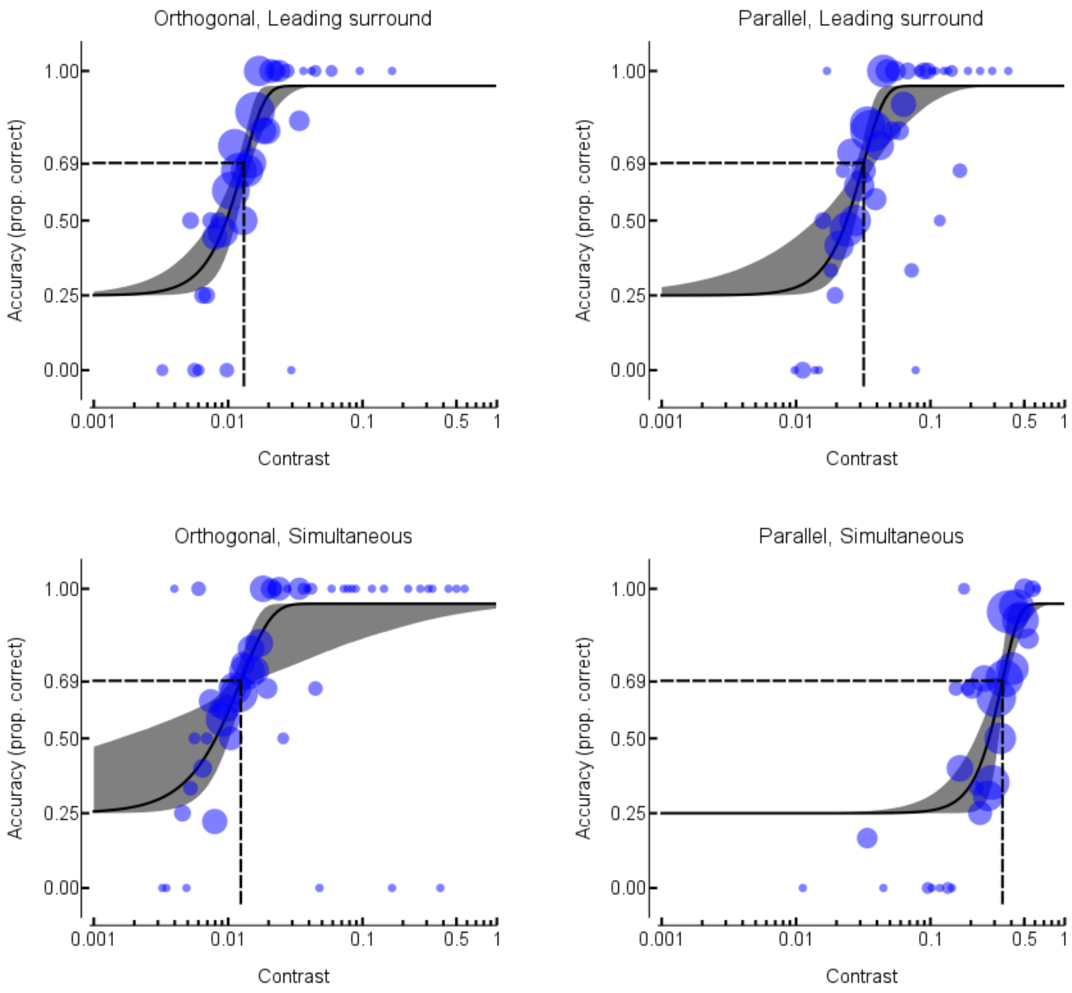
Orthogonal, Leading surround
Parallel, Leading surround
Orthogonal, Simultaneous
Parallel, Simultaneous
Accuracy (prop. correct)
1.00
0.69
0.50
0.25
0.00
Contrast
0.001
0.01
0.1
0.5
1

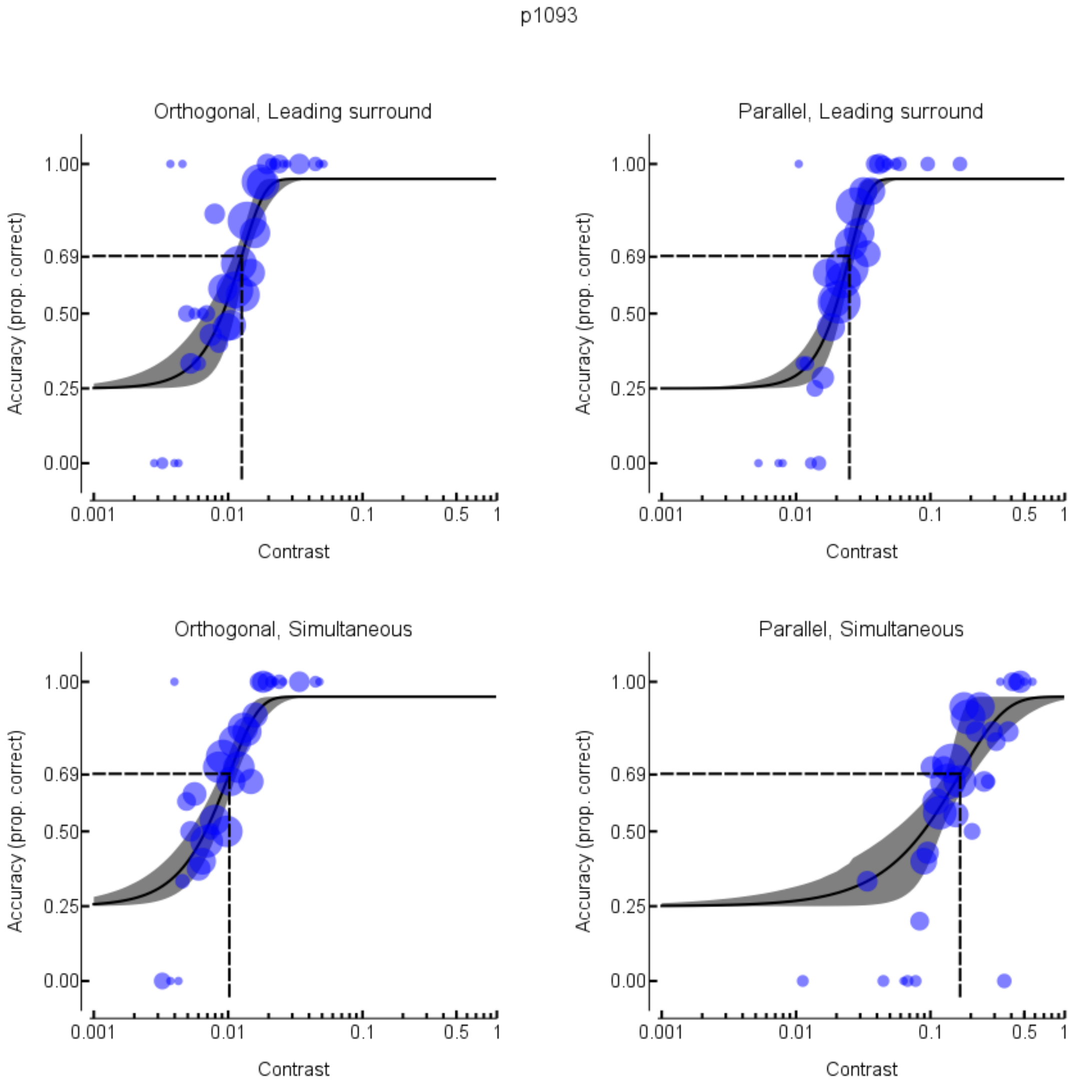
p1093
Orthogonal, Leading surround
Parallel, Leading surround
Orthogonal, Simultaneous
Parallel, Simultaneous
Accuracy (prop. correct)
1.00
0.69
0.50
0.25
0.00
0.001
0.01
0.1
0.5
1
Contrast

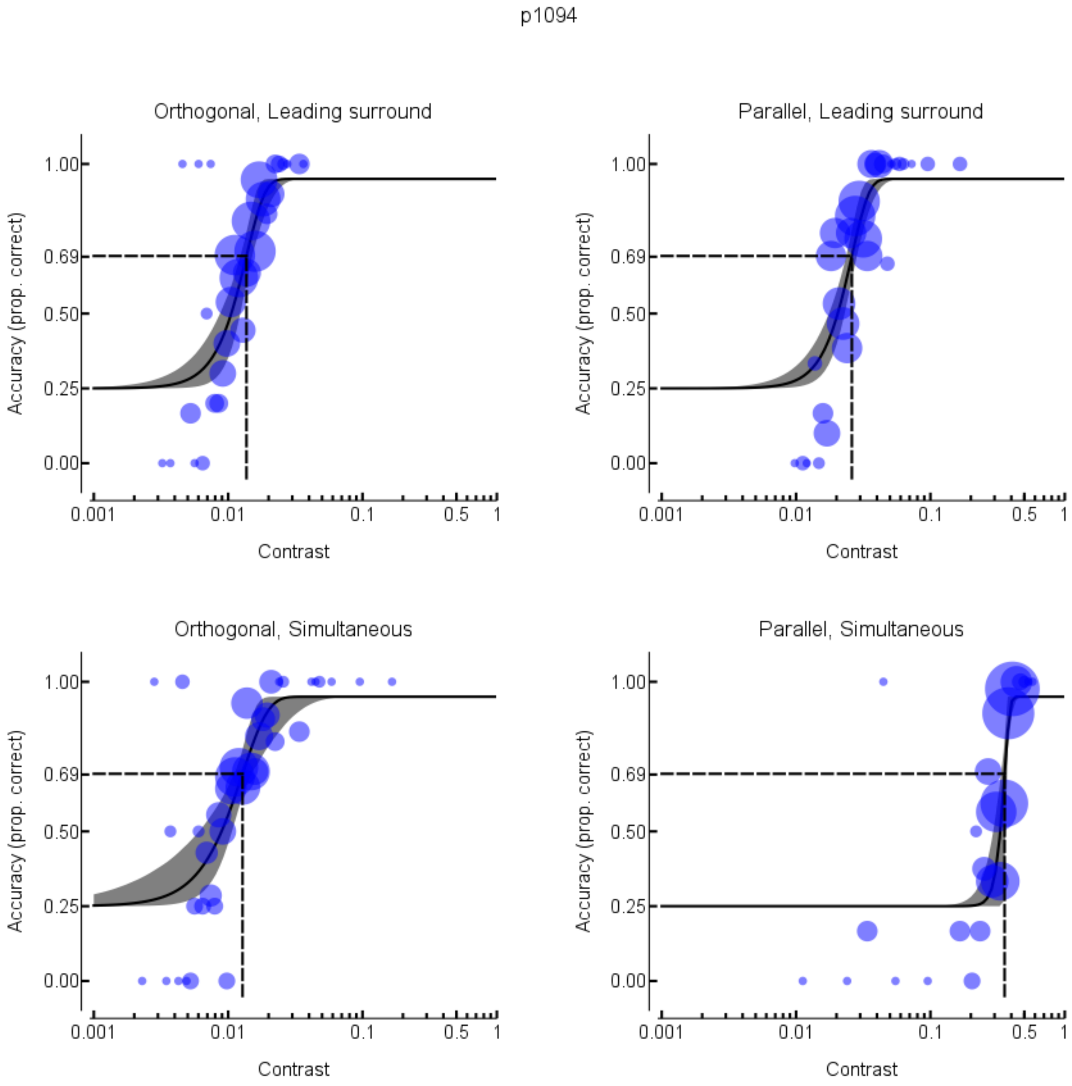
p1094
Orthogonal, Leading surround
Parallel, Leading surround
Orthogonal, Simultaneous
Parallel, Simultaneous
Accuracy (prop. correct)
1.00
0.69
0.50
0.25
0.00
0.001
0.01
0.1
0.5
1
Contrast

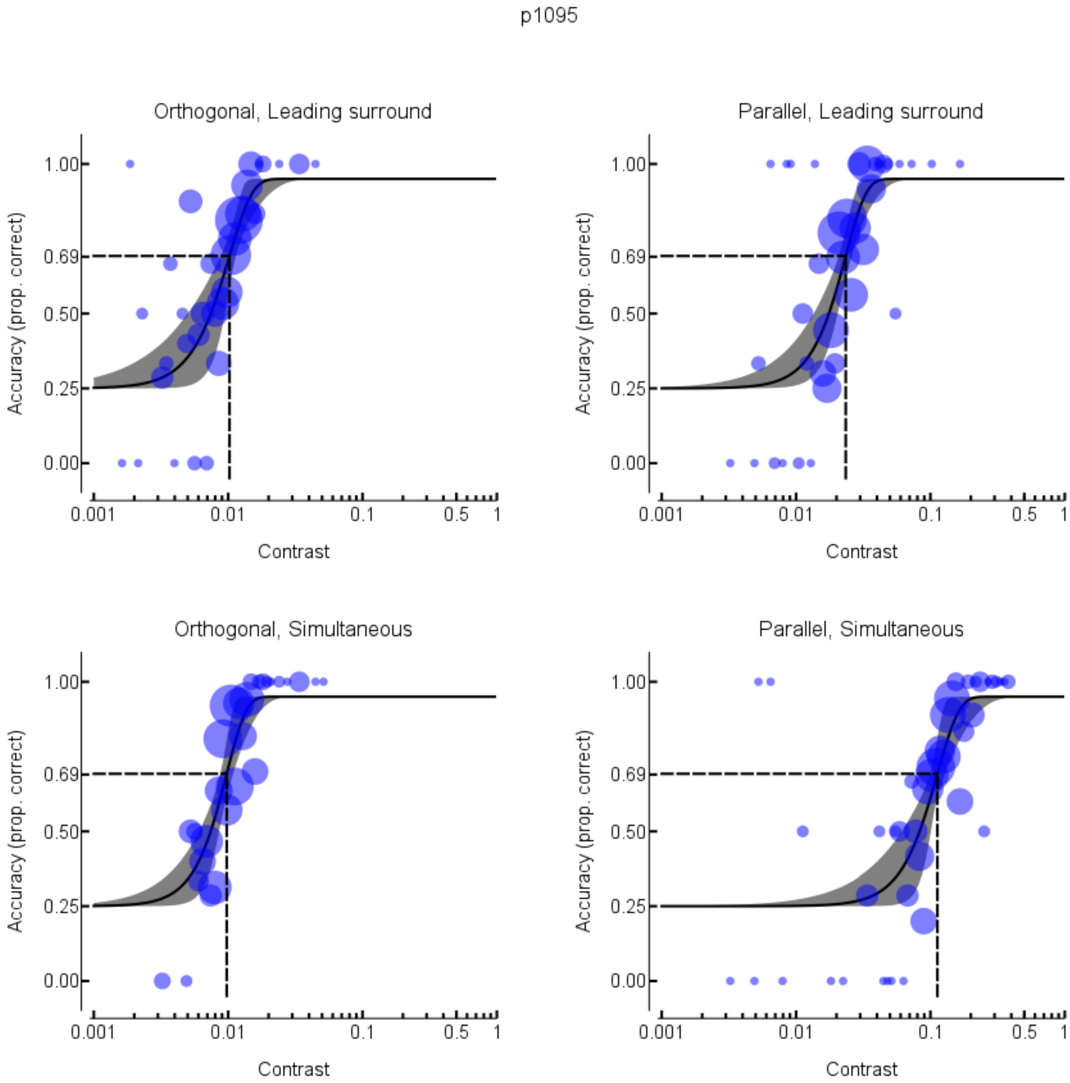
p1095
Orthogonal, Leading surround
Parallel, Leading surround
Orthogonal, Simultaneous
Parallel, Simultaneous
Accuracy (prop. correct)
1.00
0.69
0.50
0.25
0.00
0.001
0.01
0.1
0.5
1
Contrast

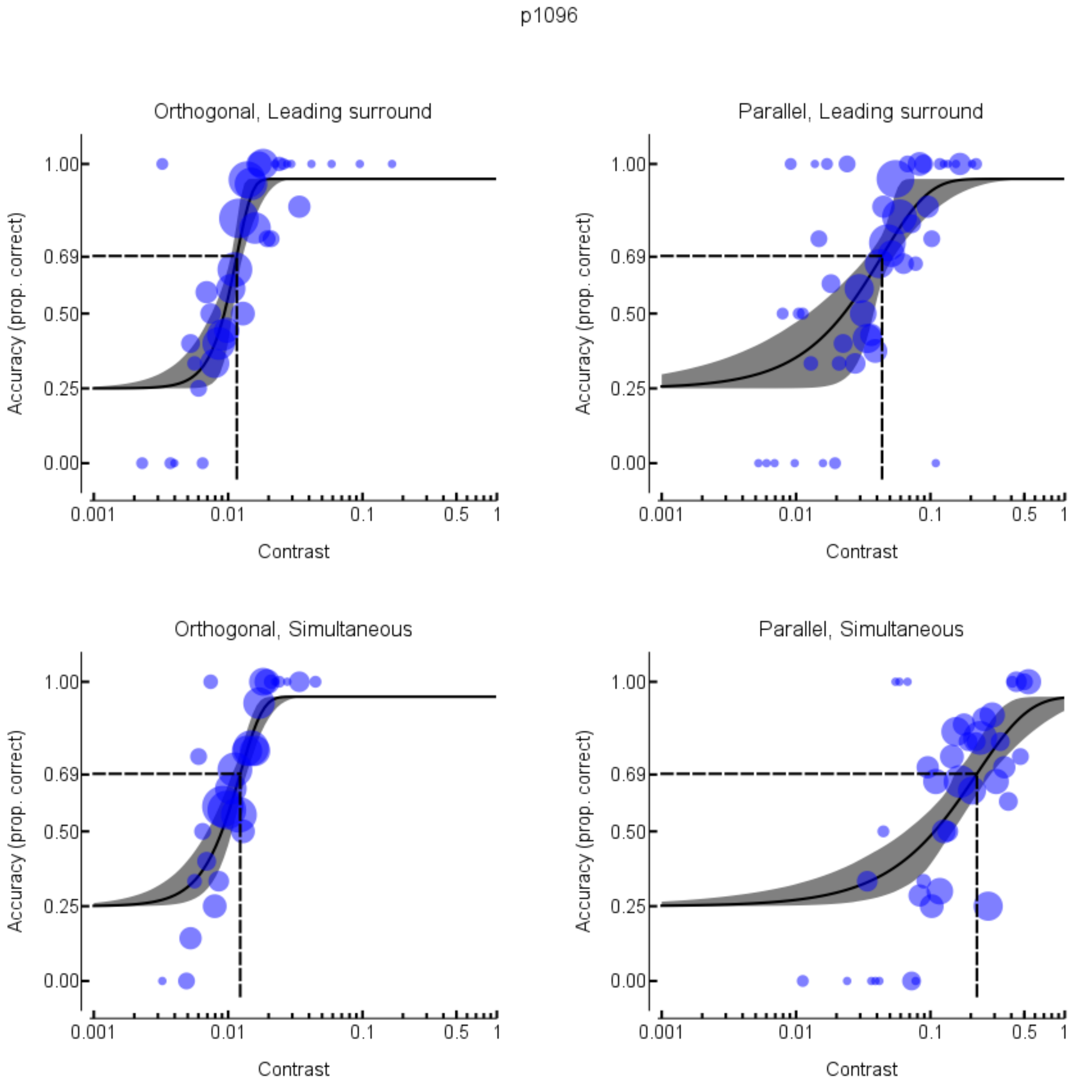
p1096
Orthogonal, Leading surround
Parallel, Leading surround
Orthogonal, Simultaneous
Parallel, Simultaneous
Accuracy (prop. correct)
1.00
0.69
0.50
0.25
0.00
0.001
0.01
0.1
0.5
1
Contrast

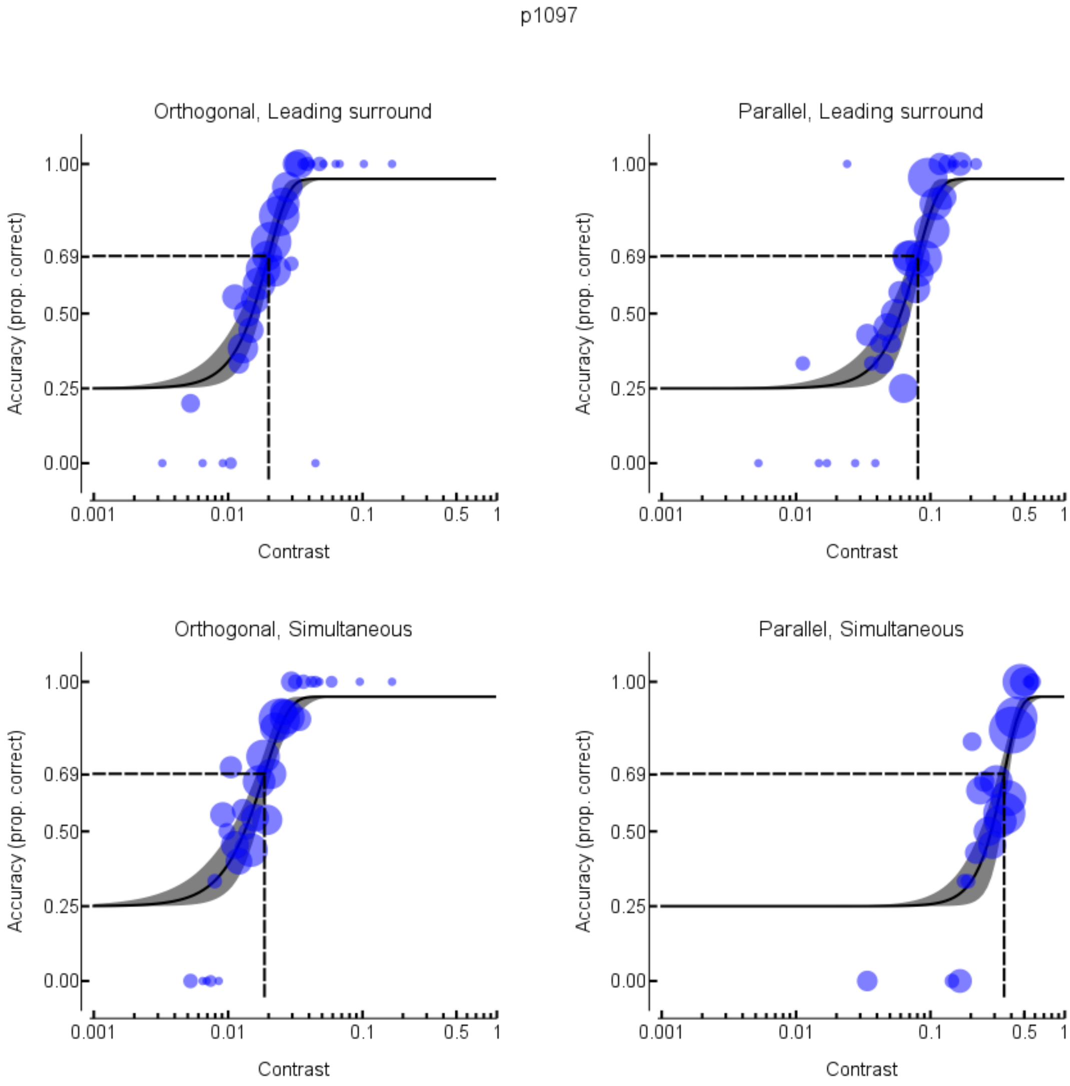
p1097
Orthogonal, Leading surround
Parallel, Leading surround
Orthogonal, Simultaneous
Parallel, Simultaneous
Accuracy (prop. correct)
1.00
0.69
0.50
0.25
0.00
Contrast
0.001
0.01
0.1
0.5
1

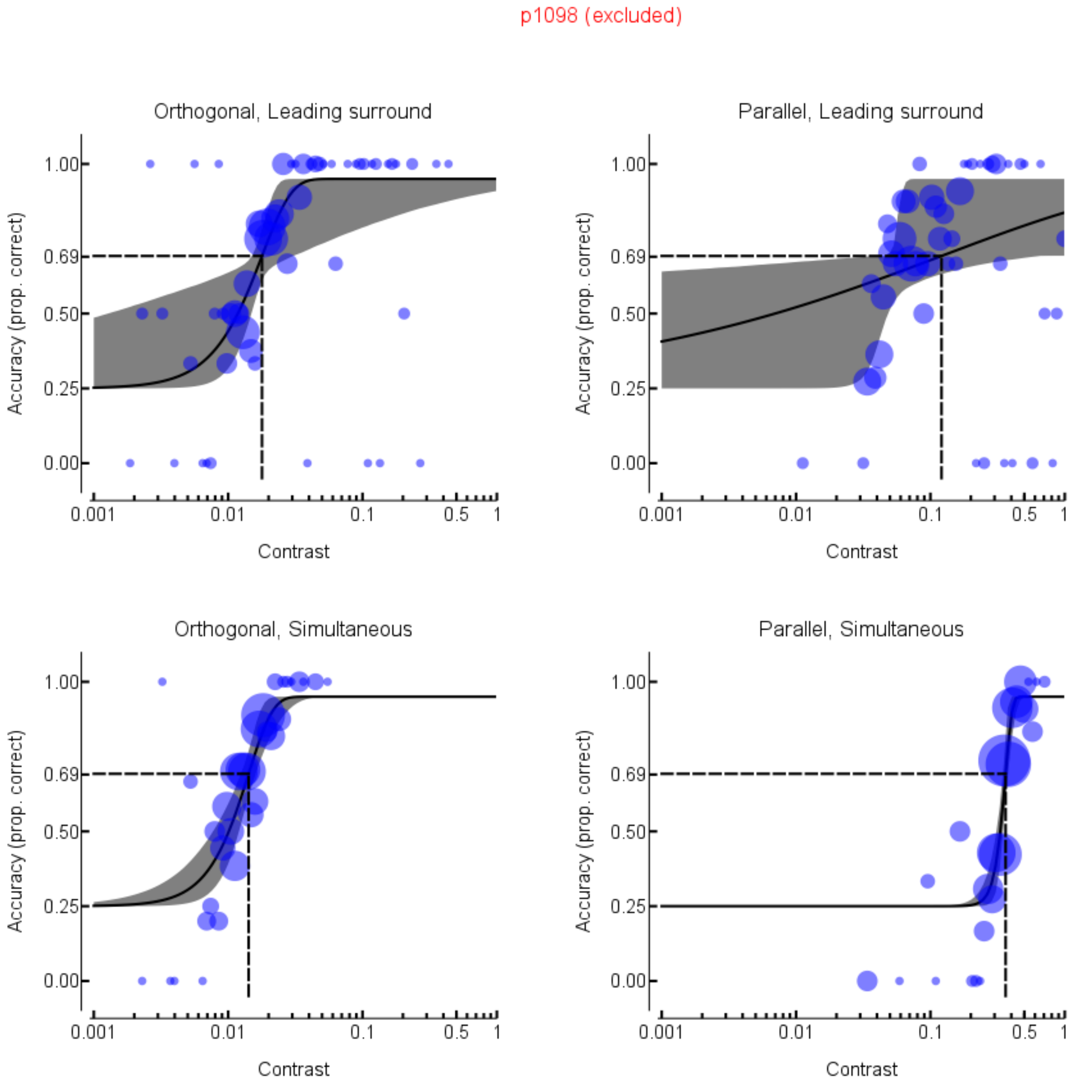
p1098 (excluded)
Orthogonal, Leading surround
Parallel, Leading surround
Orthogonal, Simultaneous
Parallel, Simultaneous
Accuracy (prop. correct)
1.00
0.69
0.50
0.25
0.00
0.001
0.01
0.1
0.5
1
Contrast

p1099

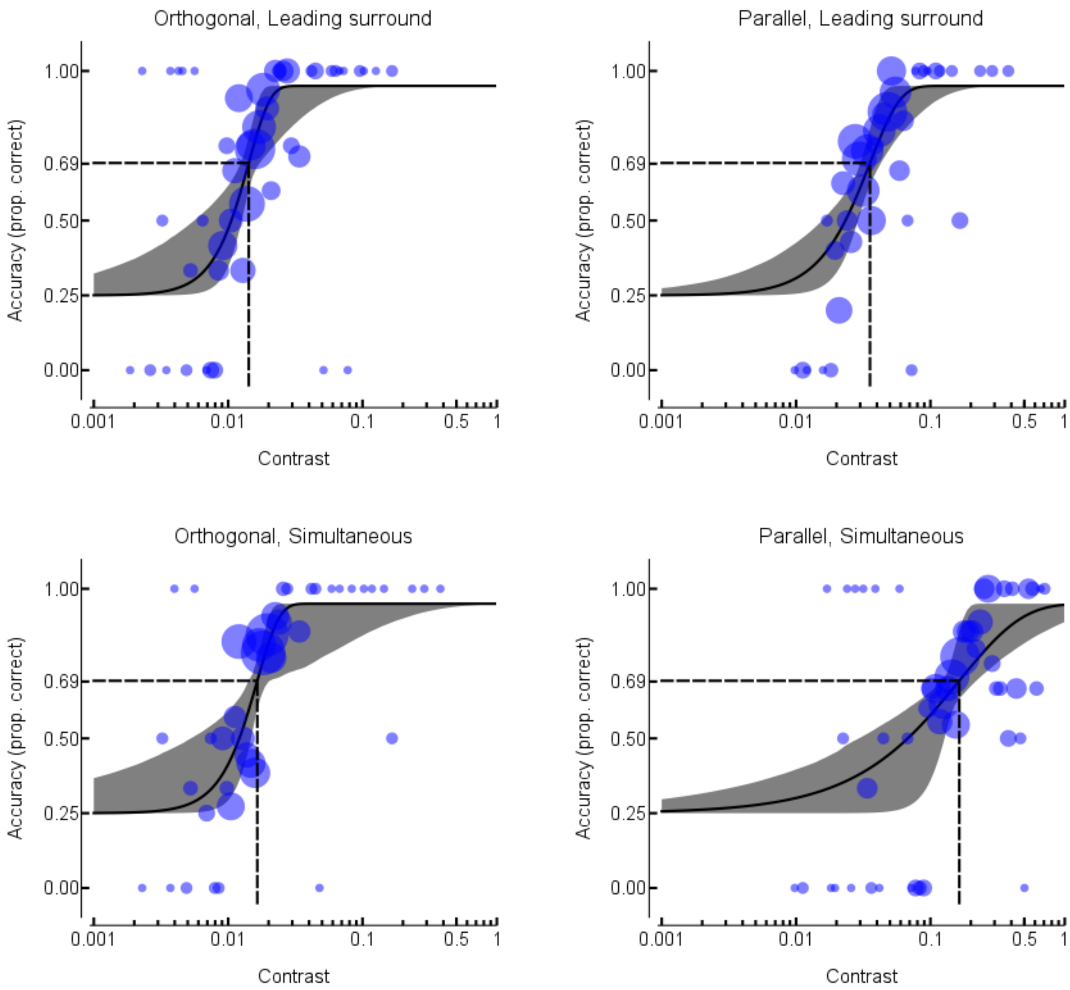
Orthogonal, Leading surround
Parallel, Leading surround
Orthogonal, Simultaneous
Parallel, Simultaneous
Accuracy (prop. correct)
1.00
0.69
0.50
0.25
0.00
0.001
0.01
0.1
0.5
1
Contrast

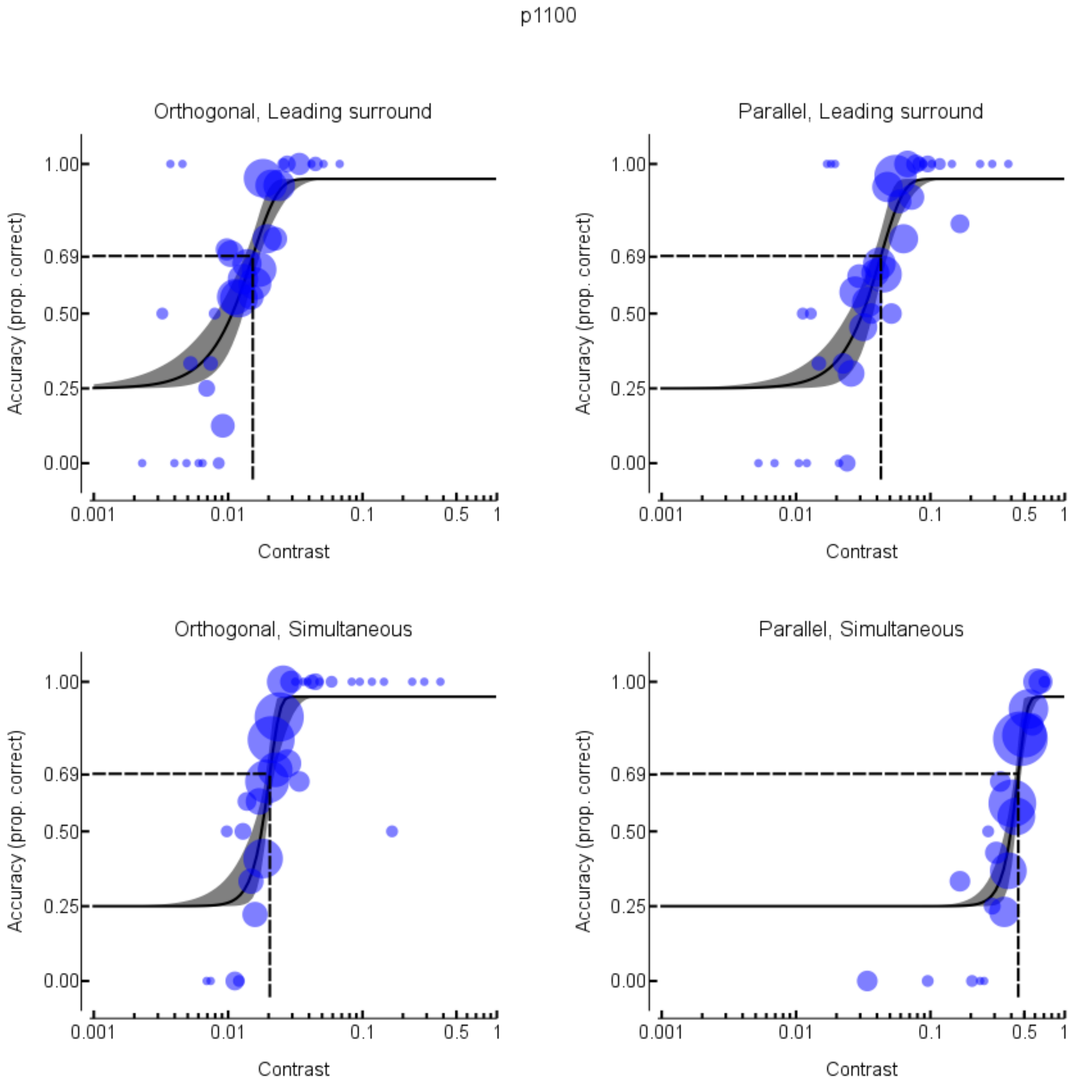
p1100
Orthogonal, Leading surround
Parallel, Leading surround
Orthogonal, Simultaneous
Parallel, Simultaneous
Accuracy (prop. correct)
1.00
0.69
0.50
0.25
0.00
0.001
0.01
0.1
0.5
1
Contrast

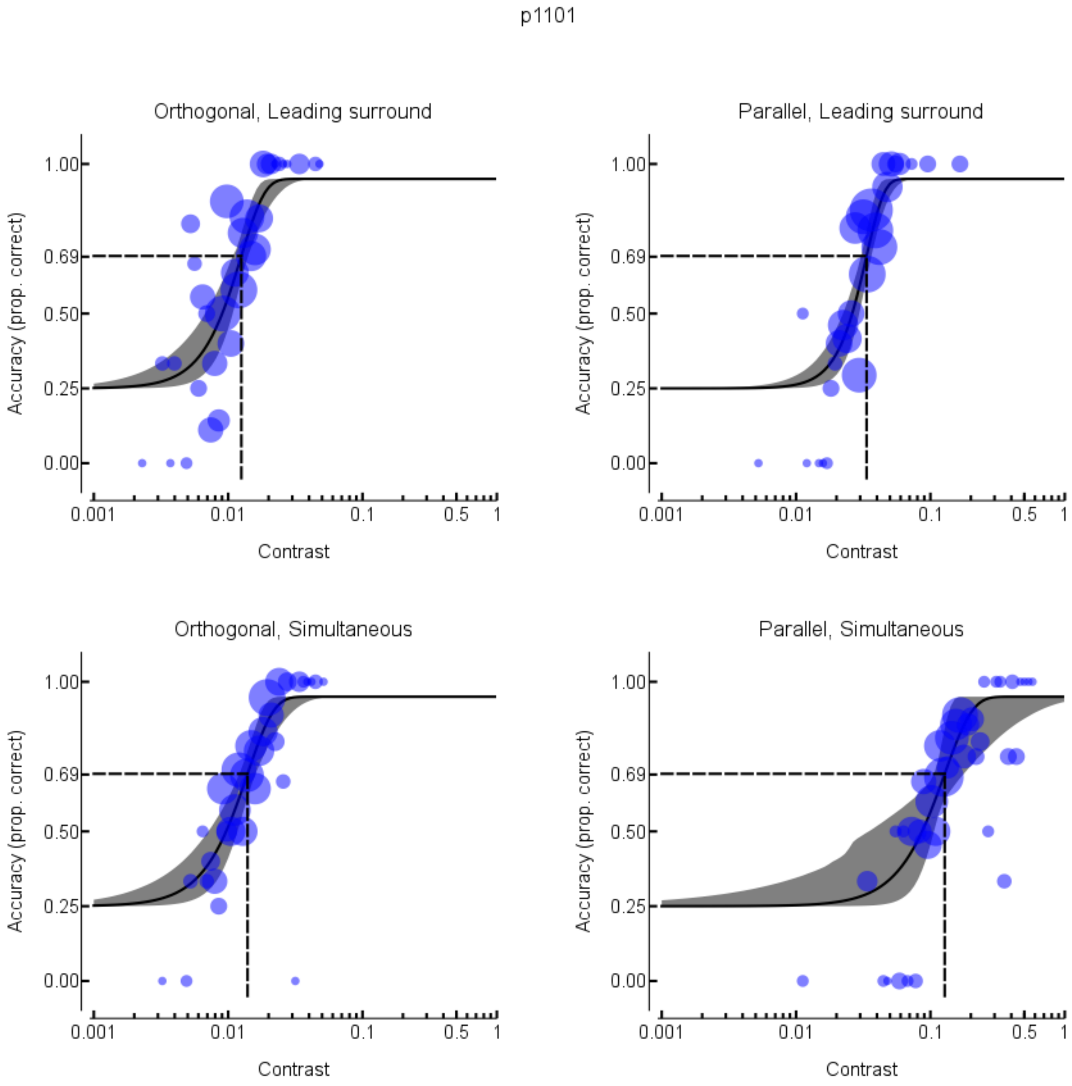
p1101
Orthogonal, Leading surround
Parallel, Leading surround
Orthogonal, Simultaneous
Parallel, Simultaneous
Accuracy (prop. correct)
1.00
0.69
0.50
0.25
0.00
Contrast
0.001
0.01
0.1
0.5
1
