# Supplementary material for: No apparent influence of psychometrically-defined schizotypy on orientation-dependent contextual modulation of visual contrast detection
Source: PeerJ. 2017 Jan 24;5:e2921. doi: 10.7717/peerj.2921 (PMC5267566; doi:10.7717/peerj.2921)
Supplement: Figure S2 — Panels show the different subscales of the O-LIFE questionnaire, and boxplots show the 25th, 50th, 75th, and 90th percentiles for the “<22” age group norms reported by Mason & Claridge (1996) and for those obtained in the current study, separated by gender. [file peerj-05-2921-s002.pdf]

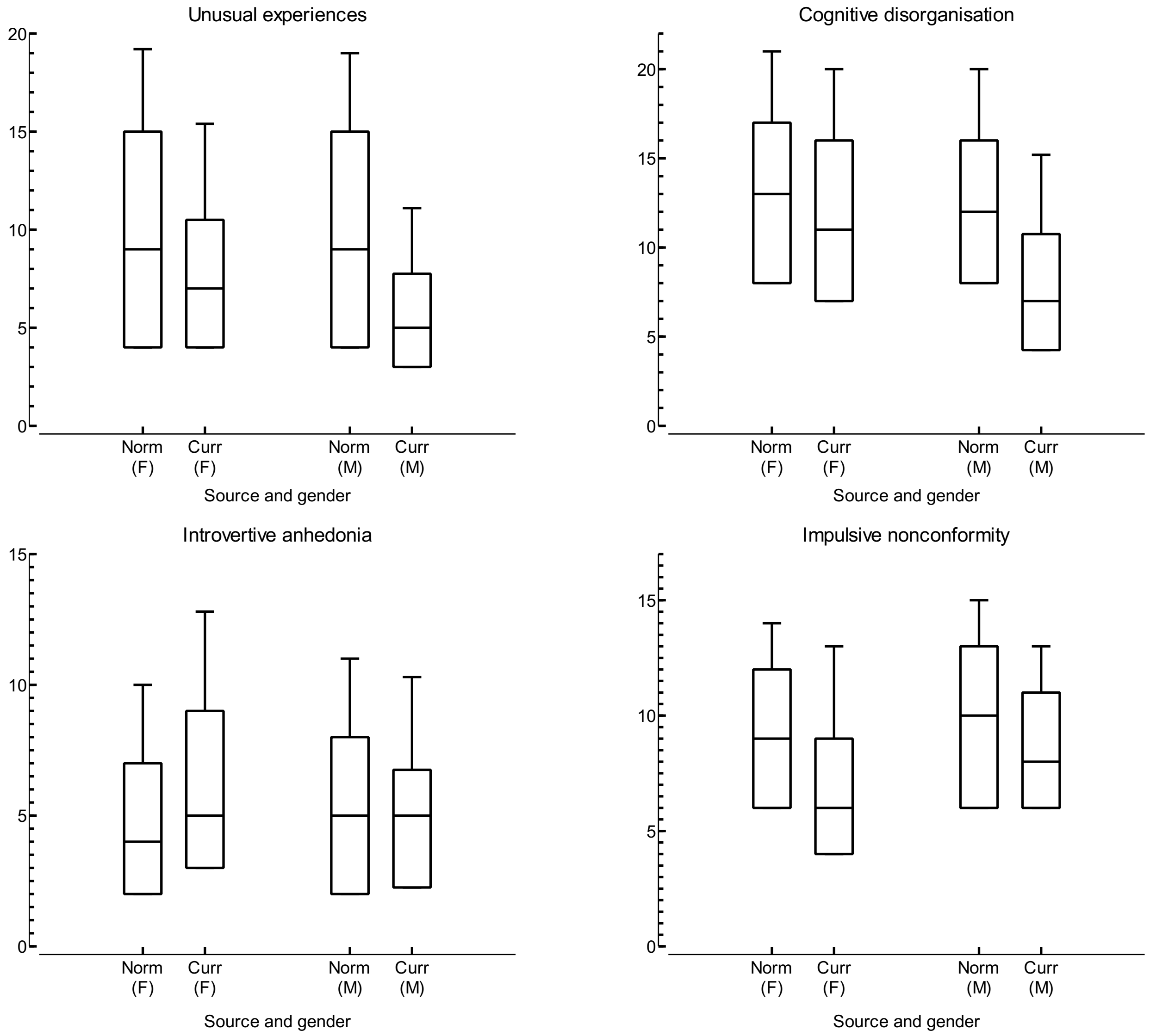

Fig. S 2 Comparison of the O-LIFE scores reported by Mason & Claridge (1996) and the current study. Panels show the different subscales of the O-LIFE questionnaire, and boxplots show the 25th, 50th, 75th, and 90th percentiles for the "< 22" age group norms reported by Mason & Claridge (1996) and for those obtained in the current study, separated by gender.
