# Supplementary material for: No apparent influence of psychometrically-defined schizotypy on orientation-dependent contextual modulation of visual contrast detection
Source: PeerJ. 2017 Jan 24;5:e2921. doi: 10.7717/peerj.2921 (PMC5267566; doi:10.7717/peerj.2921)
Supplement: Figure S3 — Correlation coefficients and p values refer to the Pearson correlation on the ranked values (Spearman’s correlation). [file peerj-05-2921-s003.pdf]

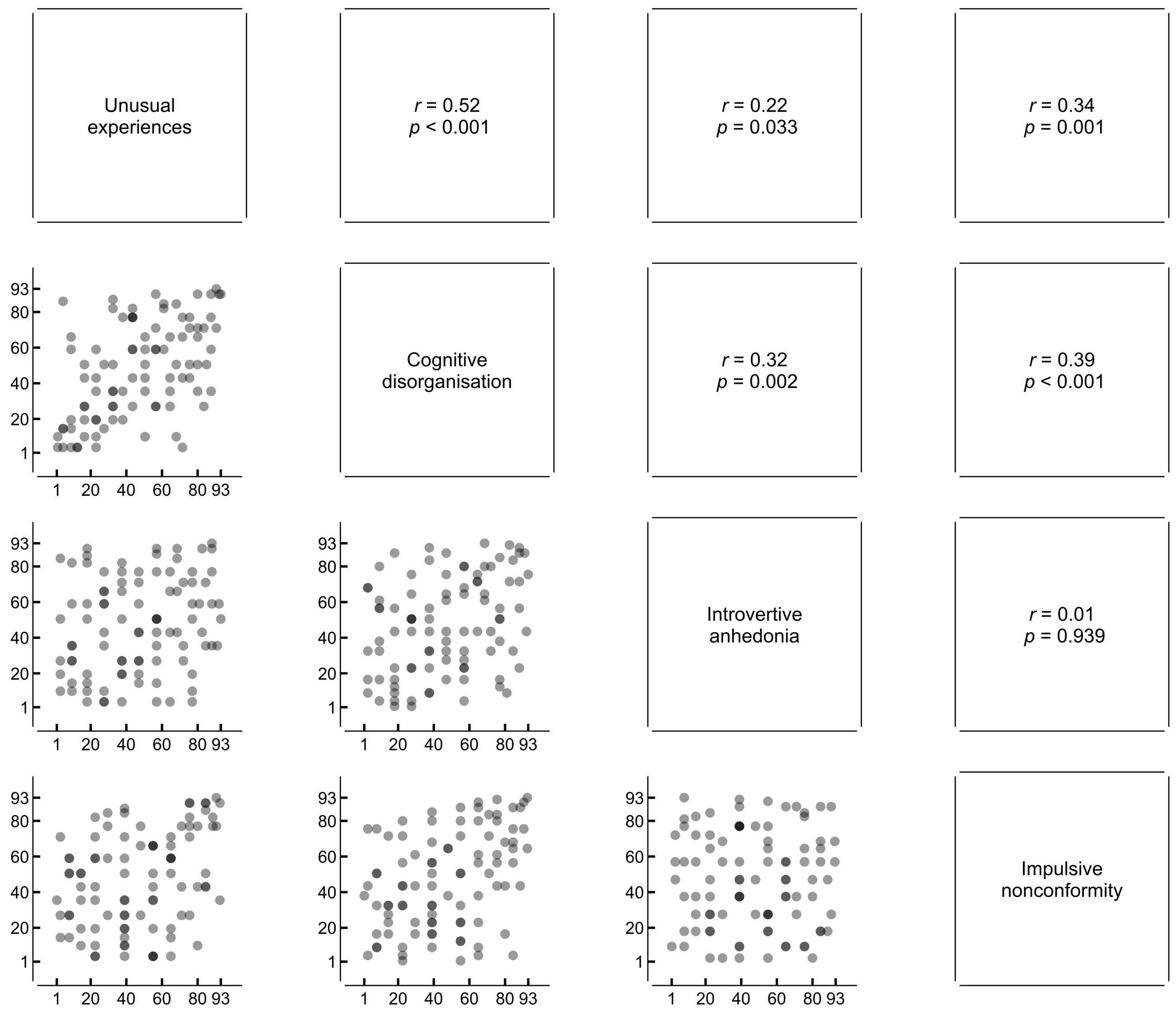

Fig. S 3 Pairwise correlations between the ranked scores on the O-LIFE subscales. Correlation coefficients and *p* values refer to the Pearson correlation on the ranked values (Spearman's correlation).
