# Supplementary material for: No apparent influence of psychometrically-defined schizotypy on orientation-dependent contextual modulation of visual contrast detection
Source: PeerJ. 2017 Jan 24;5:e2921. doi: 10.7717/peerj.2921 (PMC5267566; doi:10.7717/peerj.2921)
Supplement: Figure S4 — Each point shows a single participant’s O-LIFE subscale score and orientation-dependent context effect (the difference between the contrast detection threshold with a parallel surround and that with an orthogonal surround, both during simultaneous presentation). [file peerj-05-2921-s004.pdf]

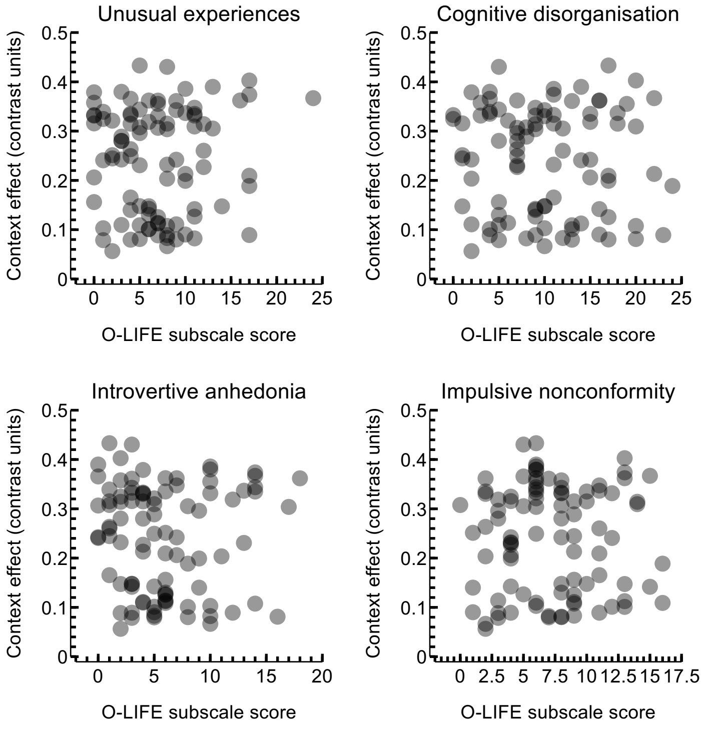

Fig. S 4 Comparison of O-LIFE score and the magnitude of the orientation-dependent effect of context during simultaneous presentation. Each point shows a single participant's O-LIFE subscale score and orientation-dependent context effect (the difference between the contrast detection threshold with a parallel surround and that with an orthogonal surround, both during simultaneous presentation).
