# Supplementary material for: No apparent influence of psychometrically-defined schizotypy on orientation-dependent contextual modulation of visual contrast detection
Source: PeerJ. 2017 Jan 24;5:e2921. doi: 10.7717/peerj.2921 (PMC5267566; doi:10.7717/peerj.2921)
Supplement: Figure S5 [file peerj-05-2921-s005.pdf]

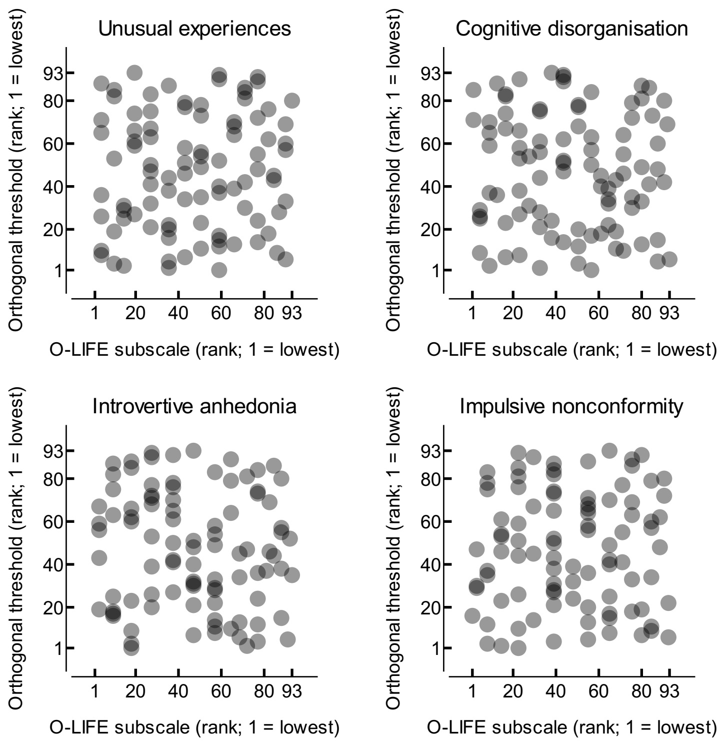

Fig. S 5 Comparison of ranked schizotypy score and the ranked contrast detection threshold with orthogonal context during simultaneous presentation.
