# Supplementary material for: No apparent influence of psychometrically-defined schizotypy on orientation-dependent contextual modulation of visual contrast detection
Source: PeerJ. 2017 Jan 24;5:e2921. doi: 10.7717/peerj.2921 (PMC5267566; doi:10.7717/peerj.2921)
Supplement: Figure S6 [file peerj-05-2921-s006.pdf]

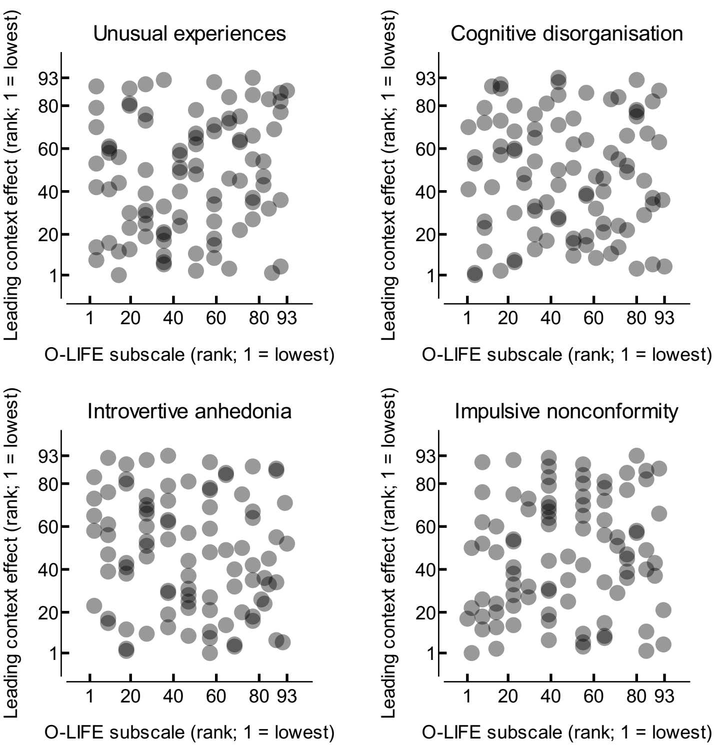

Fig. S 6 Comparison of ranked schizotypy score and the ranked magnitude of the difference between contrast detection thresholds with parallel and orthogonal context for the 'leading surround' condition.

.
