# Supplementary material for: No apparent influence of psychometrically-defined schizotypy on orientation-dependent contextual modulation of visual contrast detection
Source: PeerJ. 2017 Jan 24;5:e2921. doi: 10.7717/peerj.2921 (PMC5267566; doi:10.7717/peerj.2921)
Supplement: Figure S7 — Boxplots show the difference between the contrast detection thresholds for parallel and orthogonal contexts for simultaneous presentation, separately for females and males. [file peerj-05-2921-s007.pdf]

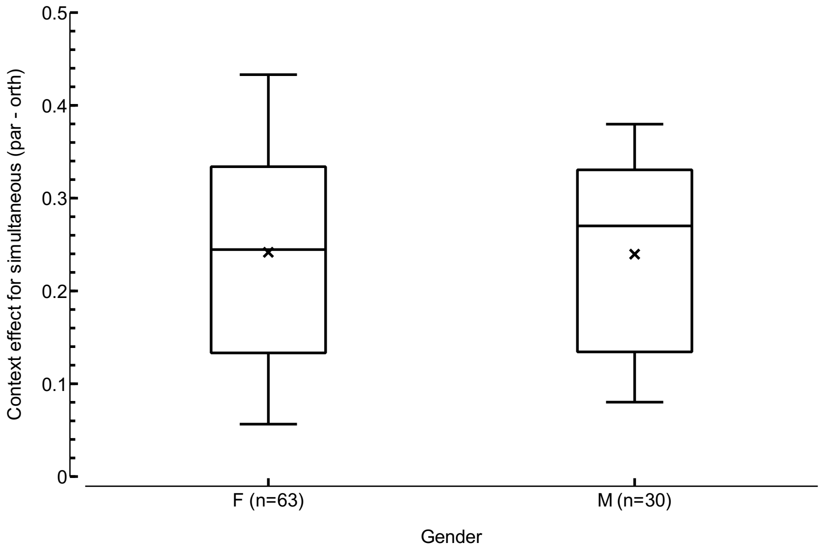

Fig. S 7 Distribution of orientation-dependent contextual modulation effects for females and males. Boxplots show the difference between the contrast detection thresholds for parallel and orthogonal contexts for simultaneous presentation, separately for females and males.
