# Supplementary material for: No apparent influence of psychometrically-defined schizotypy on orientation-dependent contextual modulation of visual contrast detection
Source: PeerJ. 2017 Jan 24;5:e2921. doi: 10.7717/peerj.2921 (PMC5267566; doi:10.7717/peerj.2921)
Supplement: Figure S8 — Boxplots show the difference between the contrast detection thresholds for parallel and orthogonal contexts for simultaneous presentation, separately for the two physical testing booths that were used in this study. [file peerj-05-2921-s008.pdf]

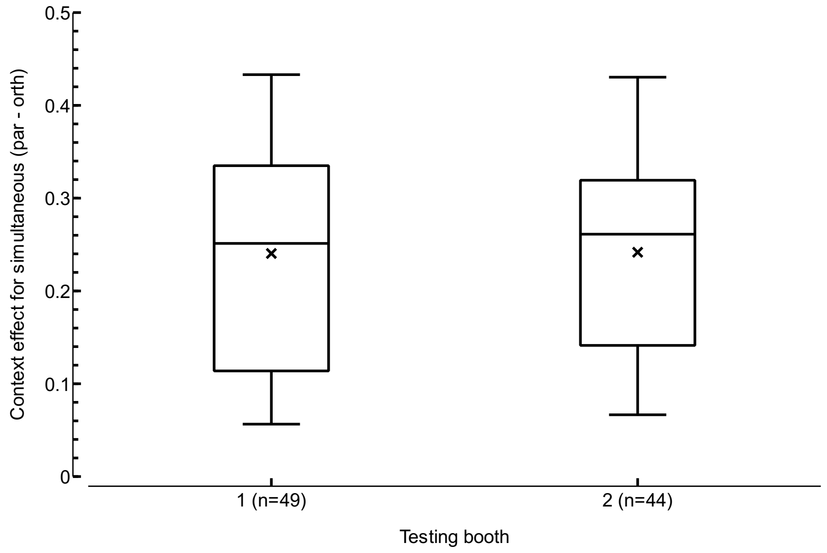

Fig. S 8 Distribution of orientation-dependent contextual modulation effects for the two testing booths. Boxplots show the difference between the contrast detection thresholds for parallel and orthogonal contexts for simultaneous presentation, separately for the two physical testing booths that were used in this study.
