# Supplementary material for: No apparent influence of psychometrically-defined schizotypy on orientation-dependent contextual modulation of visual contrast detection
Source: PeerJ. 2017 Jan 24;5:e2921. doi: 10.7717/peerj.2921 (PMC5267566; doi:10.7717/peerj.2921)
Supplement: Table S1 [file peerj-05-2921-s009.pdf]

Table S 1 Distribution of participant ages, after application of exclusion criteria.

Age	Count
18	36
19	35
20	10
21	3
23	4
25	2
28	1
30	1
31	1
