# Supplementary material for: No apparent influence of psychometrically-defined schizotypy on orientation-dependent contextual modulation of visual contrast detection
Source: PeerJ. 2017 Jan 24;5:e2921. doi: 10.7717/peerj.2921 (PMC5267566; doi:10.7717/peerj.2921)
Supplement: Article S1 [file peerj-05-2921-s010.pdf]

Re-analysis of previous orientation-dependent surround suppression schizophrenia studies

Supplementary material for:

Mannion, D.J., Donkin, C., & Whitford, T.J. No apparent influence of psychometrically-defined schizotypy on orientation-dependent contextual modulation of visual contrast detection, *PeerJ*.

Yoon et al. (2009)

Key results of interest

Yoon et al. (2009) use a two-way mixed design, with group (control, patient) as a between-subjects factor and surround stimulus (none [NS], orthogonal [OS], parallel [PS]) as a within-subjects factor. We are particularly interested in the analyses in which the orthogonal and parallel conditions are expressed as a ratio of the no-surround condition ('suppression indices').

First, to check that we have interpreted the raw data correctly, we reproduce their analyses[1] For the OS/NS condition, controls had a lower suppression index ($M = 1.26$) than patients ($M = 1.49$). For the PS/NS condition, controls had a higher suppression index ($M = 2.74$) than patients ($M = 2.31$). These values appear to be in agreement with Fig. 2 in Yoon et al. (2009).

Check of ratio assumptions

The key assumptions of the use of a ratio to adjust the numerator to control for a particular variable (denominator) are that the relationship between the denominator and numerator is linear and that it passes through the origin. To investigate these assumptions, we performed regressions of orthogonal and parallel surround thresholds against no-surround thresholds[2]. As shown in Fig. R1 (left column) below, the relationships do appear to be linear but the y-intercept is consistently below zero.

However, as described by Allison, Paultre, Goran, Poehlman, and Heymsfield (1995), the most important aspect of the use of a ratio to control for the influence of the denominator is the extent to which the correlation between the ratio and the denominator approaches zero. As shown in Fig. R1 (right column), the relationship between the ratio and the no-surround threshold appears largely uncorrelated (all $|r_s| \leq 0.40$, all $p \geq 0.112$).

Conclusions

While the assumption of a zero y-intercept appears to not be satisfied in the data of Yoon et al. (2009), the lack of structure in the comparison of the ratios against the denominator suggests that this violation does not have appreciable effects within the range of the observed data.

[1] Note that the provided data contains a threshold value of 87.1, whereas the paper reports that all thresholds were clamped to <= 75. Changing this value to 75 permitted the reproduction of the results reported in the paper.

[2] Because both dimensions are measured with error, we performed regressions using the 'Model II' method described by Ludbrook (2012).

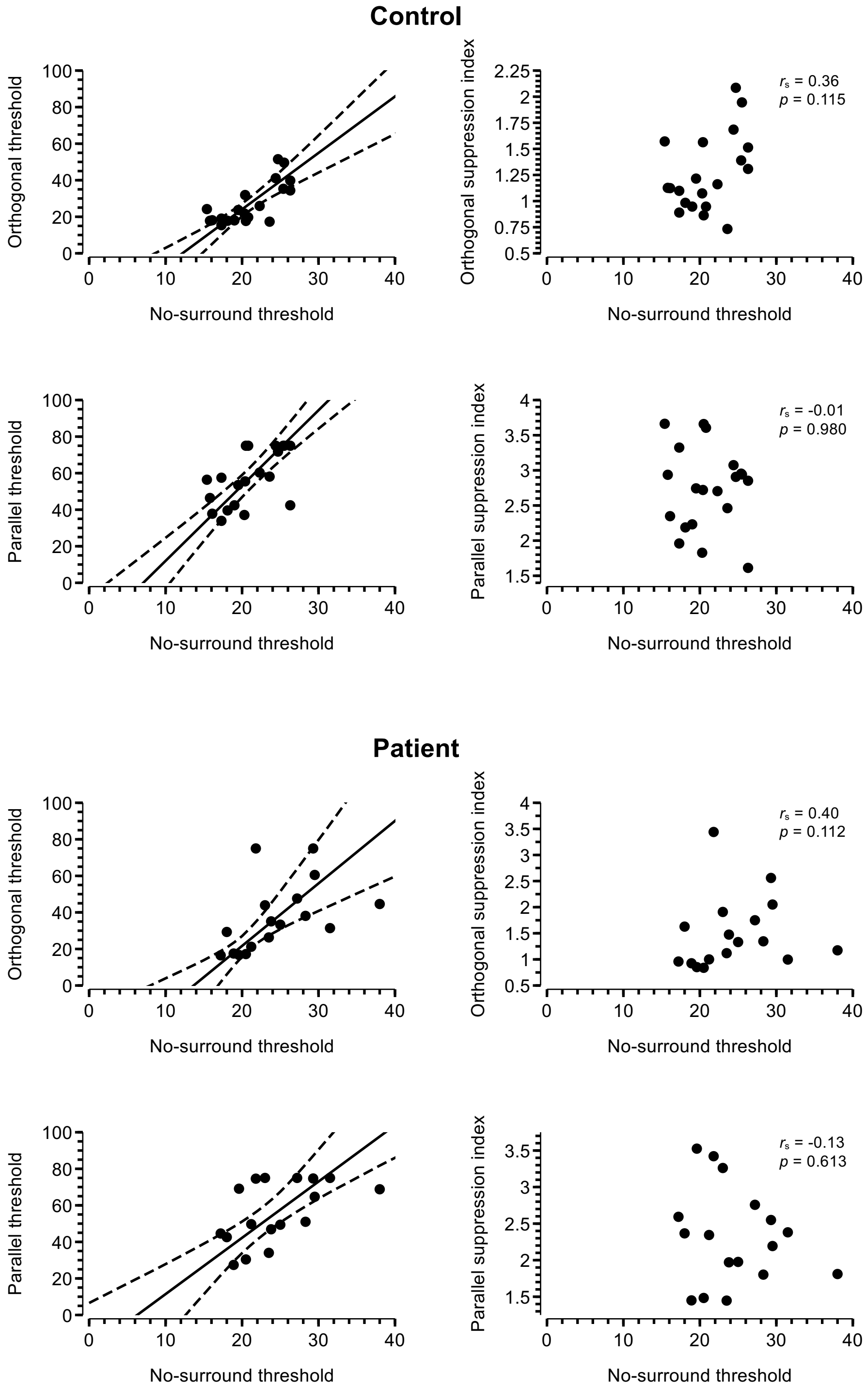

Fig. R1. Relationship between no-surround and oriented surround thresholds in Yoon et al. (2009). The top and bottom sections depict control and patient groups, respectively. The left column compare the thresholds in the no-surround condition (horizontal axis) with the thresholds in the articulated surround conditions (vertical axis). The line is the best-fitting regression, and the dashed line represents the 95% bootstrapped confidence intervals. The right column compares the thresholds in the no-surround condition (horizontal axis) with the surround suppression indices formed from the ratio of the articulated surround threshold to the no-surround threshold (vertical axis). The inset labels report the Spearman's correlation.

Serrano-Pedraza et al. (2014)

Key results of interest

Serrano-Pedraza et al. (2014) used a similar design to Yoon et al. (2009)—a two-way mixed design with group (control, patient) as a between-subjects factor and surround stimulus (none [NS], orthogonal [OS], parallel [PS]) as a within-subjects factor. Again, we are interested in the analyses in which the orthogonal and parallel conditions are expressed as a ratio of the no-surround condition ('suppression indices'). Note that, in contrast to Yoon et al. (2009), Serrano-Pedraza et al. (2014) analyse the logarithm of such ratios.

Check of ratio assumptions

As shown in Fig. R2 (left column), the y-intercept in the regression relating the no-surround and articulated surround conditions is typically close to zero (though with large uncertainty, particularly in the control group). However, we find that the relationship for the orthogonal conditions is such that there is structure evident in the comparison of the no-surround threshold with the logarithm of the orthogonal surround ratio. Quantification via Spearman's correlation indicates that the correlation coefficients for these conditions are significantly different from zero.

Conclusions

As a subset of the ratios show a clear relationship with the no-surround thresholds, we suggest that the interpretation of such ratios must proceed with caution.

References

Allison, D. B., Paultre, F., Goran, M. I., Poehlman, E. T., & Heymsfield, S. B. (1995). Statistical considerations regarding the use of ratios to adjust data. *International Journal of Obesity and Related Metabolic Disorders*, *19*(9), 644–652.

Ludbrook, J. (2012). A primer for biomedical scientists on how to execute Model II linear regression analysis. *Clin Exp Pharmacol Physiol*, *39*(4), 329–335.

Serrano-Pedraza, I., Romero-Ferreiro, V., Read, J. C. A., Diéguez-Risco, T., Bagney, A., Caballero-González, M., ... Rodriguez-Jimenez, R. (2014). Reduced visual surround suppression in schizophrenia shown by measuring contrast detection thresholds. *Frontiers in Psychology*, *5*, 1431.

Yoon, J. H., Rokem, A. S., Silver, M. A., Minzenberg, M. J., Ursu, S., Ragland, J. D., & Carter, C. S. (2009). Diminished orientation-specific surround suppression of visual processing in schizophrenia. *Schizophrenia Bulletin*, *35*(6), 1078–1084.

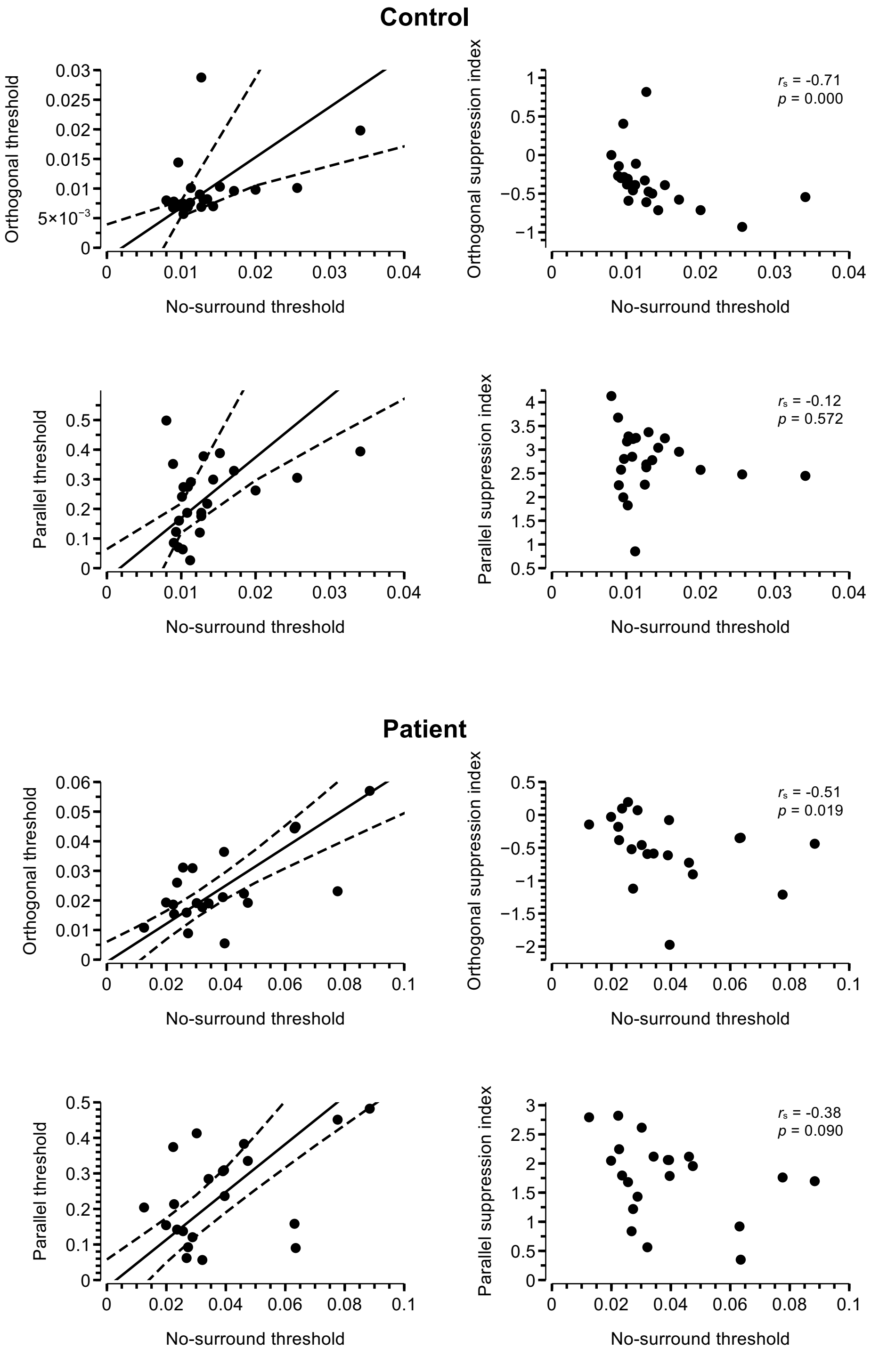

Fig. R2. Relationship between no-surround and oriented surround thresholds in Serrano-Pedraza et al. (2014). The format is as per Fig. R1, with the exception that log ratios are used to form the surround suppression indices.
